# C(sp)-C(sp) Lever-Based Targets of Orientational Chirality: Design and Asymmetric Synthesis

**DOI:** 10.3390/molecules29102274

**Published:** 2024-05-11

**Authors:** Ting Xu, Jia-Yin Wang, Yu Wang, Shengzhou Jin, Yao Tang, Sai Zhang, Qingkai Yuan, Hao Liu, Wenxin Yan, Yinchun Jiao, Xiao-Liang Yang, Guigen Li

**Affiliations:** 1 School of Chemistry and Chemical Engineering, Nanjing University, Nanjing 210093, China; 18751856695@163.com (T.X.); junfu23wang@163.com (Y.W.); 18851095810@163.com (S.J.); 2 Continuous Flow Engineering Laboratory of National Petroleum and Chemical Industry, School of Pharmacy, Changzhou University, Changzhou 213164, China; wjychem@cczu.edu.cn; 3 Department of Chemistry and Biochemistry, Texas Tech University, Lubbock, TX 79409-1061, USA; yao.tang@ttu.edu (Y.T.); zhangsai@cczu.edu.cn (S.Z.); qingyuan@ttu.edu (Q.Y.); h.liu@ttu.edu (H.L.); 4 Key Laboratory of Theoretical Organic Chemistry and Functional Molecular, Ministry of Education, School of Chemistry and Chemical Engineering, Hunan University of Science and Technology, Xiangtan 411201, China; 15273729085@163.com (W.Y.); yinchunjiao@hnust.edu.cn (Y.J.)

**Keywords:** orientational chirality, atropisomerism, Sonogashira coupling, *N*-sulfinylimine

## Abstract

In this study, the design and asymmetric synthesis of a series of chiral targets of orientational chirality were conducted by taking advantage of *N*-sulfinylimine-assisted nucleophilic addition and modified Sonogashira catalytic coupling systems. Orientational isomers were controlled completely using alkynyl/alkynyl levers [C(sp)-C(sp) axis] with absolute configuration assignment determined by X-ray structural analysis. The key structural element of the resulting orientational chirality is uniquely characterized by remote through-space blocking. Forty examples of multi-step synthesis were performed, with modest to good yields and excellent orientational selectivity. Several chiral orientational amino targets are attached with scaffolds of natural and medicinal products, showing potential pharmaceutical and medical applications in the future.

## 1. Introduction

Chirality and its asymmetric control have been among the most important and active topics in science and technology for over half a century because chirality phenomena widely exist in nature in varuous forms ranging from functional molecules to microscopic living organisms (e.g., helical bacteria) to macroscopic objects (e.g., sea shells) [1,2,3,4,5,6]. It is well known that chiral functional biomolecules, including peptides/proteins, DNA/RNA, and carbohydrates, play crucial roles in biological processes in human beings, animals, and plants [7,8,9,10]. This field has become even more important and active since an increasingly larger number of modern drugs, agrochemicals, clinical candidates, and their precursors involve chirality in their structures and subunits [11,12]. By changing the chirality of molecular medicine, potency and selectivity can be substantially enhanced to reduce dosages and unwanted side effects [11,12]. In modern materials science, controlling chirality has also been proven to be effective for achieving challenging photo- and optoelectronic properties [13,14,15,16,17,18]. It is worth noting that asymmetric synthesis and catalysis have been playing key roles in the discovery and development of new chiral drugs and materials for the past several decades [19,20,21,22,23,24,25,26,27,28,29,30,31,32,33].

**Figure 1:**
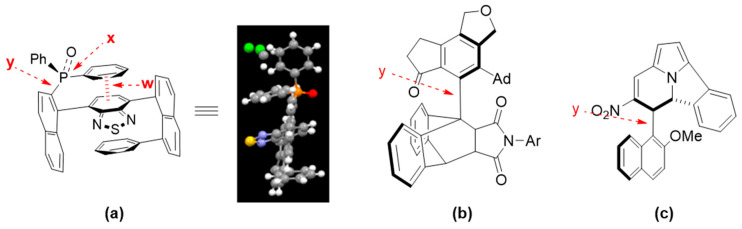
Targets (**a**–**c**) with pro-chiral center and orientational axis.

In general, chirality has been divided into the following categories so far: central [34,35,36], axial [23,37,38,39,40], spiral [41,42], sandwich (metallic [43,44] and organo [45,46]), multilayer (rigid helical [14] and flexible folding [47]), and inherent chirality [48]. Recently, we assembled a series of targets of multilayer folding chirality by using chiral auxiliaries and catalysts (Figure 1a) in which one single carbon–carbon bond forming led to multilayer chirality [49].

**Figure 2:**
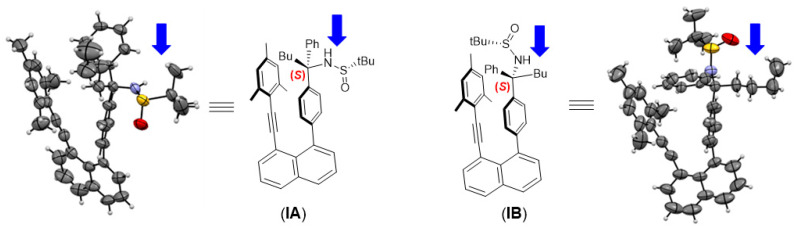
Two differentiated orientational isomers, (**IA**,**IB**).

A category of these chiral targets is stabilized by aromatic/aromatic interaction (w in Figure 1a) as proven by X-ray structures. In this chiral multilayer framework, a pro-chiral center (x in Figure 1a) and an orientational axis (y in Figure 1a) exist. The pro-phosphorus chiral center is directly connected to a naphthyl ring, and two phenyl groups are differentiated by parallel packing. The atropisomerism along the C-P axis is made possible jointly by Ar-Ar interaction and the parallel arrangement of phenyl ring on the bottom of the structural framework. Concurrently, the Sparr and Jørgensen labs independently reported an asymmetric catalytic approach to obtaining stable atropisomers containing C(sp^2^)−cyclized C(sp^3^) bonds as axes (Figure 1b,c) [50,51]. The atropisomeric chirality of their reports follows the well-known Felkin–Ahn-type model [52,53]. In this molecular framework, one of the three groups on C(sp^3^) is arranged perpendicularly to the C(sp^2^) plane. Very recently, we proposed the concept of orientational chirality and assembled its molecular architectures, in which chiral tetrahedron centers and blocking groups are anchored remotely through space [6,54,55] (Figure 2). In orientational chirality, two different rotamers were observed in their crystals, as revealed by X-ray diffraction analysis (Figure 2IA,IB). This work made it possible to achieve individual atropisomers centered on sp^3^ carbon with four independent open-chain and flexible motifs, which are differentiated from previous systems containing cyclized rigid substituents centered on sp^3^ carbon [54,55]. Among three open-chain orientational isomers, we selectively controlled one of them in which the amino branch is directed away from the remote anchor (Figure 2IA). As a result, the orientatiomers with the alkyl branches being forced away from the remote anchor were challenging in regard to the combinational design of two levers/arms on the planar naphthyl pier of the through-space structural framework. Herein, we would like to report our preliminary results of this asymmetric control.

## 2. Results and Discussion

### 2.1. Structural Design and Models

**Figure 3:**
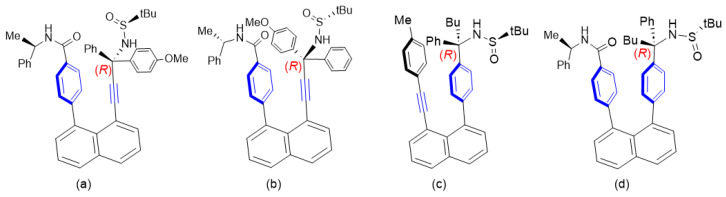
Orientational isomers (**a–d**) with different levers and remote blockers.

In our previous work, both phenyl and alkyl levers (blue units in Figure 3) were utilized as anchors for bringing remote blockers and sp^3^ chiral carbon centers closer, as represented by the orientational isomers in Figure 3 [6,54,55]. If two aromatic rings and amino branches are attached to sp^3^ chiral carbon centers with an alkynyl lever (C(sp)-C(sp^3^)) axis, the amino branch on the chiral carbon and one of the two individual aromatic branches are directed out of the plane, while another aromatic group remains on the plane and is selectively directed away from the remote chiral amide blocker on the phenyl lever [54,55]. In these cases, two chiral auxiliaries, carbonyl and sulfonyl amides, are necessary to jointly control the orientation of aromatic moieties (Figure 3a,b). A DFT computational study was performed on orientational individual orientatiomers in regard to their relative energy (Figure 3). Meanwhile, a rotational profile was also obtained by scanning the rotational dihedral angle θ, supporting the experimental observations in which the resulting orientatiomers are proven to be stable enough at constant temperatures and to be synthesized asymmetrically.

Interestingly, when one of two aromatic rings on the sp^3^ chiral carbon center was replaced by alkyl groups, and concurrently, the alkynyl and aryl levers were switched as [(C(sp^2^)-C(sp^2^)-right)/[(C(sp)-C(sp)-left) axes, the orientatiomers with amino branches directed away from the remote anchor were controlled selectively [6]. This situation exists due to the combinaion of aryl/aryl levers, the [(C(sp^2^)-C(sp^2^)-right)/[(C(sp^2^)-C(sp^2^)-left) axis, for remote blockers and the chiral carbon center [54,55]. For the latter oreintation chirality, DFT computation was also conducted, presenting relative stability with the rotation of individual orientatiomers. In order to selectively control the orientatiomers in which the alkyl branches are directed away from the remote anchor, we then focused on the use of two alkynyl levers, [(C(sp)-C(sp)-right/(C(sp)-C(sp)-left axis), for both the C(sp^3^) center and remote blockers. As shown by the asymmetric synthesis below, this design led to the results sought and is presented in forty examples.

**Figure 4:**
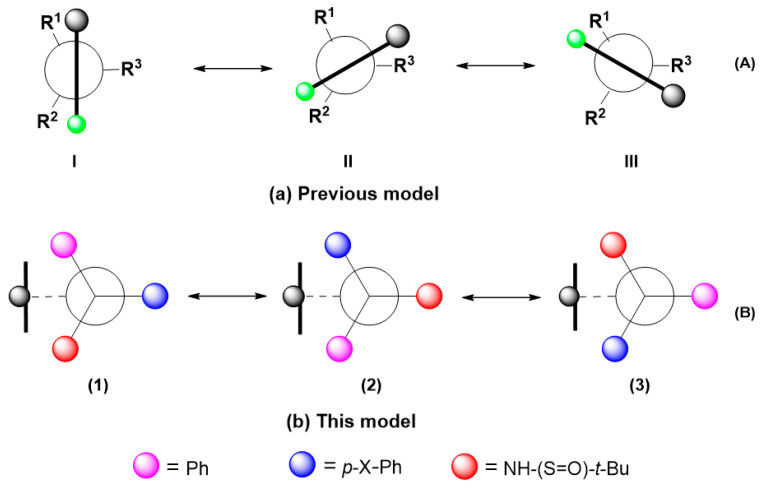
Felkin–Ahn (I–III) and orientational (1–3) chirality models.

Compared to the previous Felkin–Ahn-type model, the atropisomeric chirality is mainly based on the dialog relationship between two adjacent blocking C(sp^2^) and chiral C(sp^3^) scaffolds (I, II, III in Figure 4A). However, in orientational chirality cases in which [(C(sp)-C(sp)-right lever)/[C(sp^3^) stereogenic center] or [(C(sp^2^)-C(sp^2^)-right lever)/[C(sp^3^) stereogenic centre] structural combinations are shown, the remotely anchored groups block rotation along the C(sp^2^)−C(sp^3^) or C(sp^2^)−C(sp^3^) axis **(**Figure 4B), i.e., the new orientational chirality is focused on the dialog relationship between C(sp^3^) centers and remotely anchored chiral amide and aryl functional groups. Since there is a single interaction (the heavy black line in the model, Figure 4B) existing in each of the three orientatiomers, there are only three energy barriers instead of six existing in the previous atropisomerism. While the nomenclature of previous molecular frameworks follows the Cahn−Ingold−Prelog (CIP) rules, it seems difficult to find a nomenclature rule for the present chirality systems at this stage. The relationship among three orientatiomers ((1), (2) and (3) in Figure 4B) does not belong to the classical enantiomeric or diastereomeric isomerism. In both previous and present cases, there exist three pairs of enantiomers and six pairs of diastereomers, which is not often encountered in stereochemistry.

### 2.2. Retro-Synthetic Analysis (RSA)

**Scheme 1:**
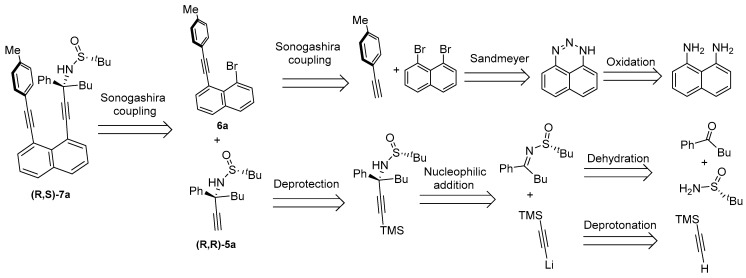
Retro-synthetic analysis of orientational target (**R**,**S**)-**7a**.

The structural assembly was performed by taking advantage of retro-synthetic analysis (RSA) with target **6a** as a representative [56], which mainly benefits from Sonogashira C-C coupling [57] and the chiral *N*-sulfinylimine auxiliary [58,59]. With the aim of facile access to the orientional isomers, we designed a convergent strategy wherein two fragments in Scheme 1, 1-bromo-8-(p-tolylethynyl)naphthalene **6a** and (*R*)-2-methyl-N-((R)-3-phenylhept-1-yn-3-yl)propane-2-sulfinamide (**R**,**R**)-**5a**, would be joined via dual Sonogashira couplings as the key C(sp)-C(sp^2^) bond formation step. 1-Bromo-8-(p-tolylethynyl)naphthalene **6a** can be synthesized by the first Sonogashira coupling between 1-ethynyl-4-methylbenzene and 1,8-dibromonaphthalene, which is pre-generated by reacting 1,8-diaminonaphthalene with NaNO_2_, followed by Sandmeyer reaction treatment with copper(I) bromide [60]. (*R*)-2-methyl-N-((R)-3-phenylhept-1-yn-3-yl)propane-2-sulfinamide (**R**,**R**)-**5a** is synthesized by the asymmetric addition of ((trimethylsilyl)ethynyl)lithium to (*R*,*E*)-2-methyl-N-(1-phenylpentylidene)propane-2-sulfinamide, which is obtained via a dehydration reaction of 1-phenylpentan-1-one with (*R*)-2-methylpropane-2-sulfinamide [61]. The cleavage of TMS is necessary because the trimethylsilyl precursor results in a complex product in this case, although the direct use of (trimethylsilyl)hept-1-yn-3-yl for Sonogashira is feasible in the literature [57].

### 2.3. Asymmetric Synthesis

**Scheme 2:**
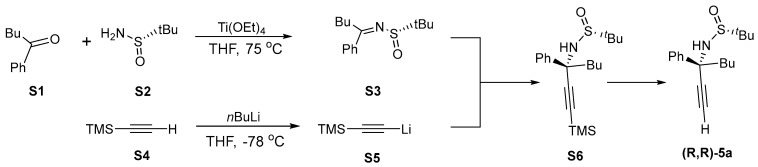
Asymmetric synthesis of (*R*)-*N*-(*R*)- building block (**R**,**R**)-**5a**.

**Scheme 3:**
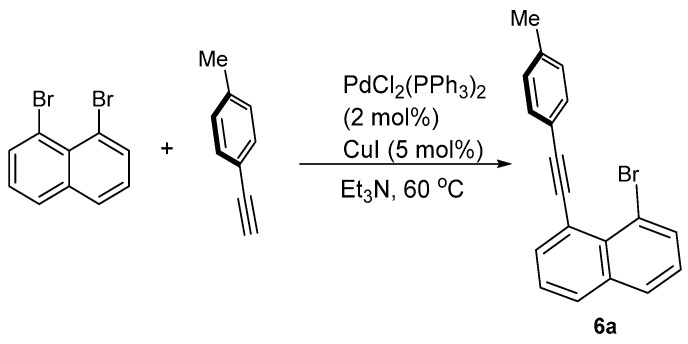
Synthesis of building block **6a**.

The asymmetric synthesis of orientatiomeric products **7** was also performed by assembling (*R*)-2-methyl-*N*-((*S*)-1-phenyl-1-(4-(8-(p-tolylethynyl)naphthalen-1-yl)phenyl)pentyl)propane-2-sulfinamide (**R**,**S**)-**7a** via two building blocks, (R)-2-methyl-N-((R)-3-phenylhept-1-yn-3-yl)propane-2-sulfinamide (**R**,**R**)-**5a** and 1-bromo-8-(p-tolylethynyl)naphthalene **6a** (Scheme 2). The preparation of the first building block **S3** was performed by the dehydration of 1-phenylpentan-1-one (**S1**) with (*R*)-2-methylpropane-2-sulfinamide (**S2**) by using Ti(OEt)_4_ in dry THF, at 75 °C to room temperature, to give a 93% yield [60]. The resulting *N*-sulfonyl ketimine (**S3**) was treated by ((trimethylsilyl)ethynyl)lithium (**S5**), which was pre-generated from the deprotonation of ethynyltrimethylsilane (**S4**) by *n*BuLi in THF, at −78 °C, to produce (*S*)-*N*-((*R*)-1-(butyl)-1-phenyl-3-(trimethylsilyl)prop-2-yn-1-yl)-2-methylpropane-2-sulfinamide ((**R**,**R**)-**5a**. The deprotection of **S6** was performed by treating (R)-2-methyl-N-((S)-3-phenyl-1-(trimethylsilyl)hept-1-yn-3-yl)propane-2-sulfinamide with K_2_CO_3_ in the presence of MeOH, with an overall yield of 82%. The preparation of the second building block, 1-bromo-8-(p-tolylethynyl)naphthalene (**6a**), was constructed by subjecting 1,8-dibromonaphtalene to a Sonogashira coupling reaction with 1-ethynyl-4-methylbenzene in the presence of PdCl_2_(PPh_3_)_2_ and CuI as co-catalysts in Et_3_N solution to produce 1-bromo-8-(p-tolylethynyl)naphthalene (**6a**) in a 74% chemical yield (Scheme 3).

**Scheme 4:**
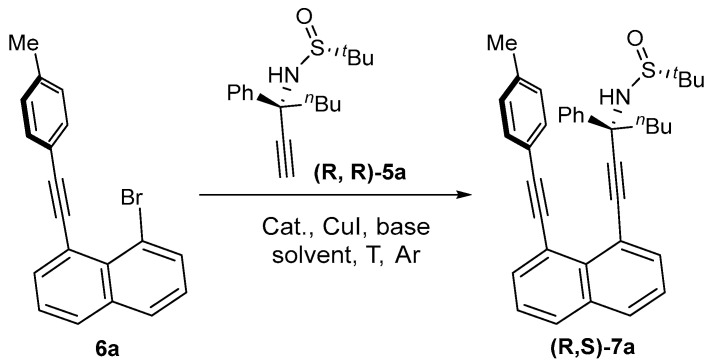
Assembly of final orientational isomer (**R**,**S**)-**7a**.

Surprisingly, when we conducted the final assembly of the final orientatiomeric product under Sonogashira and the previous modified catalytic coupling systems (Scheme 4), we found that all known conditions did not work well. We thus investigated various catalysts which have been commonly used in previous coupling reactions. Among them, NiCl_2_(PPh_3_)_2,_ PdCl_2_ and Pd(OAc)_2_ did not provide the target product at all; rather, the raw materials were recovered almost quantitatively (Table 1, entries 1, 3, and 5). PdCl_2_(PPh_3_)_2_ produced the product ((**R**,**S**)-**7a**) in a 45% yield (Table 1, entry 2), which highlights the advantages of palladium catalysis. A dramatically lower yield of 28% was obtained when Pd(PPh_3_)_4_ (Table 1, entry 4) was employed, and PdCl_2_(PPh_3_)_2_ is proven to be more appropriative than PdCl_2_(dppf)_2_ (Table 1, entry 6) for this step. Several solvents, including THF and DMF, were subsequently screened (Table 1, entries 7–9), while Cs_2_CO_3_ or Et_3_N was used as the base, with inferior results obtained in each case. In addition, we found that changing temperatures showed a significant impact on chemical yield (Table 1, entries 10–11). The optimal conditions for this reaction are PdCl_2_(PPh_3_)_2_ (2 mol %) and CuI (5 mol %) in Et_3_N as both a base and a solvent under argon at 50 °C for 24 h (Table 1, entries 10), leading to a yield of 68%. Control experiments were used to demonstrate that PdCl_2_(PPh_3_)_2_ and CuI were found to be crucial to this modified Sonogashira cross-coupling (Table 1, entry 12).

**Scheme 5:**
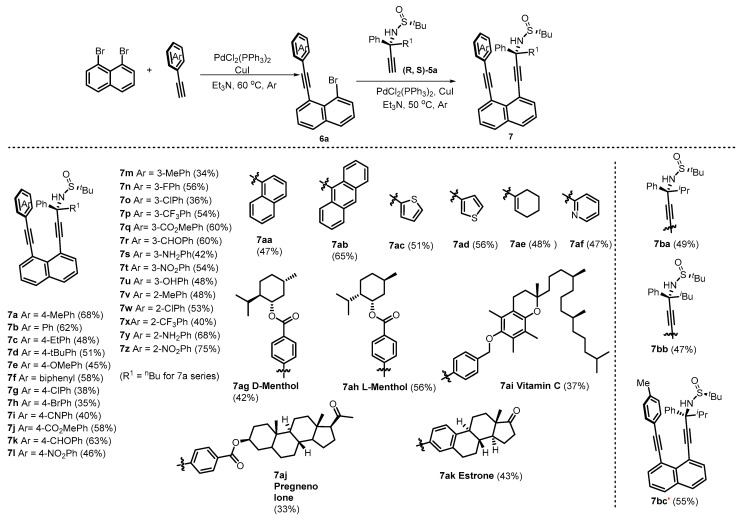
Scope of substrate expansion.

Having established the optimized conditions, we synthesized a series of orientational isomeric products by varying aryl alkyne substrates **6** with the results listed in Scheme 5. With the use of PdCl_2_(PPh_3_)_2_ (2 mol %) and CuI (5 mol %), substrates **6** bearing various aliphatic substituents underwent coupling reactions with chiral alkynyl precursors (**R**,**R**-**5a**), with moderate yields from 34% to 68%, showing that the electronic and steric nature of the aryl group on the benzyl acrylate appear to influence the reaction. Heteroaromatic rings, such as thiophene and pyridine ((**R**,**S**)-**7ac**, **7ad**, and **7af**), are especially compatible with this synthesis, and the yields range from 47% to 56%. For the cases of (**R**,**S**)-**7ba** and (**R**,**S**)-**7bb**, in which the n-butyl group in (**R**,**R**)-**5a** was replaced with isopropyl and isobutyl groups, respectively, stable rotamers were shown as expected. The opposite configuration of building block (**S**,**S**)-**5a** can also be smoothly converted into the corresponding orientational isomer (**S**,**R**)-**7bc′** with a chemical yield of 55%.

It is worth noting that several of the resulting orientational amino targets are attached with scaffolds of natural and medicinal products, including D-menthol, L-menthol, vitamin C, pregnenolone, and estrone (**R**,**S**)-**7ag**–(**R**,**S**)-**7ak,** with moderate yields and a single orientational configuration under these conditions. This shows their potential for pharmaceutical and medicinal applications in the future, especially for amino acid- and peptide-derivatized targets when the aliphatic or aromatic branch is replaced by a carboxylic acid group.

**Scheme 6:**
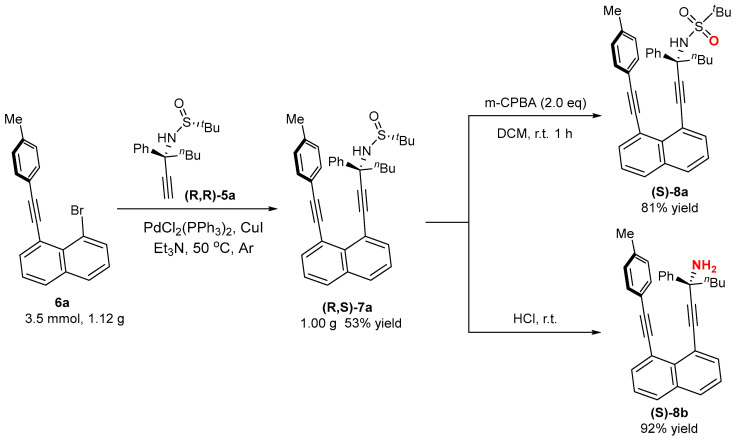
Further transformations of orientational products.

A scale-up experiment was performed to produce (**R**,**S**)-**7a** in a 53% yield (Scheme 6). (**R**,**S**)-**7a** can be readily converted to (S)-2-methyl-N-(3-phenyl-1-(8-(p-tolylethynyl)naphthalen-1-yl)hept-1-yn-3-yl)propane-2-sulfonamide ((**S**)-**8a**) through treatment with m-chloroperoxybenzoic acid (m-CPBA) in an 81% yield. Meanwhile, the *tert*-butylsulfinyl group of (**R**,**S**)-**7a** can be cleaved using hydrochloric acid to give (S)-3-phenyl-1-(8-(p-tolylethynyl)naphthalen-1-yl)hept-1-yn-3-amine ((S)-**8b**) in a 92% yield.

**Figure 5:**
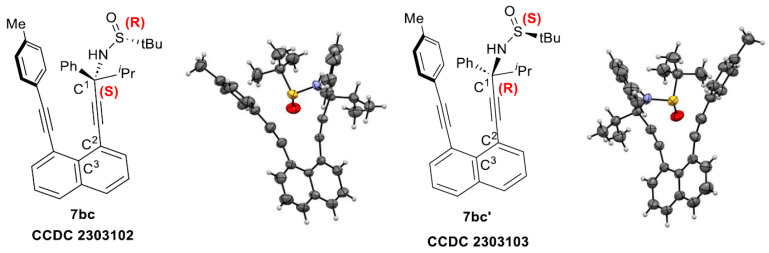
Orientational isomers confirmed by X-ray diffraction analysis. Bond angles of N-C1-C2-C3 in ORTEP are −96.2° and 95.9°, respectively.

The absolute orientational configuration was unambiguously confirmed by the X-ray diffraction analysis of (*R*)-2-methyl-*N*-((*R*)-1-phenyl-1-(4-(8-(p-tolylethynyl)naphthalen-1-yl)phenyl)pentyl)propane-2-sulfinamide (**R**,**S**)-**7bc** and its enantiomer (Figure 5). In these two structures, *i*-propyl is clearly shown to be pushed away by the remote controller on its backside. This structural arrangement of two alkynyl/alkynyl levers is different from that of alkynyl/aryl levers in which the amino branch on the chiral carbon is directed away from the remote blocker. The latter case is similar to that of aryl/aryl lever-based orientational configuration with an alkyl group on the chiral carbon center. However, the present case shows a similar orientation in which the amino branch is pointed away from the plane (Figure 5).

## 3. Computational and VT NMR Studies

**Figure 6:**
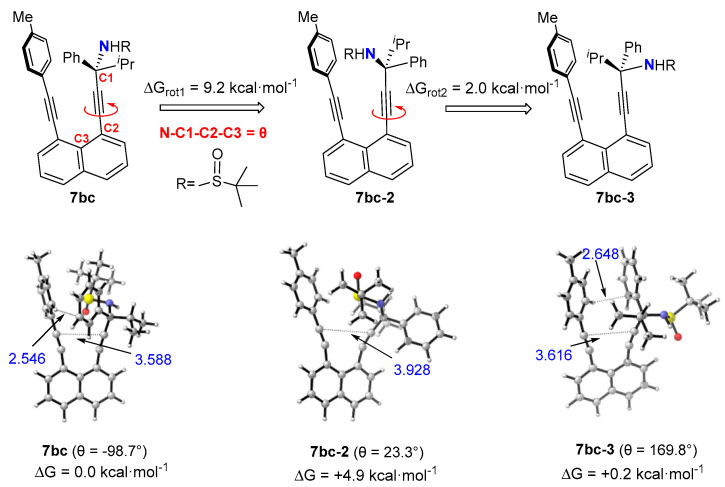
Optimized conformers and relative energies of orientatiomer (**R**,**S**)-**7bc**. The method is at the B3LYP-D3(BJ)/6–311++G (d,p) (SMD, triethylamine)//B3LYP-D3(BJ)/6–31G (d,p) level of theory (distances are in Å; H, white; C, gray; N, blue; O, red; S, yellow). The rotational energy barrier of the reactant was calculated: ΔG_rot1_ = 9.2 kcal·mol^−1^ and ΔG_rot2_ = 2.0 kcal·mol^−1^.

All DFT calculations were performed with the Gaussian 16 package [62]. The geometry optimizations of minima were carried out for the B3LYP-D3(BJ) functional [63,64] and the 6–31G(d,p) [65,66] basis sets. The vibrational frequencies were computed at the same level to check whether each optimized structure was an energy minimum (zero imaginary frequency) and to evaluate its zero-point vibration energy (ZPE) and thermal corrections at 323.15 K in kcal‧mol^−1^. The single-point energies and solvent effects in triethylamine (*ε* = 2.3832) were computed with the B3LYP-D3(BJ) function and the 6–311++G(d,p) basis set using the SMD solvation model [67,68]. The DFT-optimized structures were illustrated using CYLView (Figure 6) [69,70].

**Figure 7:**
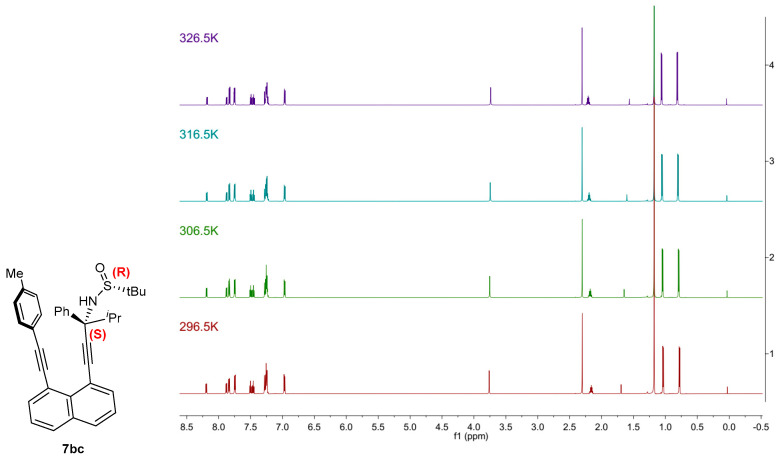
^1^H VT-NMR spectra of **7bc** in CDCl_3_ (more spectra are provided in SI).

We also conducted variable temperature NMR (VT NMR) experiments to explore the stability of orientational isomer **7bc** (Figure 7). The variable temperature spectra were acquired in 10-degree increments in the range of 25 °C to 55 °C. As shown in Figure 7 and the Supplementary Materials (pp SI 76), after undergoing VT-NMR, we can clearly see that the chemical shifts in protons and carbons in all corresponding functionality remain consistent. This indicates that the structure shown in the **7bc** is a stable isomer within this temperature range.

## 4. Conclusions

We conducted the design and asymmetric synthesis of a series of chiral targets of orientational chirality by taking advantage of *N*-sulfinylimine-assisted nucleophilic addition and modified Sonogashira coupling catalysis. Orientational isomers were readily controlled through alkynyl/alkynyl levers [C(sp)-C(sp) axis], and unambiguously determined by X-ray structural analysis. The key structural element of the resulting orientational chirality is characterized by remote through-space blocking. Forty examples of multi-step synthesis were performed, exhibiting modest to good yields and excellent orientational selectivity. It is worth noting that several of the resulting orientational amino targets are attached with scaffolds of natural and medicinal products, including D-menthol, L-menthol, vitamin C, pregnenolone, and estrone, showing their potential for pharmaceutical and medicinal applications in the future, especially for amino acid- and peptide-derivatized targets when the aliphatic or aromatic branches are replaced by carboxylic acid groups. Further physical organic chemistry studies of new orientational compounds will be conducted in due course [50,51,52,53,71].

## Figures and Tables

**Table 1:** Optimization of the reaction conditions.

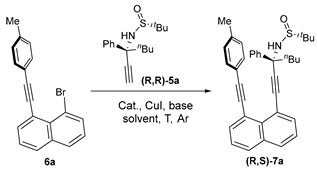					
Entry ^a^	Cat	Base	Solvent	Temp.	Product (%) ^b^
1	NiCl_2_(PPh_3_)_2_	-	Et_3_N	60	n. r.
2	PdCl_2_(PPh_3_)_2_	-	Et_3_N	60	45
3	PdCl_2_	-	Et_3_N	60	n. r.
4	Pd(PPh_3_)_4_	-	Et_3_N	60	28
5	Pd(OAc)_2_	-	-	60	n. r.
6	PdCl_2_(dppf)_2_	-	-	60	42
7	PdCl_2_(PPh_3_)_2_	Et_3_N	THF	60	Trace
8	PdCl_2_(PPh_3_)_2_	Cs_2_CO_3_	THF	60	37
9	PdCl_2_(PPh_3_)_2_	Et_3_N	DMF	60	42
**10 ^c^**	**PdCl_2_(PPh_3_)_2_**	-	**Et_3_N**	**50**	**68**
11	PdCl_2_(PPh_3_)_2_	-	Et_3_N	70	52
12	-	-	Et_3_N	60	n. r.

^a^ Reaction conditions: Reactions were carried out with **6a** (64 mg, 0.20 mmol), (**R**,**R**)-**5a** (59.2, 0.20 mmol), cat. (5 mol %), CuI (10 mol %), and a base (0.6 mmol, 3.0 eq) in solvent (2.0 mL) under argon for 24 h. ^b^ Isolated yields of (**R**,**S**)-**7a** after purification by column chromatography. ^c^ Reaction was carried out with PdCl_2_(PPh_3_)_2_ (2 mol %) and CuI (5 mol %) under argon for 24 h. n. r. represents no reaction.

## Data Availability

The data presented in this study are available in article and Supplementary Materials.

## Supplementary Materials

The following supporting information can be downloaded at: https://www.mdpi.com/article/10.3390/molecules29102274/s1.
