# Supplementary material for: C(sp)-C(sp) Lever-Based Targets of Orientational Chirality: Design and Asymmetric Synthesis
Source: Molecules. 2024 May 11;29(10):2274. doi: 10.3390/molecules29102274 (PMC11123770; doi:10.3390/molecules29102274)
Supplement: Supplementary file 1 [file molecules-29-02274-s001.zip › molecules-2988739-supplementary.pdf]

Supporting Information

C(sp)-C(sp) Lever-Based Targets of Orientational Chirality: Design and Asymmetric Synthesis

Ting Xu 1, Jia-Yin Wang 2, Yu Wang 1, Shengzhou Jin 1, Yao Tang 3, Sai Zhang 3, Qingkai Yuan 3, Hao Liu 3, Wenxin Yan 4, Yinchun Jiao 4, Xiao-Liang Yang 1,* and Guigen Li 1,3,*

1 School of Chemistry and Chemical Engineering, Nanjing University, Nanjing 210093, China; 18751856695@163.com (T.X.); junfu23wang@163.com (Y.W.); 18851095810@163.com (S.J.)

2 Continuous Flow Engineering Laboratory of National Petroleum and Chemical Industry, School of Pharmacy, Changzhou University, Changzhou 213164, China; wjychem@cczu.edu.cn

3 Department of Chemistry and Biochemistry, Texas Tech University, Lubbock, TX 79409-1061, USA; yao.tang@ttu.edu (Y.T.); zhangsai@cczu.edu.cn (S.Z.); qingyuan@ttu.edu (Q.Y.); h.liu@ttu.edu (H.L.)

4 Key Laboratory of Theoretical Organic Chemistry and Functional Molecular, Ministry of Education, School of Chemistry and Chemical Engineering, Hunan University of Science and Technology, Xiangtan 411201, China; 15273729085@163.com (W.Y.); yinchunjiao@hnust.edu.cn (Y.J.)

* Correspondence: yxlnmr@nju.edu.cn (X.-L.Y.); guigen.li@ttu.edu (G.L.)

Context

1. Synthetic Procedures and Characterization Data S2-S27

2. NMR Spectra of Precursors **A3**, **A4**, **B3**, **C5** and **D5**..S28-S32

3. NMR Spectra of Compounds **7a-8b**...S33-S75

4. VT NMR of Compound **7bc** S76

5. X-ray Single-crystal Data for Compound **7bc** and **7bc'** S77-S79

EXPERIMENTAL SECTION

For general Information, all melting points are uncorrected. The NMR spectra were recorded in $CDCl_3$ on a 400 MHz instrument with TMS as the internal standard. Chemical shifts (δ) are reported in ppm with respect to TMS. Data are represented as follows: chemical shift, multiplicity (s = singlet, d = doublet, t = triplet, m = multiplet), coupling constant (J, Hz), and integration. High-resolution mass spectrometry (HRMS) analyses were carried out using a time-of-flight mass spectrometry (TOF-MS) instrument with an electrospray ionization (ESI) source. X-ray crystallographic analysis was performed with a SMART CCD and a P4 diffractometer. All commercially sourced starting materials were used without further purification.

1. General Procedure for the Synthesis of alkynyl precursors 5

The product of **(R,Z)-2-methyl-N-(1-phenylpentylidene)propane-2-sulfinamide (S3)**: was synthesized according to the literature (**Scheme 2**). Ethynyltrimethylsilane was converted into ((trimethylsilyl)ethynyl)lithium precursor *via* the treatment with ^{n}BuLi in THF at -78 ^{o}C, followed by reacting with (**S5**) to give **(R)-2-methyl-N-((S)-3-phenyl-1-(trimethylsilyl)hept-1-yn-3-yl)propane-2-sulfinamide** (**S6**, 48% yield), which was then transformed into **(R)-2-methyl-N-((R)-3-phenylhept-1-yn-3-yl)propane-2-sulfinamide** (**(R,R)-5a**) by reacting with K_2CO_3 in the presence of MeOH to give a yield of 65%.

2. General Procedure for the Synthesis of Products (R,S-7)

1-bromo-8-(p-tolylethynyl)naphthalene (**6a**) was prepared by Sonogashira coupling in 74 % chemical yield (**Scheme 3**). With the abovementioned assumption in mind, we explored the reaction of **1-bromo-8-(p-tolylethynyl)naphthalene** (**6a**) (0.1 mmol) with **(R)-2-methyl-N-((R)-3-phenylhept-1-yn-3-yl)propane-2-sulfinamide** (**(R,R)-5a**) (0.1 mmol, 1.0 eq), as model substrates, with $PdCl_2(PPh_3)_2$ (2 mol%) and Cu(I) iodide (5 mol%) as co-catalysts in the presence of Et_3N (2.0 mL), promoted under argon at 50 ^{o}C for 24 h, resulted in the product **(R)-2-methyl-N-((S)-3-phenyl-1-(8-(p-tolylethynyl)naphthalen-1-yl)hept-1-yn-3-yl)propane-2-sulfinamide** (**(R,S)-7a**) in a yield of 68% (**Scheme 5**).

3. General Procedure for the Synthesis of substrates 6

The substrates **6** were prepared according to the reported procedures. Compound **A3, A4, B3, C5** and **D5** are new, while the rest of them have been previously reported, and their characterization data are in agreement with the literature.

A3:

Step 1: A 25 mL clean and dried tube was equipped with a stirring bar and charged with (1S, 2S, 5R)-2-isopropyl-5-methylcyclohexan-1-ol **A1** (3.0 mmol), 4-ethynylbenzoic acid (3.0 mmol, 1.0 eq.) and DMAP (0.6 mmol, 0.2 eq). 15 mL of DCM was then added to the mixture. DCC (3.6 mmol, 1.2 eq) was added slowly to this solution at 0 °C. Next, the mixture was stirred at 25 °C overnight until the reaction completed. After concentration, the crude product was purified *via* flash chromatography on silica gel using PE/EA as an eluent to give the final product **A2** (88% yield, 0.748 g).

Step 2: A mixture of **A2** (2.5 mmol), 1,8-dibromonaphthalene (2.5 mmol, 1.0 eq) and $Pd(PPh_3)_2Cl_2$ (2 mol %), CuI (5 mol%) in 20 mL Et_3N was stirred for 12 h at 60 °C under argon atmosphere. The resulting mixture was extracted with 50 mL H_2O and 20 mL ethyl acetate for three times. The organic phase was concentrated to a bottle and was dried over $MgSO_4$. Then, the mixture was filtrated. The crude product was purified by chromatography on silica gel to afford compound **A3 (**total 48% yield, 0.703 g). (Figure S1-2 in the Supporting Information)

Product **A4 (**total 41% yield, 0.605 g) was obtained through the synthesis method of product **A3**. (Figure S3-4 in the Supporting Information)

B3:

B3 was obtained through the synthesis method of product **A3 (**total 36% yield, 0.70 g). (Figure S5-6 in the Supporting Information)

C3:

Step 1: **C1** (5 mmol) was dissolved in 20 mL DCM and the mixture was loaded in an oven-dried flask equipped with a magnetic stir bar. Then, pyridine (10 mmol, 2.0 eq) was added to the flask and the mixture was cooled to 0 °C. Subsequently, Tf_2O (6 mmol, 1.2 eq) was added dropwise to the mixture at 0 °C and the mixture was stirred under room temperature overnight. The mixture was then quenched with NH_4Cl and extracted with DCM. The organic layer was dried over anhydrous $MgSO_4$ and filtered. The colature was evaporated on a rotary evaporator. The crude product was purified by chromatography on silica gel to afford compound **C2** (88% yield, 1.762 g).

Step 2: A mixture of **C2** (4 mmol), ethynyltrimethylsilane (5.6 mmol, 1.4 eq), triethylamine (4 mL), and $Pd(PPh_3)_2Cl_2$ (3 mol %) in 20 mL DMF was stirred for 4 h at 90 °C under argon atmosphere. The resulting mixture was extracted with 50 mL H_2O and 20 mL ethyl acetate three times. The organic phase was concentrated to a bottle and was dried over $MgSO_4$. Then, the mixture was filtrated. The crude product was purified by chromatography on silica gel to afford compound **C3** (63% yield, 0.879 g).

Step 3: **C3** (2 mmol) was dissolved in 10 mL MeOH and K_2CO_3 (3 mmol, 1.5 eq) was loaded in the mixture, which was stirred at room temperature for 8 h. The reaction process was determined by TLC until the starting material **C3** consumed completely. The mixture was quenched by saturated ammonium chloride solution and extracted with ethyl acetate for three times. The crude product was purified by chromatography on silica gel to afford compound **C4** (47% yield, 0.263 g).

C5 was obtained through the synthesis method of product **A3 (**total 25% yield, 0.602 g)**.** (Figure S7-8 in the Supporting Information).

D5:

Step 1: D1 (7.5 mmol, 1.5 eq) and Cs_2CO_3 (10 mmol, 2.0 eq) were dissolved in DMF. Then the 1-(bromomethyl)-4-iodobenzene (5 mmol, 1.0 eq) was added to the reaction vial and the reaction was reacted at 60 °C overnight. The resulting mixture was cooled to room temperature and filtered. Then the filtrate was extracted with ethyl acetate

and saturated aqueous sodium chloride. The organic phase was concentrated, dried by $MgSO_4$ and filtered. The crude product was purified by chromatography on silica gel to afford compound **D2** (77% yield, 0.702 g).

D5 was obtained through the synthesis method of product **A3** (total 21% yield, 0.989 g). (Figure S9-10 in the Supporting Information)

(1S, 2S, 5R)-2-isopropyl-5-methylcyclohexyl 4-((8-bromonaphthalen-1-yl)ethynyl)benzoate (A3)

^{1}H NMR (400 MHz, $CDCl_3$) δ 8.06 (dd, *J* = 15.0, 8.4 Hz, 2H), 7.98 - 7.84 (m, 4H), 7.65 (dd, *J* = 27.2, 8.4 Hz, 2H), 7.53 - 7.45 (m, 1H), 7.32 (dd, *J* = 15.2, 7.6 Hz, 1H), 5.00 - 4.93 (m, 1H), 2.20 - 2.13 (m, 1H), 2.02 - 1.94 (m, 1H), 1.76 (d, *J* = 12.0 Hz, 2H), 1.61 (d, *J* = 11.6 Hz, 2H), 1.20 - 1.10 (m, 2H), 0.98 - 0.93 (m, 7H), 0.85 - 0.81 (m, 3H). ^{13}C NMR (100 MHz, $CDCl_3$) δ 165.6, 136.1, 135.7, 135.4, 134.1, 132.4, 130.7, 130.4, 130.2, 129.6, 129.2, 128.5, 126.6, 126.5, 125.6, 120.5, 96.9, 93.0, 75.1, 47.3, 41.0, 34.3, 31.5, 26.6, 23.7, 22.0, 20.8, 16.6.

(1S,2R,5S)-2-isopropyl-5-methylcyclohexyl 4-((8-bromonaphthalen-1-yl)ethynyl)benzoate (A4)

^{1}H NMR (400 MHz, $CDCl_3$) δ 8.06 (dd, *J* = 15.2, 8.4 Hz, 2H), 7.98 - 7.84 (m, 4H), 7.65 (dd, *J* = 27.2, 8.4 Hz, 2H), 7.53 - 7.45 (m, 1H), 7.32 (dd, *J* = 15.6, 7.6 Hz, 1H), 5.00 - 4.93 (m, 1H), 2.20 - 2.13 (m, 1H), 2.02 - 1.94 (m, 1H), 1.76 (d, *J* = 11.6 Hz, 2H), 1.61 (d, *J* = 11.6 Hz, 2H), 1.20 - 1.10 (m, 2H), 0.98 - 0.93 (m, 7H), 0.85 - 0.81 (m, 3H). ^{13}C NMR (100 MHz, $CDCl_3$) δ 165.6, 136.1, 135.7, 135.4, 134.1, 132.4, 130.7, 130.4, 130.2, 129.6, 129.2, 128.5, 126.6, 126.5, 125.6, 120.5, 96.9, 93.0, 75.1, 47.3, 41.0, 34.3, 31.5, 26.6, 23.7, 22.0, 20.8, 16.6.

(3S,8S,9S,10R,13S,14S,17S)-17-acetyl-10,13-dimethyl-2,3,4,7,8,9,10,11,12,13,14,15,16,17-tetradecahydro-1H-cyclopenta[a]phenanthren-3-yl 4-((8-bromonaphthalen-1-yl)ethynyl)benzoate (B5)

^{1}H NMR (400 MHz, $CDCl_3$) δ 8.08 (d, *J* = 8.4 Hz, 2H), 7.97 - 7.82 (m, 4H), 7.72 - 7.64 (m, 2H), 7.52 - 7.47 (m, 1H), 7.36 - 7.30 (m, 1H), 5.46 (d, *J* = 3.6 Hz, 1H), 4.94 - 4.85 (m, 1H), 2.61 - 2.46 (m, 3H), 2.15 (s, 3H), 2.11 -

1.94 (m, 4H), 1.80 - 1.50 (m, 9H), 1.33 - 1.16 (m, 4H), 1.10 (s, 3H), 0.67 (s, 3H). ^{13}C NMR (100 MHz, $CDCl_3$) δ 209.4, 165.5, 139.6, 136.1, 135.7, 134.1, 130.6, 130.4, 130.1, 129.5, 129.2, 128.6, 126.6, 125.6, 122.5, 120.5, 100.0, 96.9, 93.1, 74.7, 63.7, 56.9, 50.0, 44.0, 38.8, 38.2, 37.1, 36.7, 31.9, 31.8, 31.5, 27.9, 24.5, 22.9, 21.1, 19.4, 13.2.

(8R,9S,13S,14S)-3-((8-bromonaphthalen-1-yl)ethynyl)-13-methyl-6,7,8,9,11,12,13,14,15,16-decahydro-17H-cyclopenta[a]phenanthren-17-one (C5)

^{1}H NMR (400 MHz, $CDCl_3$) δ 7.93 - 7.88 (m, 2H), 7.83 (d, *J* = 8.4 Hz, 2H), 7.49 - 7.41 (m, 2H), 7.37 (s, 1H), 7.34 - 7.29 (m, 2H), 2.99 - 2.93 (m, 2H), 2.57 - 2.44 (m, 2H), 2.39 - 2.32 (m, 1H), 2.20 - 2.00 (m, 4H), 1.69 - 1.48 (m, 7H), 0.95 (s, 3H). ^{13}C NMR (100 MHz, $CDCl_3$) δ 140.3, 136.7, 135.7, 135.6, 133.9, 131.3, 130.6, 129.7, 129.1, 128.3, 126.4, 125.6, 125.4, 121.5, 121.2, 120.7, 97.9, 89.6, 50.6, 48.0, 44.5, 38.0, 35.8, 31.6, 29.2, 26.4, 25.6, 21.6, 13.9.

(S)-6-((4-((8-bromonaphthalen-1-yl)ethynyl)benzyl)oxy)-2,5,7,8-tetramethyl-2-((4S,8S)-4,8,12-trimethyltridecyl)chromane (D5)

^{1}H NMR (400 MHz, $CDCl_3$) δ 7.94 - 7.88 (m, 2H), 7.84 - 7.80 (m, 2H), 7.65 (d, *J* = 8.0 Hz, 2H), 7.52 (d, *J* = 8.0 Hz, 2H), 7.46 (t, *J* = 7.6 Hz, 1H), 7.32 - 7.28 (m, 1H), 4.74 (s, 2H), 2.64 - 2.58 (m, 2H), 2.23 (s, 3H), 2.18 (s, 3H), 2.13 (s, 3H), 1.87 - 1.76 (m, 2H), 1.60 - 1.51 (m, 4H), 1.47 - 1.26 (m, 12H), 1.23 - 1.04 (m, 8H), 0.89 - 0.85 (m, 12H). ^{13}C NMR (100 MHz, $CDCl_3$) δ 148.1, 148.0, 138.3, 135.7, 134.0, 131.0, 130.7, 129.9, 129.1, 127.9, 127.6, 126.5, 125.9, 125.6, 123.5, 123.0, 121.0, 120.7, 117.7, 97.7, 90.3, 74.9, 74.3, 40.1, 39.4, 37.5, 37.5, 37.4, 37.3, 32.8, 32.7, 31.e, 28.0, 24.8, 24.5, 23.9, 22.7, 22.6, 21.1, 20.7, 19.8, 19.7, 12.9, 12.0, 11.8.

(R)-2-methyl-N-((S)-3-phenyl-1-(8-(p-tolylethynyl)naphthalen-1-yl)hept-1-yn-3-yl)propane-2-sulfinamide (7a)

Isolation by column chromatography (petroleum ether/ ethyl acetate= 5/1 v/v), white solid, 36.2 mg, 68 % yield, mp 138.4-139.5 °C; ^{1}H NMR (400 MHz, $CDCl_3$) δ 8.03 (d, *J* = 7.2 Hz, 1H), 7.84 - 7.79 (m, 3H), 7.70 (d, *J* = 7.6 Hz, 2H), 7.49 - 7.41 (m, 2H), 7.32 - 7.26 (m, 3H), 7.14 (d, *J* = 8.0 Hz, 2H), 6.90 (d, *J* = 8.0 Hz, 2H), 3.52 (s, 1H), 2.27 (s, 3H), 2.18 - 2.10 (m, 1H), 1.87 - 1.80 (m, 1H), 1.74 - 1.68 (m, 1H), 1.48 - 1.42 (m, 1H), 1.18 (s, 9H), 1.11 (d, *J* = 6.8 Hz, 1H), 0.99 - 0.94 (m, 1H), 0.74 (t, *J* = 7.2 Hz, 3H). ^{13}C NMR (100 MHz, $CDCl_3$) δ 142.6, 137.8, 136.0, 135.2, 134.1, 131.5, 131.3, 129.5, 129.4, 128.8, 128.1, 127.7, 127.0, 125.7, 125.4, 121.0, 120.9, 120.6, 98.1, 96.9, 89.5, 87.6, 61.3, 56.2, 43.3, 29.7, 26.8, 22.6, 22.5, 21.4, 13.9. HRMS (ESI) m/z: $[M+H]^+$ Calcd for $C_{36}H_{38}NOS$ 532.2674; Found 532.2654. $[\alpha]_D^{25}$ = 10.166 (c = 2.12, CH_2Cl_2).

(R)-2-methyl-N-((S)-3-phenyl-1-(8-(phenylethynyl)naphthalen-1-yl)hept-1-yn-3-yl)propane-2-sulfinamide (7b)

Isolation by column chromatography (petroleum ether/ ethyl acetate= 5/1 v/v), colorless oil, 32.0 mg, 62 % yield; ^{1}H NMR (400 MHz, $CDCl_3$) δ 8.05 (d, *J* = 6.8 Hz, 1H), 7.86 - 7.80 (m, 3H), 7.70 (d, *J* = 6.8 Hz, 2H), 7.50 - 7.42 (m, 2H), 7.31 - 7.23 (m, 5H), 7.19 - 7.14 (m, 1H), 7.12 - 7.06 (m, 2H), 3.49 (s, 1H), 2.20 - 2.12 (m, 1H), 1.87 - 1.78 (m, 1H), 1.50 - 1.41 (m, 1H), 1.18 (s, 9H), 1.14 - 1.05 (m, 2H), 1.02 - 0.94 (m, 1H), 0.75 (t, *J* = 7.2 Hz, 3H). ^{13}C NMR (100 MHz, $CDCl_3$) δ 142.5, 136.1, 135.2, 134.1, 131.6, 131.2, 129.6, 129.5, 128.2, 128.1, 127.8, 127.7, 127.0, 125.7, 125.4, 123.9, 120.8, 120.5, 98.0, 96.6, 90.2, 87.6, 61.3, 56.2, 43.3, 26.8, 22.7, 22.5, 13.9. HRMS (ESI) m/z: $[M+H]^+$ Calcd for $C_{35}H_{36}NOS$ 518.2518; Found 518.2532. $[\alpha]_D^{25}$ = 9.544 (c = 1.76, CH_2Cl_2).

(R)-N-((S)-1-(8-((4-ethylphenyl)ethynyl)naphthalen-1-yl)-3-phenylhept-1-yn-3-yl)-2-methylpropane-2-sulfinamide (7c)

Isolation by column chromatography (petroleum ether/ ethyl acetate= 5/1 v/v), colorless oil, 27.2 mg, 48 % yield; ^{1}H NMR (400 MHz, $CDCl_3$) δ 8.02 (d, *J* = 7.2 Hz, 1H), 7.84 - 7.79 (m, 3H), 7.69 (d, *J* = 7.6 Hz, 2H), 7.49 - 7.41 (m, 2H), 7.31 - 7.25 (m, 3H), 7.16 (d, *J* = 8.0 Hz, 2H), 6.92 (d, *J* = 7.6 Hz, 2H), 3.51 (s, 1H), 2.56 (q, *J* = 7.6 Hz,

2H), 2.19 - 2.11 (m, 1H), 1.87 - 1.78 (m, 1H), 1.46 - 1.41 (m, 1H), 1.20 - 1.15 (m, 12H), 1.12 - 1.06 (m, 2H), 0.99 - 0.95 (m, 1H), 0.74 (t, J = 7.2 Hz, 3H). ^{13}C NMR (100 MHz, $CDCl_3$) δ 144.2, 142.6, 136.0, 135.1, 134.1, 131.8, 131.6, 131.3, 129.5, 129.34.4, 128.1, 127.7, 127.6, 127.0, 125.7, 125.4, 121.1, 121.0, 120.6, 98.0, 96.9, 89.4, 87.6, 61.3, 56.2, 43.3, 29.7, 28.8, 26.8, 22.7, 22.5, 15.4, 13.9. HRMS (ESI) m/z: $[M+Na]^+$ Calcd for $C_{37}H_{39}NNaOS$ 568.2650; Found 568.2630. $[\alpha]_D^{25}$ = 15.071 (c = 1.04, CH_2Cl_2).

(R)-N-((S)-1-(8-((4-(tert-butyl)phenyl)ethynyl)naphthalen-1-yl)-3-phenylhept-1-yn-3-yl)-2-methylpropane-2-sulfinamide (7d)

Isolation by column chromatography (petroleum ether/ ethyl acetate= 5/1 v/v), colorless oil, 29.1 mg, 51 % yield; ^{1}H NMR (400 MHz, $CDCl_3$) δ 8.02 (d, J = 7.2 Hz, 1H), 7.84 - 7.79 (m, 3H), 7.68 (d, J = 7.2 Hz, 2H), 7.48 - 7.41 (m, 2H), 7.30 - 7.26 (m, 3H), 7.17 (d, J = 8.0 Hz, 2H), 7.09 (d, J = 8.0 Hz, 2H), 3.46 (s, 1H), 2.21 - 2.12 (m, 1H), 1.86 - 1.76 (m, 1H), 1.46 - 1.40 (m, 1H), 1.26 (s, 9H), 1.17 (s, 9H), 1.12 - 1.05 (m, 2H), 0.99 - 0.94 (m, 1H), 0.74 (t, J = 7.2 Hz, 3H). ^{13}C NMR (100 MHz, $CDCl_3$) δ 150.9, 142.6, 136.0, 135.1, 134.1, 131.3, 129.5, 129.4, 128.1, 127.7, 127.0, 125.6, 125.4, 125.0, 121.1, 120.9, 120.6, 98.0, 96.8, 89.4, 87.6, 61.2, 56.2, 43.3, 34.6, 31.1, 26.8, 22.7, 22.5, 13.9. HRMS (ESI) m/z: $[M+H]^+$ Calcd for $C_{39}H_{44}NOS$ 574.3144; Found 574.3116. $[\alpha]_D^{25}$ = 12.233 (c = 1.18, CH_2Cl_2).

(R)-N-((S)-1-(8-((4-methoxyphenyl)ethynyl)naphthalen-1-yl)-3-phenylhept-1-yn-3-yl)-2-methylpropane-2-sulfinamide (7e)

Isolation by column chromatography (petroleum ether/ ethyl acetate= 5/1 v/v), colorless oil, 24.6 mg, 45 % yield; ^{1}H NMR (400 MHz, $CDCl_3$) δ 8.02 (d, J = 7.2 Hz, 1H), 7.84 - 7.78 (m, 3H), 7.71 (d, J = 7.6 Hz, 2H), 7.49 - 7.40 (m, 2H), 7.32 - 7.25 (m, 3H), 7.16 (d, J = 8.0 Hz, 2H), 6.61 (d, J = 8.0 Hz, 2H), 3.75 (s, 3H), 3.51 (s, 1H), 2.20 - 2.12 (m, 1H), 1.88 - 1.79 (m, 1H), 1.48 - 1.42 (m, 1H), 1.19 (s, 9H), 1.15 - 1.06 (m, 2H), 0.99 - 0.94 (m, 1H), 0.76

(t, J = 7.2 Hz, 3H). ^{13}C NMR (100 MHz, $CDCl_3$) δ 159.2, 142.6, 136.0, 135.1, 134.1, 133.0, 131.3, 129.5, 129.3, 128.2, 127.7, 127.0, 125.6, 125.4, 121.1, 120.6, 116.2, 113.7, 98.0, 96.6, 88.7, 87.7, 61.3, 56.2, 55.2, 43.3, 29.7, 26.8, 22.7, 22.6, 13.9. HRMS (ESI) m/z: $[M+H]^+$ Calcd for $C_{36}H_{38}NO_2S$ 548.2623; Found 548.2591. $[\alpha]_D^{25}$ = 15.941 (c = 1.23, CH_2Cl_2).

(R)-N-((S)-1-(8-([1,1'-biphenyl]-4-ylethynyl)naphthalen-1-yl)-3-phenylhept-1-yn-3-yl)-2-methylpropane-2-sulfinamide (7f)

Isolation by column chromatography (petroleum ether/ ethyl acetate= 5/1 v/v), colorless oil, 34.4 mg, 58 % yield; ^{1}H NMR (400 MHz, $CDCl_3$) δ 8.05 (d, J = 6.8 Hz, 1H), 7.88 - 7.82 (m, 3H), 7.71 (d, J = 7.2 Hz, 2H), 7.52 (d, J = 8.0 Hz, 2H), 7.50 - 7.42 (m, 4H), 7.39 - 7.32 (m, 2H), 7.32 - 7.27 (m, 6H), 3.50 (s, 1H), 2.25 - 2.15 (m, 1H), 1.90 - 1.80 (m, 1H), 1.53 - 1.43 (m, 1H), 1.18 (s, 9H), 1.15 - 1.08 (m, 2H), 1.03 - 0.96 (m, 1H), 0.75 (t, J = 7.2 Hz, 3H). ^{13}C NMR (100 MHz, $CDCl_3$) δ 142.6, 140.5, 140.4, 136.1, 135.2, 134.1, 132.0, 131.3, 129.6, 129.6, 128.8, 128.3, 128.2, 127.8, 127.5, 127.0, 126.9, 126.7, 125.7, 125.4, 122.9, 120.8, 120.6, 98.0, 96.4, 90.9, 87.6, 61.2, 56.3, 43.3, 26.9, 22.6, 22.6, 13.9. HRMS (ESI) m/z: $[M+H]^+$ Calcd for $C_{41}H_{40}NOS$ 594.2831; Found 594.2823. $[\alpha]_D^{25}$ = 10.588 (c = 1.55, CH_2Cl_2).

(R)-N-((S)-1-(8-((4-chlorophenyl)ethynyl)naphthalen-1-yl)-3-phenylhept-1-yn-3-yl)-2-methylpropane-2-sulfinamide (7g)

Isolation by column chromatography (petroleum ether/ ethyl acetate= 5/1 v/v), colorless oil, 20.1 mg, 38 % yield; ^{1}H NMR (400 MHz, $CDCl_3$) δ 8.04 (dd, J = 7.2, 0.8 Hz, 1H), 7.86 - 7.81 (m, 3H), 7.71 - 7.66 (m, 2H), 7.50 - 7.42 (m, 2H), 7.33 - 7.27 (m, 3H), 7.13 - 7.09 (m, 2H), 7.05 - 7.01 (m, 2H), 3.43 (s, 1H), 2.22 - 2.14 (m, 1H), 1.88 - 1.79 (m, 1H), 1.49 - 1.40 (m, 1H), 1.19 (s, 9H), 1.16 - 1.10 (m, 2H), 1.02 - 0.94 (m, 1H), 0.76 (t, J = 7.2 Hz, 3H). ^{13}C NMR (100 MHz, $CDCl_3$) δ 142.4, 136.2, 135.3, 134.1, 133.7, 132.8, 131.2, 129.8, 129.6, 128.3, 128.2, 127.9, 126.9,

125.8, 125.4, 122.4, 120.4, 120.4, 97.9, 95.3, 91.1, 87.6, 61.2, 56.3, 43.3, 26.9, 22.6, 22.6, 13.9. HRMS (ESI) m/z: $[M+H]^+$ Calcd for $C_{35}H_{35}ClNOS$ 552.2128; Found 552.2127. $[\alpha]_D^{25}$ = 10.217 (c = 0.77, CH_2Cl_2).

(R)-N-((S)-1-(8-((4-bromophenyl)ethynyl)naphthalen-1-yl)-3-phenylhept-1-yn-3-yl)-2-methylpropane-2-sulfinamide (7h)

Isolation by column chromatography (petroleum ether/ ethyl acetate= 5/1 v/v), colorless oil, 20.8 mg, 35 % yield; ^{1}H NMR (400 MHz, $CDCl_3$) δ 8.04 (d, *J* = 7.2 Hz, 1H), 7.83 (d, *J* = 8.4 Hz, 3H), 7.68 (d, *J* = 7.6 Hz, 2H), 7.50 - 7.42 (m, 2H), 7.33 - 7.27 (m, 3H), 7.18 (d, *J* = 8.0 Hz, 2H), 7.04 (d, *J* = 8.0 Hz, 2H), 3.42 (s, 1H), 2.22 - 2.14 (m, 1H), 1.89 - 1.80 (m, 1H), 1.47 - 1.41 (m, 1H), 1.19 (s, 9H), 1.16 - 1.10 (m, 2H), 0.99 - 0.95 (m, 1H), 0.76 (t, *J* = 7.2 Hz, 3H). ^{13}C NMR (100 MHz, $CDCl_3$) δ 142.5, 136.2, 135.2, 134.1, 133.0, 131.2, 129.8, 129.6, 128.2, 127.9, 126.9, 125.8, 125.4, 122.8, 121.9, 120.4, 97.9, 95.4, 91.3, 87.6, 61.2, 56.3, 43.3, 29.7, 26.9, 22.6, 22.6, 13.9. HRMS (ESI) m/z: $[M+H]^+$ Calcd for $C_{35}H_{35}BrNOS$ 596.1623; Found 596.1606. $[\alpha]_D^{25}$ = 9.695 (c = 1.64, CH_2Cl_2).

(R)-N-((S)-1-(8-((4-cyanophenyl)ethynyl)naphthalen-1-yl)-3-phenylhept-1-yn-3-yl)-2-methylpropane-2-sulfinamide (7i)

Isolation by column chromatography (petroleum ether/ ethyl acetate= 5/1 v/v), colorless oil, 21.7 mg, 40 % yield; ^{1}H NMR (400 MHz, $CDCl_3$) δ 8.08 - 8.04 (m, 1H), 7.89 - 7.81 (m, 3H), 7.68 - 7.63 (m, 2H), 7.52 - 7.44 (m, 2H), 7.34 - 7.26 (m, 5H), 7.23 (d, *J* = 7.6 Hz, 2H), 3.34 (s, 1H), 2.24 - 2.15 (m, 1H), 1.88 - 1.78 (m, 1H), 1.51 - 1.42 (m, 1H), 1.19 (s, 9H), 1.17 - 1.09 (m, 2H), 1.00 - 0.92 (m, 1H), 0.76 (t, *J* = 7.6 Hz, 3H). ^{13}C NMR (100 MHz, $CDCl_3$) δ 142.3, 136.4, 135.4, 134.0, 132.0, 131.6, 131.1, 130.4, 129.7, 128.7, 128.3, 128.0, 126.8, 126.0, 125.4, 120.3, 119.7, 118.6, 110.7, 97.8, 94.8, 87.6, 61.1, 56.3, 43.5, 26.9, 22.6(9), 22.6(6), 13.9. HRMS (ESI) m/z: $[M+H]^+$ Calcd for $C_{36}H_{35}N_2OS$ 543.2470; Found 543.2469. $[\alpha]_D^{25}$ = 7.890 (c = 1.09, CH_2Cl_2).

Methyl 4-((8-((S)-3-(((R)-tert-butylsulfinyl)amino)-3-phenylhept-1-yn-1-yl)naphthalen-1-yl)ethynyl)benzoate (7j)

Isolation by column chromatography (petroleum ether/ ethyl acetate= 5/1 v/v), colorless oil, 33.1 mg, 58 % yield; ^{1}H NMR (400 MHz, $CDCl_3$) δ 8.06 (d, *J* = 6.4 Hz, 1H), 7.87 - 7.82 (m, 3H), 7.74 (d, *J* = 8.0 Hz, 2H), 7.69 - 7.65 (m, 2H), 7.48 (dd, *J* = 16.4, 8.0 Hz, 2H), 7.30 - 7.25 (m, 5H), 3.90 (s, 3H), 3.44 (s, 1H), 2.20 - 2.12 (m, 1H), 1.86 - 1.78 (m, 1H), 1.50 - 1.40 (m, 1H), 1.18 (s, 9H), 1.13 - 1.06 (m, 2H), 0.99 - 0.91 (m, 1H), 0.74 (t, *J* = 7.2 Hz, 3H). ^{13}C NMR (100 MHz, $CDCl_3$) δ 166.5, 142.3, 136.2, 135.4, 134.0, 131.4, 131.1(1), 130.1(5), 129.6, 129.2, 128.9, 128.6, 128.3, 127.9, 126.9, 125.9, 125.4, 120.4, 120.2, 98.0, 95.8, 93.4, 87.5, 61.2, 56.3, 52.1, 43.3, 26.9, 22.6, 22.5, 13.9. HRMS (ESI) m/z: [M+H] $^+$ Calcd for $C_{37}H_{38}NO_3S$ 576.2572; Found 576.2571. $[\alpha]_D^{25}$ = 8.855 (c = 1.66, CH_2Cl_2).

(R)-N-((S)-1-(8-((4-formylphenyl)ethynyl)naphthalen-1-yl)-3-phenylhept-1-yn-3-yl)-2-methylpropane-2-sulfinamide (7k)

Isolation by column chromatography (petroleum ether/ ethyl acetate= 5/1 v/v), colorless oil, 33.8 mg, 63% yield; ^{1}H NMR (400 MHz, $CDCl_3$) δ9.91 (s, 1H), 8.06 (d, J = 7.2 Hz, 1H), 7.88 - 7.82 (m, 3H), 7.66 (d, J = 7.6 Hz, 2H), 7.56 (d, J = 8.0 Hz, 2H), 7.52 - 7.43 (m, 2H), 7.33 (d, J = 8.0 Hz, 2H), 7.28 (d, J = 7.2 Hz, 3H), 3.41 (s, 1H), 2.22 - 2.13 (m, 1H), 1.86 - 1.78 (m, 1H), 1.51 - 1.41 (m, 1H), 1.18 (s, 9H), 1.14 - 1.07 (m, 2H), 0.99 - 0.90 (m, 1H), 0.74 (t, J = 7.2 Hz, 3H). ^{13}C NMR (100 MHz, $CDCl_3$) δ191.4, 142.3, 136.3, 135.4, 134.9, 134.0, 132.1, 131.1, 130.3, 130.2, 129.7, 129.2, 128.3, 127.9, 126.8, 125.9, 125.4, 120.3, 120.0, 97.9, 95.6, 94.5, 87.6, 61.2, 56.3, 43.4, 26.9, 22.6, 22.5, 13.9. HRMS (ESI) m/z: [M+H] $^+$ Calcd for $C_{36}H_{36}NO_2S$ 546.2467; Found 546.2459. $[\alpha]_D^{25}$ = 6.962 (c =1.69, CH_2Cl_2).

(R)-2-methyl-N-((S)-1-(8-((4-nitrophenyl)ethynyl)naphthalen-1-yl)-3-phenylhept-1-yn-3-yl)propane-2-sulfinamide (7l)

Isolation by column chromatography (petroleum ether/ ethyl acetate= 5/1 v/v), colorless oil, 25.7 mg, 46 % yield; ^{1}H NMR (400 MHz, $CDCl_3$) δ 8.07 (d, *J* = 7.2 Hz, 1H), 7.90 - 7.83 (m, 5H), 7.68 - 7.61 (m, 2H), 7.50 (dd, *J* = 17.6, 8.0 Hz, 2H), 7.30 - 7.25 (m, 5H), 3.35 (s, 1H), 2.24 - 2.14 (m, 1H), 1.89 - 1.79 (m, 1H), 1.51 - 1.41 (m, 1H), 1.20 (s, 9H), 1.17 - 1.07 (m, 2H), 1.01 - 0.92 (m, 1H), 0.76 (t, *J* = 7.2 Hz, 3H). ^{13}C NMR (100 MHz, $CDCl_3$) δ 146.4, 142.2, 136.4, 135.5, 134.0, 132.2, 131.5, 131.1, 130.7, 130.5, 129.7, 128.4, 128.0, 126.8, 126.0, 125.4, 123.7, 123.1, 120.3, 119.6, 97.9, 95.7, 94.6, 87.6, 61.1, 56.4, 43.6, 26.9, 22.6, 13.9. HRMS (ESI) m/z: $[M+H]^+$ Calcd for $C_{35}H_{35}N_2O_3S$ 563.2368; Found 563.2360. $[\alpha]_D^{25}$ = 3.306 (c = 1.21, CH_2Cl_2).

(R)-2-methyl-N-((S)-3-phenyl-1-(8-(m-tolylethynyl)naphthalen-1-yl)hept-1-yn-3-yl)propane-2-sulfinamide (7m)

Isolation by column chromatography (petroleum ether/ ethyl acetate= 5/1 v/v), colorless oil, 18.1 mg, 34 % yield; ^{1}H NMR (400 MHz, $CDCl_3$) δ 8.05 - 8.01 (m, 1H), 7.85 - 7.81 (m, 3H), 7.73 - 7.69 (m, 2H), 7.49 - 7.42 (m, 2H), 7.31 - 7.25 (m, 3H), 7.10 - 7.04 (m, 2H), 7.03 - 6.97 (m, 2H), 3.53 (s, 1H), 2.19 - 2.15 (m, 1H), 2.14 (s, 3H), 2.12 (d, *J* = 4.0 Hz, 1H), 1.86 - 1.77 (m, 1H), 1.47 - 1.40 (m, 1H), 1.17 (s, 9H), 1.13 - 1.06 (m, 2H), 1.00 - 0.94 (m, 1H), 0.74 (t, *J* = 3.2 Hz, 3H). ^{13}C NMR (100 MHz, $CDCl_3$) δ 142.5, 137.6, 136.0, 135.2, 134.1, 132.0, 131.2, 129.5, 128.8, 128.7, 128.2, 128.0, 127.7, 127.0, 125.7, 125.4, 123.7, 120.9, 120.5, 98.0, 96.8, 89.8, 87.6, 61.3, 56.2, 43.3, 29.7, 26.9, 22.6, 22.5, 21.1, 13.9. HRMS (ESI) m/z: $[M+H]^+$ Calcd for $C_{36}H_{38}NOS$ 532.2674; Found 532.2695. $[\alpha]_D^{25}$ = 15.844 (c = 0.64, CH_2Cl_2).

(R)-N-((S)-1-(8-((3-fluorophenyl)ethynyl)naphthalen-1-yl)-3-phenylhept-1-yn-3-yl)-2-methylpropane-2-sulfinamide (7n)

Isolation by column chromatography (petroleum ether/ ethyl acetate= 5/1 v/v), colorless oil, 29.9 mg, 56 % yield; ^{1}H NMR (400 MHz, $CDCl_3$) δ 8.05 (d, *J* = 7.2 Hz, 1H), 7.83 (d, *J* = 7.6 Hz, 3H), 7.67 (d, *J* = 6.4 Hz, 2H), 7.50 - 7.42 (m, 2H), 7.33 - 7.26 (m, 3H), 7.06 - 6.99 (m, 2H), 6.94 - 6.82 (m, 2H), 3.48 (s, 1H), 2.22 - 2.14 (m, 1H), 1.89 - 1.80 (m, 1H), 1.52 - 1.41 (m, 1H), 1.20 (s, 9H), 1.15 - 1.09 (m, 2H), 1.01 - 0.92 (m, 1H), 0.76 (t, *J* = 7.2 Hz, 3H). ^{13}C NMR (100 MHz, $CDCl_3$) δ 163.3, 160.9, 142.3, 136.2, 135.4, 134.0, 131.2, 129.9, 129.6, 129.5, 128.2, 127.9, 127.5(3), 127.5(8), 126.8, 125.8, 125.7, 125.4, 120.4, 120.3, 118.4, 118.2, 115.2, 115.0, 98.0, 95.2(1), 95.2(6), 91.1, 87.5, 61.2, 56.3, 43.3, 26.9, 22.6, 22.5, 13.9. HRMS (ESI) m/z: $[M+H]^+$ Calcd for $C_{35}H_{35}FNOS$ 536.2423; Found 536.2415. $[\alpha]_D^{25}$ = 8.067 (c = 1.50, CH_2Cl_2).

(R)-N-((S)-1-(8-((3-chlorophenyl)ethynyl)naphthalen-1-yl)-3-phenylhept-1-yn-3-yl)-2-methylpropane-2-sulfinamide (7o)

Isolation by column chromatography (petroleum ether/ ethyl acetate= 5/1 v/v), colorless oil, 19.8 mg, 36 % yield; ^{1}H NMR (400 MHz, $CDCl_3$) δ 8.07 - 8.03 (m, 1H), 7.86 - 7.81 (m, 3H), 7.70 - 7.65 (m, 2H), 7.50 - 7.43 (m, 2H), 7.30 - 7.26 (m, 3H), 7.24 (s, 1H), 7.16 - 7.09 (m, 2H), 7.02 - 6.98 (m, 1H), 3.49 (s, 1H), 2.22 - 2.13 (m, 1H), 1.89 - 1.80 (m, 1H), 1.50 - 1.41 (m, 1H), 1.21 (s, 9H), 1.17 - 1.10 (m, 2H), 0.99 - 0.91 (m, 1H), 0.76 (t, *J* = 7.2 Hz, 3H). ^{13}C NMR (100 MHz, $CDCl_3$) δ 142.3, 136.2, 135.4, 134.0, 133.9, 131.3, 131.1, 129.9, 129.8, 129.6, 129.2, 128.3, 128.0, 127.9, 126.8, 125.8, 125.6, 125.4, 120.4, 120.3, 98.0, 95.0, 91.4, 87.6, 61.2, 56.3, 43.3, 29.7, 26.9, 22.6, 22.5, 13.9. HRMS (ESI) m/z: $[M+H]^+$ Calcd for $C_{35}H_{35}ClNOS$ 552.2128; Found 552.2129. $[\alpha]_D^{25}$ = 7.400 (c = 1.0, CH_2Cl_2).

(R)-2-methyl-N-((S)-3-phenyl-1-(8-((3-(trifluoromethyl)phenyl)ethynyl)naphthalen-1-yl)hept-1-yn-3-yl)propane-2-sulfinamide (7p)

Isolation by column chromatography (petroleum ether/ ethyl acetate= 5/1 v/v), colorless oil, 31.4 mg, 54 % yield; ^{1}H NMR (400 MHz, $CDCl_3$) δ 8.07 (d, *J* = 7.2 Hz, 1H), 7.87 - 7.83 (m, 3H), 7.68 (d, *J* = 7.6 Hz, 2H), 7.57 (s, 1H), 7.52 - 7.45 (m, 2H), 7.39 (dd, *J* = 13.6, 7.6 Hz, 2H), 7.29 - 7.23 (m, 3H), 7.18 (t, *J* = 8.0 Hz, 1H), 3.49 (s, 1H), 2.20 - 2.13 (m, 1H), 1.89 - 1.81 (m, 1H), 1.51 - 1.42 (m, 1H), 1.20 (s, 9H), 1.12 - 1.06 (m, 2H), 1.01 - 0.93 (m, 1H), 0.73 (t, *J* = 7.2 Hz, 3H). ^{13}C NMR (100 MHz, $CDCl_3$) δ 142.2, 136.3, 135.6, 134.8, 134.0, 131.1, 130.8, 130.5, 130.1, 129.6, 128.5, 128.4, 128.2, 128.1(1), 128.1(7), 127.9, 127.0, 126.8, 125.9, 125.4, 125.0, 124.9, 124.3, 120.4, 120.1, 98.0, 94.9, 91.8, 87.6, 61.2, 56.3, 43.3, 26.8, 22.7, 22.6, 22.5, 13.8. HRMS (ESI) m/z: $[M+H]^+$ Calcd for $C_{36}H_{35}F_3NOS$ 586.2391; Found 586.2385. $[\alpha]_D^{25}$ = 11.361 (c = 1.58, CH_2Cl_2).

methyl 3-((8-((S)-3-(((R)-tert-butylsulfinyl)amino)-3-phenylhept-1-yn-1-yl)naphthalen-1-yl)ethynyl)benzoate (7q)

Isolation by column chromatography (petroleum ether/ ethyl acetate= 5/1 v/v), white solid, 34.4 mg, 60 % yield, mp 145.6-146.5 °C; ^{1}H NMR (400 MHz, $CDCl_3$) δ8.06 (d, *J* = 7.2 Hz, 1H), 7.99 (s, 1H), 7.87 - 7.81 (m, 4H), 7.65 (d, *J* = 7.6 Hz, 2H), 7.51 - 7.44 (m, 2H), 7.39 (d, *J* = 7.6 Hz, 1H), 7.25 - 7.13 (m, 4H), 3.89 (s, 3H), 3.55 (s, 1H), 2.18 - 2.09 (m, 1H), 1.87 - 1.79 (m, 1H), 1.48 - 1.39 (m, 1H), 1.21 (s, 9H), 1.12 - 1.04 (m, 2H), 1.00 - 0.92 (m, 1H), 0.74 (t, *J* = 7.2 Hz, 3H). ^{13}C NMR (100 MHz, $CDCl_3$) δ166.3, 142.2, 136.2, 135.7, 135.4, 134.0, 132.7, 131.1, 130.1, 129.9, 129.6, 128.8, 128.1(3), 128.1(1), 127.7, 126.8, 125.8, 125.4, 124.3, 120.4, 120.3, 98.0, 95.4, 91.1, 87.6, 61.3, 56.3, 52.1, 43.5, 26.8, 22.6, 22.5, 13.9. HRMS (ESI) m/z: $[M+H]^+$ Calcd for $C_{37}H_{38}NO_3S$ 576.2572; Found 576.2564. $[\alpha]_D^{25}$ = 7.160 (c = 0.25, CH_2Cl_2).

(R)-N-((S)-1-(8-((3-formylphenyl)ethynyl)naphthalen-1-yl)-3-phenylhept-1-yn-3-yl)-2-methylpropane-2-sulfinamide6 (7r)

Isolation by column chromatography (petroleum ether/ ethyl acetate= 5/1 v/v), colorless oil, 32.7 mg, 60 % yield; ^{1}H NMR (400 MHz, $CDCl_3$) δ 9.70 (s, 1H), 8.05 (d, *J* = 7.2 Hz, 1H), 7.87 - 7.83 (m, 3H), 7.66 (d, *J* = 7.6 Hz, 3H), 7.62 (s, 1H), 7.52 - 7.45 (m, 3H), 7.26 - 7.21 (m, 4H), 3.45 (s, 1H), 2.21 - 2.13 (m, 1H), 1.85 - 1.78 (m, 1H), 1.50 - 1.39 (m, 1H), 1.19 (s, 9H), 1.13 - 1.06 (m, 2H), 1.00 - 0.92 (m, 1H), 0.73 (t, *J* = 7.2 Hz, 3H). ^{13}C NMR (100 MHz, $CDCl_3$) δ 191.6, 142.3, 137.2, 136.3, 136.1, 135.4, 134.0, 133.6, 131.2, 130.1, 129.7, 128.7, 128.3, 127.9, 127.6, 126.9, 125.9, 125.4, 125.0, 120.4, 120.2, 97.8, 94.9, 91.7, 87.8, 61.2, 56.3, 43.6, 26.9, 22.6, 22.5, 13.9. HRMS (ESI) m/z: [M+Na] $^{+}$ Calcd for $C_{36}H_{35}NNaO_2S$ 568.2286; Found 568.2293. $[\alpha]_D^{25}$ = 10.508 (c = 1.64, CH_2Cl_2).

(R)-N-((S)-1-(8-((3-aminophenyl)ethynyl)naphthalen-1-yl)-3-phenylhept-1-yn-3-yl)-2-methylpropane-2-sulfinamide (7s)

Isolation by column chromatography (petroleum ether/ ethyl acetate= 5/1 v/v), colorless oil, 22.3 mg, 42 % yield; ^{1}H NMR (400 MHz, $CDCl_3$) δ 7.98 (dd, *J* = 7.2, 1.2 Hz, 1H), 7.84 - 7.79 (m, 3H), 7.77 - 7.73 (m, 2H), 7.48 - 7.43 (m, 2H), 7.35 - 7.26 (m, 3H), 6.92 - 6.88 (m, 1H), 6.71 (d, *J* = 7.6 Hz, 1H), 6.49 - 6.41 (m, 2H), 3.66 (s, 1H), 2.27 - 2.19 (m, 1H), 1.91 - 1.84 (m, 1H), 1.54 - 1.45 (m, 1H), 1.29 - 1.25 (m, 1H), 1.17 (s, 9H), 1.12 - 1.00 (m, 2H), 0.77 (t, *J* = 7.2 Hz, 3H). ^{13}C NMR (100 MHz, $CDCl_3$) δ 146.1, 142.8, 136.0, 135.2, 134.1, 132.1, 131.2, 129.6, 129.4, 129.0, 128.6, 128.4, 128.2, 127.6, 127.2, 125.6, 125.5, 124.5, 121.9, 121.0, 120.5, 117.8, 114.9, 97.8, 97.1, 89.5, 87.8, 61.5, 56.3, 43.6, 27.0, 22.6, 22.6, 13.9. HRMS (ESI) m/z: [M+Na] $^{+}$ Calcd for $C_{35}H_{36}N_2NaOS$ 555.2446; Found 555.2462. $[\alpha]_D^{25}$ = 2.523 (c = 1.07, CH_2Cl_2).

(R)-2-methyl-N-((S)-1-(8-((3-nitrophenyl)ethynyl)naphthalen-1-yl)-3-phenylhept-1-yn-3-yl)propane-2-sulfinamide (7t)

Isolation by column chromatography (petroleum ether/ ethyl acetate= 5/1 v/v), colorless oil, 30.3 mg, 54 % yield; ^{1}H NMR (400 MHz, $CDCl_3$) δ 8.06 (d, *J* = 7.2 Hz, 1H), 7.99 (s, 1H), 7.95 (d, *J* = 8.4 Hz, 1H), 7.89 - 7.83 (m, 3H),

7.62 (d, J = 7.6 Hz, 2H), 7.56 - 7.45 (m, 3H), 7.26 - 7.23 (m, 1H), 7.21 - 7.13 (m, 3H), 3.51 (s, 1H), 2.23 - 2.15 (m, 1H), 1.90 - 1.81 (m, 1H), 1.51 - 1.40 (m, 1H), 1.23 (s, 9H), 1.17 - 1.11 (m, 2H), 1.03 - 0.93 (m, 1H), 0.76 (t, J = 7.2 Hz, 3H). ^{13}C NMR (100 MHz, $CDCl_3$) δ147.7, 142.0, 137.3, 136.5, 135.5, 134.0, 131.1, 130.4, 129.7, 128.8, 128.1, 127.9, 126.6, 126.2, 126.0, 125.7, 125.4, 122.3, 120.3, 119.7, 97.9, 93.9, 92.7, 87.7, 61.2, 56.4, 43.8, 26.9, 22.6, 22.6, 13.9. HRMS (ESI) m/z: $[M+H]^+$ Calcd for $C_{35}H_{35}N_2O_3S$ 563.2368; Found 563.2360. $[\alpha]_D^{25}$ = 8.688 (c = 1.55, CH_2Cl_2).

(R)-N-((S)-1-(8-((3-hydroxyphenyl)ethynyl)naphthalen-1-yl)-3-phenylhept-1-yn-3-yl)-2-methylpropane-2-sulfinamide (7u)

Isolation by column chromatography (petroleum ether/ ethyl acetate= 5/1 v/v), colorless oil, 25.6 mg, 48 % yield; ^{1}H NMR (400 MHz, $CDCl_3$) δ 8.14 (s, 1H), 7.89 - 7.79 (m, 4H), 7.64 (d, J = 7.6 Hz, 2H), 7.48 - 7.44 (m, 2H), 7.16 (t, J = 7.6 Hz, 2H), 7.07 - 7.02 (m, 1H), 6.96 - 6.91 (m, 2H), 6.76 (d, J = 7.6 Hz, 1H), 6.63 (dd, J = 8.0, 2.0 Hz, 1H), 3.97 (s, 1H), 2.19 - 2.11 (m, 1H), 2.00 - 1.93 (m, 1H), 1.58 - 1.47 (m, 1H), 1.29 (s, 9H), 1.22 - 1.15 (m, 3H), 0.79 (t, J = 6.8 Hz, 3H). ^{13}C NMR (100 MHz, $CDCl_3$) δ 156.4, 142.2, 136.4, 134.8, 134.2, 131.5, 129.9, 129.3, 129.0, 128.2, 128.0, 127.5, 126.5, 125.7, 125.5, 124.2, 121.9, 121.0, 120.4, 119.0, 116.0, 97.7, 97.2, 89.3, 88.2, 62.8, 57.0, 45.8, 27.2, 22.7, 22.5, 13.9. HRMS (ESI) m/z: $[M+H]^+$ Calcd for $C_{35}H_{36}NO_2S$ 534.2467; Found 534.2463. $[\alpha]_D^{25}$ = 9.520 (c = 1.28, CH_2Cl_2).

(R)-2-methyl-N-((S)-3-phenyl-1-(8-(o-tolylethynyl)naphthalen-1-yl)hept-1-yn-3-yl)propane-2-sulfinamide (7v)

Isolation by column chromatography (petroleum ether/ ethyl acetate= 5/1 v/v), colorless oil, 25.0 mg, 48 % yield; ^{1}H NMR (400 MHz, $CDCl_3$) δ 8.07 - 8.03 (m, 1H), 7.85 - 7.81 (m, 3H), 7.69 - 7.65 (m, 2H), 7.50 - 7.42 (m, 2H), 7.30 - 7.26 (m, 3H), 7.10 (d, J = 6.8 Hz, 3H), 6.89 - 6.83 (m, 1H), 3.54 (s, 1H), 2.46 (s, 3H), 2.14 - 2.06 (m, 1H), 1.86 - 1.78 (m, 1H), 1.43 - 1.36 (m, 1H), 1.19 (s, 9H), 1.09 - 0.98 (m, 1H), 0.74 (t, J = 7.2 Hz, 3H). ^{13}C NMR (100

MHz, $CDCl_3$) δ 142.6, 140.4, 136.1, 135.1, 134.1, 131.6, 131.1, 129.5(7), 129.5(1), 129.3, 128.1, 127.9, 127.7, 126.9, 125.8, 125.4, 125.3, 123.7, 121.1, 120.6, 98.3, 95.7, 94.2, 87.6, 61.2, 56.3, 43.1, 26.7, 22.6, 22.5, 21.0, 13.9. HRMS (ESI) m/z: $[M+H]^+$ Calcd for $C_{36}H_{38}NOS$ 532.2674; Found 532.2668. $[\alpha]_D^{25}$ = 12.700 (c = 1.28, CH_2Cl_2).

(R)-N-((S)-1-(8-((2-chlorophenyl)ethynyl)naphthalen-1-yl)-3-phenylhept-1-yn-3-yl)-2-methylpropane-2-sulfinamide (7w)

Isolation by column chromatography (petroleum ether/ ethyl acetate= 5/1 v/v), colorless oil, 29.2 mg, 53 % yield; ^1H NMR (400 MHz, $CDCl_3$) δ 8.09 - 8.04 (m, 1H), 7.94 - 7.89 (m, 1H), 7.87 - 7.81 (m, 2H), 7.69 (d, J = 7.6 Hz, 2H), 7.51 - 7.46 (m, 2H), 7.31 - 7.26 (m, 3H), 7.24 - 7.20 (m, 1H), 7.18 - 7.15 (m, 1H), 7.12 - 7.07 (m, 1H), 6.93 - 6.88 (m, 1H), 3.65 (s, 1H), 2.17 - 2.08 (m, 1H), 1.93 - 1.85 (m, 1H), 1.48 - 1.40 (m, 1H), 1.22 (s, 9H), 1.12 - 1.06 (m, 2H), 0.92 - 0.82 (m, 1H), 0.74 (t, J = 7.2 Hz, 3H). ^{13}C NMR (100 MHz, $CDCl_3$) δ 142.4, 136.2, 136.1, 135.8, 134.0, 133.1, 131.0, 129.9, 129.5, 129.1, 128.8, 128.2, 127.7, 126.9, 126.1, 125.8, 125.4, 123.9, 120.5, 120.4, 98.2, 95.4, 93.5, 87.6, 61.4, 56.3, 43.3, 29.7, 26.8, 22.7, 22.5, 13.9. HRMS (ESI) m/z: $[M+H]^+$ Calcd for $C_{35}H_{35}ClNOS$ 552.2128; Found 552.2132. $[\alpha]_D^{25}$ = 5.445 (c = 1.64, $CHCl_3$).

(R)-2-methyl-N-((S)-3-phenyl-1-(8-((2-(trifluoromethyl)phenyl)ethynyl)naphthalen-1-yl)hept-1-yn-3-yl)propane-2-sulfinamide (7x)

Isolation by column chromatography (petroleum ether/ ethyl acetate= 5/1 v/v), colorless oil, 35.2 mg, 68 % yield; ^1H NMR (400 MHz, $CDCl_3$) δ8.08 (d, J = 7.2 Hz, 1H), 7.89 (d, J = 7.2 Hz, 1H), 7.87 - 7.82 (m, 2H), 7.72 (d, J = 8.0 Hz, 2H), 7.58 (d, J = 8.0 Hz, 1H), 7.51 - 7.45 (m, 2H), 7.28 - 7.20 (m, 5H), 7.12 - 7.07 (m, 1H), 3.68 (s, 1H), 2.20 - 2.11 (m, 1H), 1.98 - 1.89 (m, 1H), 1.51 - 1.40 (m, 1H), 1.22 (s, 9H), 1.13 - 1.03 (m, 3H), 0.71 (t, J = 6.8 Hz, 3H). ^{13}C NMR (100 MHz, $CDCl_3$) δ142.4, 136.4, 135.6, 134.0, 133.5, 131.3, 131.1, 130.9, 130.1, 129.6, 128.2, 127.8, 127.5, 127.0, 125.8, 125.6(4), 125.6(1), 125.5, 122.1, 120.4, 120.3, 98.1, 95.9, 92.7, 87.7, 61.6, 56.3, 43.4,

26.8, 22.6, 22.5, 13.7. HRMS (ESI) m/z: $[M+H]^+$ Calcd for $C_{36}H_{35}F_3NOS$ 586.2391; Found 586.2388. $[\alpha]_D^{25}$ = 9.234 (c = 1.11, CH_2Cl_2).

(R)-N-((S)-1-(8-((2-aminophenyl)ethynyl)naphthalen-1-yl)-3-phenylhept-1-yn-3-yl)-2-methylpropane-2-sulfinamide (7y)

Isolation by column chromatography (petroleum ether/ ethyl acetate= 5/1 v/v), colorless oil, 36.2 mg, 68 % yield; ^{1}H NMR (400 MHz, $CDCl_3$) δ7.99 (d, *J* = 7.2 Hz, 1H), 7.85 - 7.81 (m, 3H), 7.64 (d, *J* = 7.6 Hz, 2H), 7.50 - 7.43 (m, 2H), 7.26 - 7.17 (m, 3H), 6.99 - 6.93 (m, 2H), 6.51 (d, *J* = 8.4 Hz, 1H), 6.47 - 6.42 (m, 1H), 4.27 (s, 2H), 3.97 (s, 1H), 2.25 - 2.17 (m, 1H), 1.87 - 1.83 (m, 1H), 1.45 - 1.36 (m, 1H), 1.22 (s, 9H), 1.14 - 1.04 (m, 2H), 1.03 - 0.95 (m, 1H), 0.77 (t, *J* = 7.2 Hz, 3H). ^{13}C NMR (100 MHz, $CDCl_3$) δ147.8, 142.5, 136.1, 135.2, 134.1, 131.8, 131.2, 129.6, 129.2, 128.0, 127.6, 126.8, 125.7, 125.5, 120.8, 120.5, 117.7, 114.4, 109.1, 98.8, 94.9, 93.6, 87.4, 61.1, 56.3, 43.2, 26.8, 22.7, 22.5, 14.0. HRMS (ESI) m/z: $[M+H]^+$ Calcd for $C_{35}H_{35}N_2O_3S$ 563.2368; Found 563.2368. $[\alpha]_D^{25}$ = 12.210 (c = 1.81, CH_2Cl_2).

(R)-2-methyl-N-((S)-1-(8-((2-nitrophenyl)ethynyl)naphthalen-1-yl)-3-phenylhept-1-yn-3-yl)propane-2-sulfinamide (7z)

Isolation by column chromatography (petroleum ether/ ethyl acetate= 5/1 v/v), colorless oil, 42.1 mg, 75 % yield; ^{1}H NMR (400 MHz, $CDCl_3$) δ7.99 (d, *J* = 7.2 Hz, 1H), 7.85 - 7.81 (m, 3H), 7.64 (d, *J* = 7.6 Hz, 2H), 7.50 - 7.43 (m, 2H), 7.26 - 7.17 (m, 3H), 6.99 - 6.93 (m, 2H), 6.51 (d, *J* = 8.4 Hz, 1H), 6.47 - 6.42 (m, 1H), 4.27 (s, 2H), 3.97 (s, 1H), 2.25 - 2.17 (m, 1H), 1.87 - 1.83 (m, 1H), 1.45 - 1.36 (m, 1H), 1.22 (s, 9H), 1.14 - 1.04 (m, 2H), 1.03 - 0.95 (m, 1H), 0.77 (t, *J* = 7.2 Hz, 3H). ^{13}C NMR (100 MHz, $CDCl_3$) δ147.8, 142.5, 136.1, 135.2, 134.1, 131.8, 131.2, 129.6, 129.2, 128.0, 127.6, 126.8, 125.7, 125.5, 120.8, 120.5, 117.7, 114.4, 109.1, 98.8, 94.9, 93.6, 87.4, 61.1, 56.3,

43.2, 26.8, 22.7, 22.5, 14.0. HRMS (ESI) m/z: [M+H] $^+$ Calcd for $C_{35}H_{35}N_2O_3S$ 563.2368; Found 563.2368. $[\alpha]_D^{25}$ = 1.110 (c = 2.11, CH_2Cl_2).

(R)-2-methyl-N-((S)-1-(8-(naphthalen-1-ylethynyl)naphthalen-1-yl)-3-phenylhept-1-yn-3-yl)propane-2-sulfinamide (7aa)

Isolation by column chromatography (petroleum ether/ ethyl acetate= 5/1 v/v), colorless oil, 26.7 mg, 47 % yield; ^{1}H NMR (400 MHz, $CDCl_3$) δ 8.46 (d, *J* = 8.4 Hz, 1H), 8.08 (d, *J* = 7.2 Hz, 1H), 7.96 (d, *J* = 7.2 Hz, 1H), 7.88 - 7.84 (m, 2H), 7.78 (d, *J* = 7.6 Hz, 1H), 7.69 (d, *J* = 8.4 Hz, 1H), 7.60 - 7.55 (m, 2H), 7.53 - 7.46 (m, 4H), 7.40 (d, *J* = 7.2 Hz, 1H), 7.18 - 7.14 (m, 1H), 7.11 - 7.06 (m, 3H), 3.52 (s, 1H), 2.00 - 1.92 (m, 1H), 1.73 - 1.67 (m, 1H), 1.33 - 1.27 (m, 1H), 1.10 (s, 9H), 0.91 - 0.84 (m, 1H), 0.83 - 0.75 (m, 2H), 0.57 (t, *J* = 7.2 Hz, 3H). ^{13}C NMR (100 MHz, $CDCl_3$) δ 142.4, 136.2, 135.3, 134.1, 133.4, 133.2, 131.2, 130.1, 129.7, 129.5, 128.4, 128.1, 128.0, 127.5, 126.7, 126.7, 126.3, 125.8, 125.5, 125.0, 121.6, 120.9, 120.7, 98.5, 95.0, 94.9, 87.5, 61.2, 56.2, 43.0, 26.6, 22.5, 22.2, 13.7. HRMS (ESI) m/z: [M+H] $^+$ Calcd for $C_{39}H_{38}NOS$ 568.2674; Found 568.2664. $[\alpha]_D^{25}$ = 16.321 (c = 1.06, CH_2Cl_2).

(R)-N-((S)-1-(8-(anthracen-9-ylethynyl)naphthalen-1-yl)-3-phenylhept-1-yn-3-yl)-2-methylpropane-2-sulfinamide (7ab)

Isolation by column chromatography (petroleum ether/ ethyl acetate= 5/1 v/v), colorless oil, 40.0 mg, 65 % yield; ^{1}H NMR (400 MHz, $CDCl_3$) δ 8.72 (d, *J* = 8.4 Hz, 2H), 8.32 (s, 1H), 8.13 (dd, *J* = 7.2, 1.2 Hz, 1H), 8.04 (dd, *J* = 7.2, 1.2 Hz, 1H), 7.95 (d, *J* = 8.4 Hz, 2H), 7.91 - 7.87 (m, 2H), 7.54 - 7.44 (m, 6H), 7.30 - 7.27 (m, 2H), 6.79 - 6.75 (m, 1H), 6.73 - 6.69 (m, 2H), 3.47 (s, 1H), 1.60 - 1.46 (m, 2H), 1.37 - 1.21 (m, 2H), 1.15 (s, 9H), 0.94 - 0.88 (m, 1H), 0.80 - 0.72 (m, 1H), 0.39 (t, *J* = 7.2 Hz, 3H). ^{13}C NMR (100 MHz, $CDCl_3$) δ 142.0, 136.5, 135.2, 134.2, 132.5, 131.2, 131.1, 129.8, 129.5, 128.5, 127.5, 127.4, 126.9, 126.6, 126.1, 126.0, 125.6, 125.5, 121.0, 120.9, 118.4, 101.1,

99.5, 93.9, 87.2, 61.0, 56.3, 42.7, 26.0, 22.7, 21.9, 13.6. HRMS (ESI) m/z: $[M+H]^+$ Calcd for $C_{43}H_{40}NOS$ 618.2831; Found 618.2821. $[\alpha]_D^{25}$ = 10.380 (c = 1.97, CH_2Cl_2).

(R)-2-methyl-N-((S)-3-phenyl-1-(8-(thiophen-2-ylethynyl)naphthalen-1-yl)hept-1-yn-3-yl)propane-2-sulfinamide (7ac)

Isolation by column chromatography (petroleum ether/ ethyl acetate= 5/1 v/v), colorless oil, 26.2 mg, 51 % yield; ^{1}H NMR (400 MHz, $CDCl_3$) δ 8.04 (d, J = 6.8 Hz, 1H), 7.87 - 7.80 (m, 3H), 7.74 (d, J = 7.2 Hz, 2H), 7.50 - 7.43 (m, 2H), 7.34 - 7.27 (m, 3H), 7.12 (d, J = 4.8 Hz, 1H), 6.78 (d, J = 6.8 Hz, 2H), 3.65 (s, 1H), 2.25 - 2.17 (m, 1H), 1.96 - 1.88 (m, 1H), 1.51 - 1.43 (m, 1H), 1.21 (s, 9H), 1.17 - 1.10 (m, 2H), 1.04 (d, J = 11.6 Hz, 1H), 0.77 (t, J = 7.2 Hz, 3H). ^{13}C NMR (100 MHz, $CDCl_3$) δ 142.5, 136.0, 135.7, 134.0, 131.7, 131.2, 129.9, 129.5, 128.4, 128.2, 127.8, 127.1, 126.9, 126.8, 125.8, 125.4, 124.2, 120.5, 120.4, 98.3, 93.8, 89.8, 87.3, 61.4, 56.3, 43.3, 26.9, 22.7, 22.6, 13.9. HRMS (ESI) m/z: $[M+H]^+$ Calcd for $C_{33}H_{34}NOS_2$ 524.2082; Found 524.2076. $[\alpha]_D^{25}$ = 7.608 (c = 1.31, CH_2Cl_2).

(R)-2-methyl-N-((S)-3-phenyl-1-(8-(thiophen-3-ylethynyl)naphthalen-1-yl)hept-1-yn-3-yl)propane-2-sulfinamide (7ad)

Isolation by column chromatography (petroleum ether/ ethyl acetate= 5/1 v/v), colorless oil, 29.2 mg, 56 % yield; ^{1}H NMR (400 MHz, $CDCl_3$) δ 8.03 (d, J = 7.2 Hz, 1H), 7.82 (d, J = 8.8 Hz, 3H), 7.73 (d, J = 6.8 Hz, 2H), 7.49 - 7.41 (m, 2H), 7.35 - 7.28 (m, 3H), 7.08 - 7.03 (m, 1H), 7.01 - 6.95 (m, 1H), 6.88 (d, J = 4.8 Hz, 1H), 3.51 (s, 1H), 2.26 - 2.17 (m, 1H), 1.91 - 1.81 (m, 1H), 1.52 - 1.42 (m, 1H), 1.20 (s, 9H), 1.18 - 1.11 (m, 2H), 1.02 - 0.94 (m, 1H), 0.77 (t, J = 7.2 Hz, 3H). ^{13}C NMR (100 MHz, $CDCl_3$) δ 142.6, 136.0, 135.4, 134.1, 131.3, 130.0, 129.6, 129.5, 128.3, 128.2, 127.8, 127.1, 125.7, 125.4, 124.8, 123.1, 120.7, 120.5, 97.9, 91.8, 89.5, 87.5, 61.2, 56.3, 43.3, 26.9,

22.7, 22.6, 13.9. HRMS (ESI) m/z: $[M+H]^+$ Calcd for $C_{33}H_{34}NOS_2$ 524.2082; Found 524.2075. $[\alpha]_D^{25}$ = 9.542 (c = 1.31, CH_2Cl_2).

(R)-N-((S)-1-(8-(((R)-cyclohex-2-en-1-yl)ethynyl)naphthalen-1-yl)-3-phenylhept-1-yn-3-yl)-2-methylpropane-2-sulfinamide (7ae)

Isolation by column chromatography (petroleum ether/ ethyl acetate= 5/1 v/v), colorless oil, 27.1 mg, 52 % yield; ^{1}H NMR (400 MHz, $CDCl_3$) δ 8.01 (d, *J* = 7.2 Hz, 1H), 7.82 - 7.72 (m, 5H), 7.46 - 7.42 (m, 1H), 7.40 - 7.33 (m, 3H), 7.30 (d, *J* = 7.2 Hz, 1H), 5.87 (s, 1H), 3.74 (s, 1H), 2.40 - 2.32 (m, 1H), 2.12 - 2.06 (m, 1H), 2.05 - 1.96 (m, 2H), 1.92 - 1.84 (m, 2H), 1.62 - 1.54 (m, 1H), 1.46 - 1.39 (m, 4H), 1.34 - 1.28 (m, 2H), 1.27 (s, 9H), 1.22 - 1.16 (m, 1H), 0.86 (t, *J* = 7.2 Hz, 3H). ^{13}C NMR (100 MHz, $CDCl_3$) δ 142.6, 135.9, 135.1, 134.6, 134.1, 131.1, 129.5, 129.0, 128.3, 127.8, 127.1, 125.6, 125.4, 121.5, 121.4, 120.5, 98.7, 97.7, 87.7, 87.6, 61.6, 56.3, 43.5, 28.9, 27.0, 25.6, 22.8, 22.7, 22.2, 21.3, 14.0. HRMS (ESI) m/z: $[M+H]^+$ Calcd for $C_{35}H_{40}NOS$ 522.2831; Found 522.2822. $[\alpha]_D^{25}$ = 10.236 (c = 1.31, CH_2Cl_2).

(R)-2-methyl-N-((S)-3-phenyl-1-(8-(pyridin-2-ylethynyl)naphthalen-1-yl)hept-1-yn-3-yl)propane-2-sulfinamide (7af)

Isolation by column chromatography (petroleum ether/ ethyl acetate= 5/1 v/v), colorless oil, 24.3 mg, 47 % yield; ^{1}H NMR (400 MHz, $CDCl_3$) δ 8.34 (dd, *J* = 4.8, 0.8 Hz, 1H), 7.95 - 7.90 (m, 2H), 7.87 - 7.81 (m, 2H), 7.76 - 7.71 (m, 2H), 7.49 - 7.43 (m, 3H), 7.34 (d, *J* = 8.0 Hz, 1H), 7.25 - 7.20 (m, 2H), 7.16 - 7.11 (m, 1H), 7.08 - 7.03 (m, 1H), 5.99 (s, 1H), 2.37 - 2.29 (m, 1H), 2.02 - 1.94 (m, 1H), 1.53 - 1.42 (m, 1H), 1.26 (s, 9H), 1.19 - 1.11 (m, 2H), 1.03 - 0.94 (m, 1H), 0.75 (t, *J* = 7.2 Hz, 3H). ^{13}C NMR (100 MHz, $CDCl_3$) δ 149.2, 143.7, 142.0, 136.1, 135.8, 135.7, 134.0, 131.3, 130.4, 129.5, 127.8, 127.53, 127.5(8), 127.4, 125.7, 125.4, 122.3, 120.7, 119.9, 98.9, 95.0,

90.6, 86.5, 61.6, 56.3, 42.9, 27.0, 23.0, 22.6, 13.9. HRMS (ESI) m/z: [M+Na] $^+$ Calcd for $C_{34}H_{34}N_2NaOS$ 541.2290; Found 541.2296. $[\alpha]_D^{25}$ = 3.154 (c = 1.3, CH_2Cl_2).

(1S,2R,5S)-2-isopropyl-5-methylcyclohexyl 4-((8-((S)-3-(((R)-tert-butylsulfinyl)amino)-3-phenylhept-1-yn-1-yl)naphthalen-1-yl)ethynyl)benzoate (7ag)

Isolation by column chromatography (petroleum ether/ ethyl acetate= 5/1 v/v), colorless oil, 29.4 mg, 42 % yield; ^{1}H NMR (400 MHz, $CDCl_3$) δ 8.04 (d, *J* = 7.2 Hz, 1H), 7.84 (d, *J* = 6.4 Hz, 3H), 7.72 (d, *J* = 8.0 Hz, 2H), 7.64 (d, *J* = 8.0 Hz, 2H), 7.51 - 7.43 (m, 2H), 7.28 - 7.23 (m, 5H), 4.95 - 4.87 (m, 1H), 3.42 (s, 1H), 2.20 - 2.10 (m, 2H), 1.97 - 1.89 (m, 1H), 1.86 - 1.78 (m, 1H), 1.74 (d, *J* = 11.6 Hz, 2H), 1.59 - 1.52 (m, 2H), 1.47 - 1.39 (m, 1H), 1.18 (s, 9H), 1.15 - 1.05 (m, 4H), 1.00 (d, *J* = 15.2 Hz, 1H), 0.94 (d, *J* = 6.8 Hz, 6H), 0.91 - 0.85 (m, 1H), 0.82 (d, *J* = 6.8 Hz, 3H), 0.74 (t, *J* = 7.2 Hz, 3H). ^{13}C NMR (100 MHz, $CDCl_3$) δ 165.5, 142.3, 136.2, 135.2, 134.0, 131.4, 131.2, 130.0, 129.6, 129.1, 128.3, 128.2, 127.9, 126.8, 125.8, 125.4, 120.4, 120.3, 98.0, 95.8, 93.1, 87.5, 75.0, 61.1, 56.3, 47.3, 43.3, 41.0, 34.3, 31.5, 29.7, 26.8, 26.7, 23.8, 22.6, 22.5, 22.0, 20.7, 16.7, 13.9. HRMS (ESI) m/z: [M+H] $^+$ Calcd for $C_{46}H_{54}NO_3S$ 700.3824; Found 700.3831. $[\alpha]_D^{25}$ = 9.588 (c = 2.1, CH_2Cl_2).

(1S,2S,5R)-2-isopropyl-5-methylcyclohexyl 4-((8-((3S)-3-((tert-butylsulfinyl)amino)-3-phenylhept-1-yn-1-yl)naphthalen-1-yl)ethynyl)benzoate (7ah)

Isolation by column chromatography (petroleum ether/ ethyl acetate= 5/1 v/v), colorless oil, 39.2 mg, 56 % yield; ^{1}H NMR (400 MHz, $CDCl_3$) δ 8.04 (d, *J* = 7.2 Hz, 1H), 7.86 - 7.81 (m, 3H), 7.73 (d, *J* = 8.0 Hz, 2H), 7.66 (d, *J* = 7.2 Hz, 2H), 7.51 - 7.43 (m, 2H), 7.29 - 7.23 (m, 5H), 4.94 - 4.87 (m, 1H), 3.40 (s, 1H), 2.21 - 2.10 (m, 2H), 1.97 - 1.89 (m, 1H), 1.87 - 1.78 (m, 1H), 1.77 - 1.71 (m, 3H), 1.61 - 1.52 (m, 2H), 1.48 - 1.38 (m, 1H), 1.17 (s, 9H), 1.15 - 1.05 (m, 4H), 0.99 (d, *J* = 10.0 Hz, 1H), 0.94 (d, *J* = 6.8 Hz, 6H), 0.81 (d, *J* = 7.2 Hz, 3H), 0.73 (t, *J* = 7.2 Hz, 3H). ^{13}C NMR (100 MHz, $CDCl_3$) δ 165.5, 142.4, 136.2, 135.3, 134.0, 131.4, 131.2, 130.0, 129.6, 129.2, 128.4,

128.2, 127.9, 126.8, 125.8, 125.4, 120.4, 120.3, 98.1, 95.8, 93.1, 87.5, 75.0, 61.1, 56.3, 47.3, 43.3, 41.0, 34.3, 31.5, 26.8, 26.7, 23.8, 22.6, 22.5, 22.0, 20.7, 16.7, 13.9. HRMS (ESI) m/z: $[M+Na]^+$ Calcd for $C_{46}H_{53}NNaO_3$ 722.3644; Found 722.3658. $[\alpha]_D^{25}$ = 3.057 (c = 1.96, CH_2Cl_2).

(R)-2-methyl-N-((S)-3-phenyl-1-(8-((4-((((S)-2,5,7,8-tetramethyl-2-((4S,8S)-4,8,12-trimethyltridecyl)chroman-6-yl)oxy)methyl)phenyl)ethynyl)naphthalen-1-yl)hept-1-yn-3-yl)propane-2-sulfinamide (7ai)

Isolation by column chromatography (petroleum ether/ ethyl acetate= 5/1 v/v), colorless oil, 35.4 mg, 37 % yield; ^{1}H NMR (400 MHz, $CDCl_3$) δ 8.05 (d, *J* = 7.2 Hz, 1H), 7.88 - 7.82 (m, 3H), 7.71 (d, *J* = 7.6 Hz, 2H), 7.51 - 7.45 (m, 2H), 7.32 - 7.25 (m, 5H), 7.22 (d, *J* = 8.0 Hz, 2H), 4.63 (s, 2H), 3.52 (s, 1H), 2.64 - 2.59 (m, 2H), 2.21 (s, 3H), 2.16 (s, 3H), 2.13 (s, 3H), 1.90 - 1.78 (m, 3H), 1.60 - 1.53 (m, 3H), 1.52 - 1.45 (m, 2H), 1.44 - 1.38 (m, 3H), 1.36 - 1.30 (m, 4H), 1.29 - 1.22 (m, 9H), 1.20 (s, 9H), 1.17 - 1.07 (m, 8H), 0.88 (t, *J* = 6.8 Hz, 12H), 0.77 (t, *J* = 7.2 Hz, 3H). ^{13}C NMR (100 MHz, $CDCl_3$) δ 148.2, 148.0, 142.5, 137.9, 136.1, 135.2, 134.1, 131.6, 131.3, 129.6, 129.5, 128.2, 127.8, 127.8, 127.1, 127.0, 125.8, 125.7, 125.4, 123.2, 123.0, 120.8, 120.6, 117.7, 98.1, 96.6, 90.2, 87.6, 74.9, 74.2, 61.2, 56.3, 43.3, 40.1, 39.4, 37.5, 37.4, 37.3, 32.8, 32.7, 31.4, 28.0, 26.8, 24.8, 24.5, 23.9, 22.7, 22.6, 22.6, 21.1, 20.7, 19.8, 19.7, 13.9, 12.9, 12.0, 11.8. HRMS (ESI) m/z: $[M+H]^+$ Calcd for $C_{65}H_{86}NO_3S$ 960.6328; Found 960.6335. $[\alpha]_D^{25}$ = 7.679 (c = 1.12, CH_2Cl_2).

(3S,8R,9S,10S,13S,17S)-17-acetyl-10,13-dimethylhexadecahydro-1H-cyclopenta[a]phenanthren-3-yl 4-((8-((S)-3-(((R)-tert-butylsulfinyl)amino)-3-phenylhept-1-yn-1-yl)naphthalen-1-yl)ethynyl)benzoate (7aj)

Isolation by column chromatography (petroleum ether/ ethyl acetate= 5/1 v/v), colorless oil, 28.4 mg, 33 % yield; ^{1}H NMR (400 MHz, $CDCl_3$) δ 8.05 (d, *J* = 6.8 Hz, 1H), 7.91 - 7.78 (m, 3H), 7.74 (d, *J* = 8.4 Hz, 2H), 7.69 - 7.62 (m, 2H), 7.51 - 7.43 (m, 2H), 7.28 (d, *J* = 7.2 Hz, 3H), 7.24 (s, 1H), 5.43 (d, *J* = 4.0 Hz, 1H), 4.89 - 4.80 (m, 1H), 3.43 (s, 1H), 2.59 - 2.51 (m, 1H), 2.46 (d, *J* = 8.0 Hz, 2H), 2.13 (s, 3H), 2.10 - 1.93 (m, 4H), 1.83 - 1.64 (m, 5H),

1.61 (s, 6H), 1.59 - 1.40 (m, 5H), 1.30 - 1.21 (m, 6H), 1.19 (s, 9H), 1.15 - 1.09 (m, 2H), 1.08 (s, 3H), 0.90 - 0.82 (m, 1H), 0.73 (t, *J* = 7.2 Hz, 3H). ^{13}C NMR (100 MHz, $CDCl_3$) δ 209.5, 165.4, 142.3, 139.6, 136.2, 135.3, 134.0, 131.4, 131.1, 130.0, 129.6, 129.5, 129.2, 128.4, 128.3, 127.9, 126.9, 125.9, 125.4, 122.5, 120.4, 120.2, 98.0, 95.9, 93.2, 87.5, 74.6, 63.7, 61.2, 56.9, 56.3, 49.9, 44.0, 43.3, 38.8, 38.2, 37.1, 36.7, 31.8, 31.5, 27.9, 26.8, 24.5, 22.9, 22.6, 22.5, 21.1, 19.4, 13.9, 13.2. HRMS (ESI) m/z: $[M+H]^+$ Calcd for $C_{57}H_{68}NO_4S$ 862.4869; Found 862.4793. $[\alpha]_D^{25}$ = 7.596 (c = 1.04, CH_2Cl_2).

(R)-2-methyl-N-((S)-1-(8-(((8R,9S,13S,14S)-13-methyl-17-oxo-7,8,9,11,12,13,14,15,16,17-decahydro-6H-cyclopenta[a]phenanthren-3-yl)ethynyl)naphthalen-1-yl)-3-phenylhept-1-yn-3-yl)propane-2-sulfinamide (7ak)

Isolation by column chromatography (petroleum ether/ ethyl acetate= 5/1 v/v), colorless oil, 29.8 mg, 43 % yield; ^{1}H NMR (400 MHz, $CDCl_3$) δ 8.03 - 7.99 (m, 1H), 7.84 - 7.79 (m, 3H), 7.71 (d, *J* = 6.8 Hz, 2H), 7.48 - 7.41 (m, 2H), 7.31 - 7.26 (m, 3H), 7.06 - 7.01 (m, 2H), 6.98 (s, 1H), 3.55 (s, 1H), 2.71 - 2.60 (m, 2H), 2.55 - 2.48 (m, 1H), 2.34 (d, *J* = 8.8 Hz, 1H), 2.22 - 2.10 (m, 3H), 1.97 (d, *J* = 2.8 Hz, 1H), 1.87 - 1.80 (m, 1H), 1.64 - 1.58 (m, 7H), 1.50 - 1.45 (m, 3H), 1.17 (s, 9H), 1.12 - 1.06 (m, 2H), 0.92 (s, 3H), 0.74 (t, *J* = 7.2 Hz, 3H). ^{13}C NMR (100 MHz, $CDCl_3$) δ 142.5, 139.8, 136.2, 136.0, 135.2, 134.1, 131.9, 131.2, 129.5, 129.4, 129.1, 128.1, 127.6, 127.1, 125.7, 125.4, 125.1, 121.3, 121.0, 120.5, 97.9, 96.8, 89.5, 87.7, 61.3, 56.2, 50.5, 47.9, 44.4, 43.3, 38.1, 35.8, 31.6, 29.0, 26.9, 26.3, 25.6, 22.6, 22.5, 21.6, 13.9, 13.8. HRMS (ESI) m/z: $[M+H]^+$ Calcd for $C_{47}H_{52}NO_2S$ 694.3719; Found 694.3711. $[\alpha]_D^{25}$ = 9.704 (c = 1.18, CH_2Cl_2).

(R)-2-methyl-N-((S)-4-methyl-3-phenyl-1-(8-(phenylethynyl)naphthalen-1-yl)pent-1-yn-3-yl)propane-2-sulfinamide (7ba)

Isolation by column chromatography (petroleum ether/ ethyl acetate= 5/1 v/v), colorless oil, 24.6 mg, 49 % yield; ^{1}H NMR (400 MHz, $CDCl_3$) δ 8.20 - 8.16 (m, 1H), 7.88 - 7.85 (m, 1H), 7.84 - 7.80 (m, 2H), 7.73 - 7.70 (m, 2H),

7.50 - 7.42 (m, 2H), 7.36 - 7.33 (m, 2H), 7.26 - 7.20 (m, 3H), 7.19 - 7.11 (m, 3H), 3.74 (s, 1H), 2.16 - 2.08 (m, 1H), 1.15 (s, 9H), 1.01 (d, J = 6.8 Hz, 3H), 0.74 (d, J = 6.8 Hz, 3H). ^{13}C NMR (100 MHz, $CDCl_3$) δ 141.0, 136.6, 135.5, 134.1, 131.7, 131.0, 129.6, 129.4, 128.3, 128.0, 127.8, 127.6, 127.4, 125.9, 125.3, 124.2, 120.8, 120.6, 97.17, 96.8, 90.3, 89.0, 65.9, 56.5, 40.1, 22.8, 18.2, 18.1. HRMS (ESI) m/z: $[M+H]^+$ Calcd for $C_{34}H_{34}NOS$ 504.2361; Found 504.2359. $[\alpha]_D^{25}$ = 9.578 (c = 0.98, CH_2Cl_2).

(R)-2-methyl-N-((S)-5-methyl-3-phenyl-1-(8-(phenylethynyl)naphthalen-1-yl)hex-1-yn-3-yl)propane-2-sulfinamide (7bb)

Isolation by column chromatography (petroleum ether/ ethyl acetate= 5/1 v/v), colorless oil, 24.3 mg, 47 % yield; ^{1}H NMR (400 MHz, $CDCl_3$) δ 8.03 - 7.99 (m, 1H), 7.86 - 7.81 (m, 3H), 7.75 - 7.71 (m, 2H), 7.49 - 7.42 (m, 2H), 7.31 - 7.25 (m, 3H), 7.23 - 7.19 (m, 2H), 7.17 - 7.13 (m, 1H), 7.10 - 7.05 (m, 2H), 3.45 (s, 1H), 2.19 - 2.12 (m, 1H), 1.80 - 1.74 (m, 1H), 1.68 - 1.61 (m, 1H), 1.17 (s, 9H), 0.90 (d, J = 6.8 Hz, 3H), 0.49 (d, J = 6.4 Hz, 3H). ^{13}C NMR (100 MHz, $CDCl_3$) δ 142.5, 135.8, 135.3, 134.1, 131.6, 131.3, 129.6, 129.5, 128.2, 128.1, 127.8, 127.7, 127.1, 125.7, 125.4, 123.9, 120.8, 120.6, 98.1, 96.5, 90.3, 87.6, 61.0, 56.2, 52.1, 24.6, 24.1, 24.0, 22.6. HRMS (ESI) m/z: $[M+H]^+$ Calcd for $C_{35}H_{36}NOS$ 518.2518; Found 518.2519. $[a]_D^{25}$ = 5.422 (c = 1.21, CH_2Cl_2).

(S)-2-methyl-N-((R)-4-methyl-3-phenyl-1-(8-(p-tolylethynyl)naphthalen-1-yl)pent-1-yn-3-yl)propane-2-sulfinamide (7bc)

Isolation by column chromatography (petroleum ether/ ethyl acetate= 5/1 v/v), white solid, 28.9 mg, 55 % yield, mp 141.4-142.2 °C; ^{1}H NMR (400 MHz, $CDCl_3$) δ 8.16 (d, J = 7.2 Hz, 1H), 7.87 - 7.79 (m, 3H), 7.75 - 7.69 (m, 2H), 7.50 - 7.41 (m, 2H), 7.26 - 7.21 (m, 5H), 6.94 (d, J = 8.0 Hz, 2H), 3.73 (s, 1H), 2.28 (s, 3H), 2.17 - 2.10 (m, 1H), 1.15 (s, 9H), 1.01 (d, J = 6.8 Hz, 3H), 0.75 (d, J = 6.8 Hz, 3H). ^{13}C NMR (100 MHz, $CDCl_3$) δ 141.0, 137.9, 136.5, 135.5, 134.1, 131.6, 131.0, 129.5, 129.4, 128.7, 128.4, 127.6, 127.4, 125.9, 125.3, 121.1, 121.0, 120.6, 97.2, 97.1,

89.6, 89.0, 65.9, 56.5, 40.0, 22.8, 21.4, 18.2, 18.1. HRMS (ESI) m/z: [M+H] $^+$ Calcd for $C_{35}H_{36}NOS$ 518.2518; Found 518.2525. $[\alpha]_D^{25}$ = 9.238 (c = 1.44, CH_2Cl_2).

(R)-2-methyl-N-((R)-4-methyl-3-phenyl-1-(8-(p-tolylethynyl)naphthalen-1-yl)pent-1-yn-3-yl)propane-2-sulfinamide (7bc')

Isolation by column chromatography (petroleum ether/ ethyl acetate= 5/1 v/v), white solid, 30.5 mg, 59 % yield mp 139.8-140.7 °C; ^{1}H NMR (400 MHz, $CDCl_3$) δ 8.17 (d, *J* = 7.2 Hz, 1H), 7.83 (dd, *J* = 15.6, 7.6 Hz, 3H), 7.72 (d, *J* = 6.8 Hz, 2H), 7.50 – 7.41 (m, 2H), 7.22 (d, *J* = 6.8 Hz, 4H), 6.94 (d, *J* = 7.8 Hz, 2H), 3.74 (s, 1H), 2.28 (s, 3H), 1.15 (s, 9H), 1.00 (d, *J* = 6.8 Hz, 3H), 0.75 (d, *J* = 6.8 Hz, 3H). ^{13}C NMR (100 MHz, $CDCl_3$) δ 141.0, 137.9, 136.5, 135.5, 134.1, 131.6, 131.0, 129.5, 129.4, 128.7, 128.4, 127.6, 127.4, 125.9, 125.3, 121.1, 121.0, 120.6, 97.2, 97.1, 89.6, 89.0, 65.9, 56.5, 40.0, 22.8, 21.4, 18.2, 18.1. HRMS (ESI) m/z: [M+H] $^+$ Calcd for $C_{35}H_{36}NOS$ 518.2518; Found 518.2528. $[\alpha]_D^{25}$ = -10.233 (c = 1.62, CH_2Cl_2).

(S)-2-methyl-N-(3-phenyl-1-(8-(p-tolylethynyl)naphthalen-1-yl)hept-1-yn-3-yl)propane-2-sulfonamide (8a)

Isolation by column chromatography (petroleum ether/ ethyl acetate= 5/1 v/v), colorless oil, 44.2 mg, 81 % yield; ^{1}H NMR (400 MHz, $CDCl_3$) δ7.98 (dd, *J* = 7.2, 1.2 Hz, 1H), 7.86 – 7.81 (m, 3H), 7.71 (d, *J* = 6.8 Hz, 2H), 7.50 – 7.43 (m, 2H), 7.34 – 7.27 (m, 3H), 7.16 (d, *J* = 8.0 Hz, 2H), 6.93 (d, *J* = 7.6 Hz, 2H), 4.04 (s, 1H), 2.29 (s, 3H), 2.28 – 2.21 (m, 1H), 1.87 – 1.77 (m, 1H), 1.39 (s, 9H), 1.18 – 1.07 (m, 2H), 1.01 – 0.92 (m, 1H), 0.76 (t, *J* = 7.2 Hz, 3H). ^{13}C NMR (100 MHz, $CDCl_3$) 142.4, 138.1, 136.0, 135.0, 134.1, 131.5, 131.4, 129.7, 129.5, 129.0, 128.0, 127.6, 126.9, 125.7, 125.5, 120.9, 120.7, 120.5, 96.9, 96.7, 89.5, 87.4, 62.3, 60.3, 42.5, 27.0, 24.4, 22.4, 21.4, 13.9. HRMS (ESI) m/z: [M+Na] $^+$ Calcd for $C_{36}H_{37}NNaO_2S$ 570.2443; Found 570.2435. $[\alpha]_D^{25}$ = 10.238 (c = 2.12, CH_2Cl_2).

(S)-3-phenyl-1-(8-(p-tolylethynyl)naphthalen-1-yl)hept-1-yn-3-amine (8b)

Isolation by column chromatography (petroleum ether/ ethyl acetate= 5/1 v/v), white solid, 38.7 mg, 56 % yield, mp 88.7-89.9 °C; ^{1}H NMR (400 MHz, $CDCl_3$) δ 7.89 – 7.82 (m, 4H), 7.76 – 7.71 (m, 2H), 7.51 – 7.45 (m, 2H), 7.38 – 7.33 (m, 2H), 7.32 – 7.28 (m, 1H), 7.24 (d, J = 8.0 Hz, 2H), 7.03 (d, J = 7.6 Hz, 2H), 2.35 (s, 3H), 1.83 – 1.75 (m, 2H), 1.47 – 1.38 (m, 1H), 1.32 – 1.21 (m, 3H), 0.87 (t, J = 7.2 Hz, 3H). ^{13}C NMR (100 MHz, $CDCl_3$) 145.2, 138.3, 135.1, 134.8, 134.2, 131.7, 131.1, 129.5, 129.3, 129.2, 127.9, 127.0, 126.3, 125.5, 125.5, 121.2, 120.9, 120.7, 102.2, 96.6, 89.4, 84.3, 56.4, 45.8, 27.2, 22.8, 21.5, 14.0. HRMS (ESI) m/z: $[M+Na]^+$ Calcd for $C_{32}H_{29}NNa$ 450.2198; Found 450.2180. $[\alpha]_D^{25}$ = 10.444 (c = 1.84, CH_2Cl_2).

2 NMR spectra of Precursors **A3**, **A4**, **B3**, **C5** and **D5**

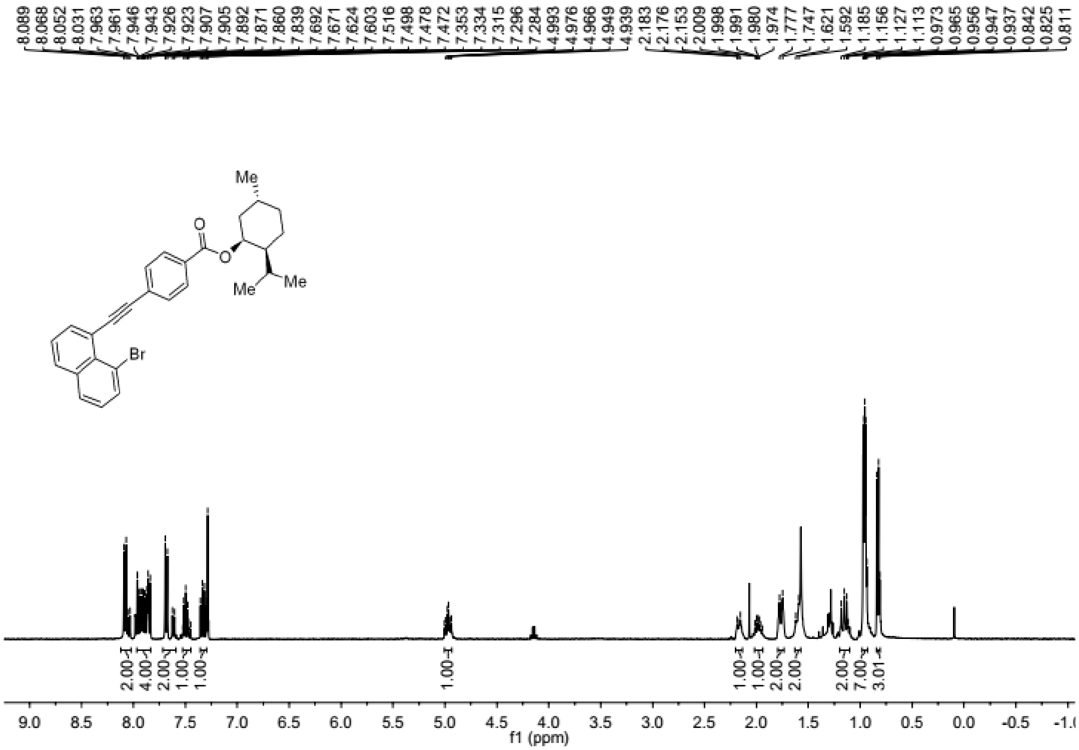

Figure S1. ^{1}H NMR Spectrum of Precursor A3 ($CDCl_3$, 400 MHz)

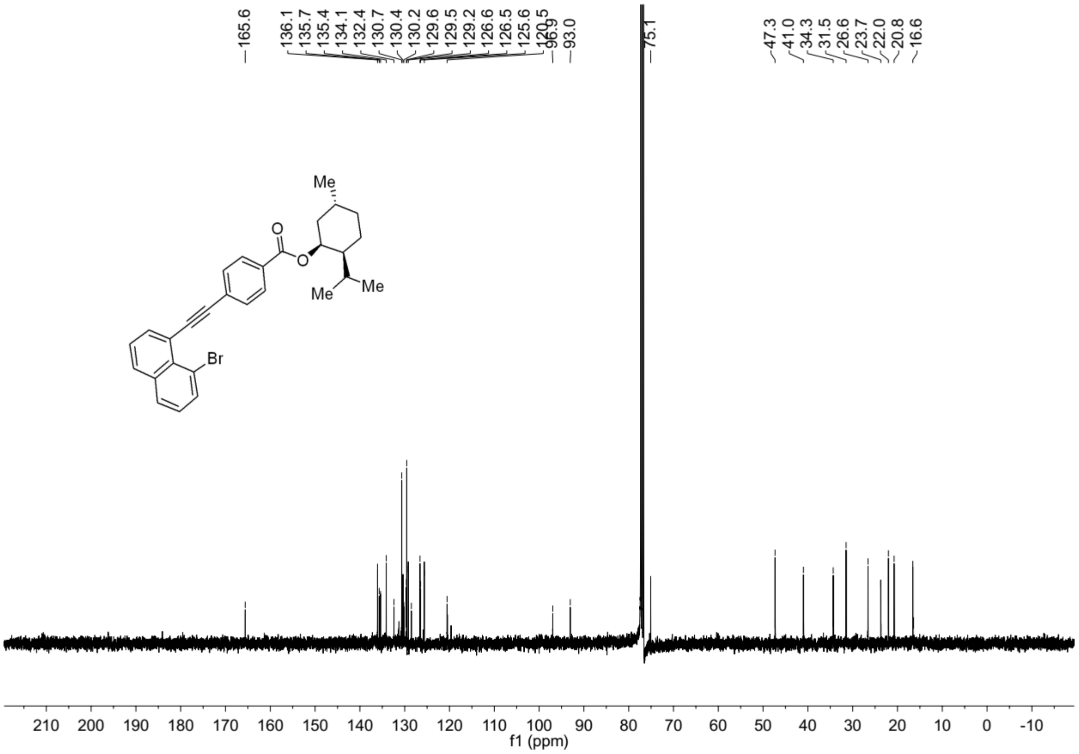

Figure S2. ^{13}C NMR Spectrum of Precursor A3 ($CDCl_3$, 100 MHz)

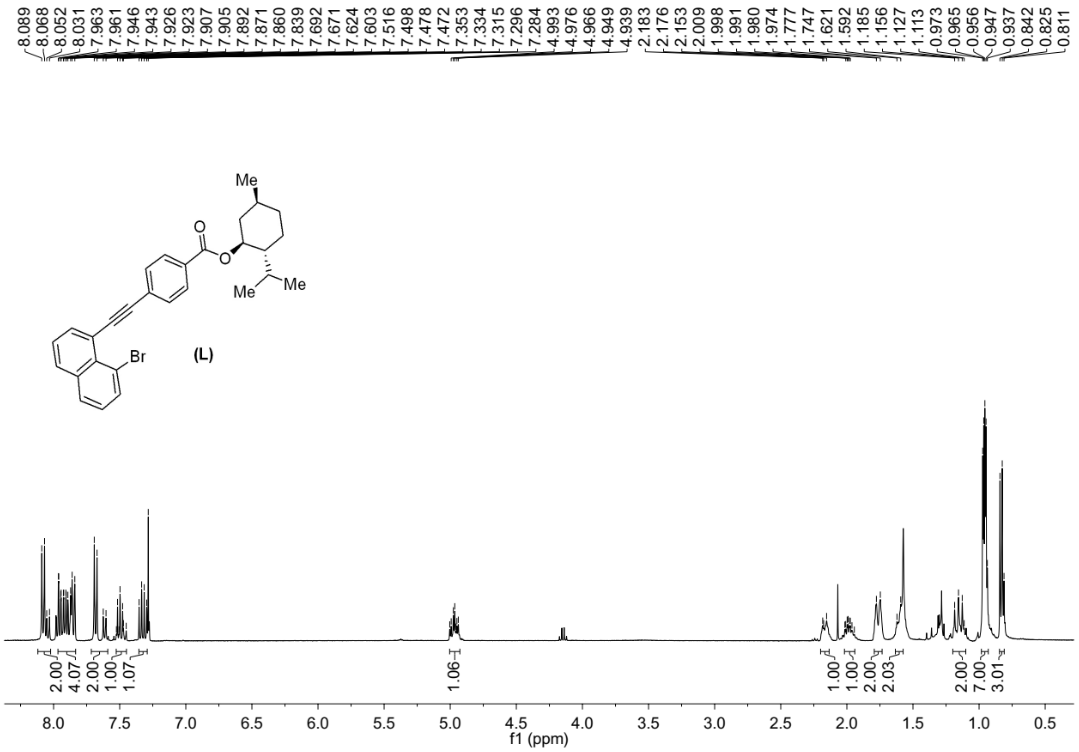

Figure S3. ^{1}H NMR Spectrum of Precursor A4 ($CDCl_3$, 400 MHz)

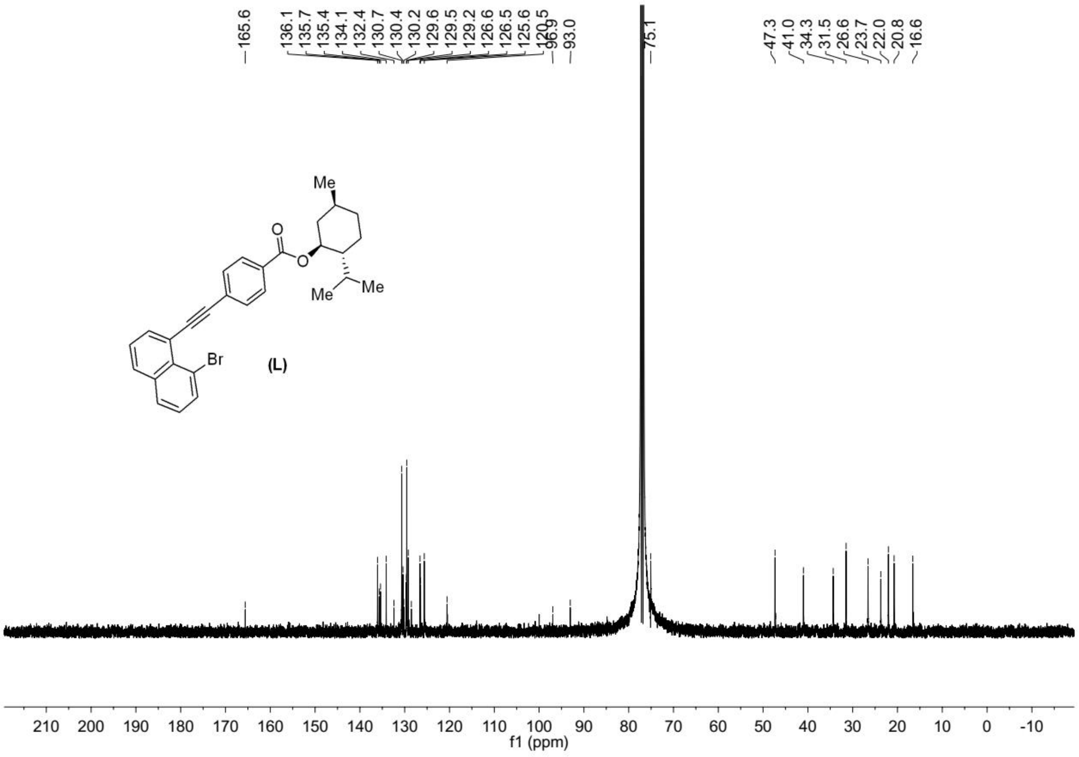

Figure S4. ^{13}C NMR Spectrum of Precursor A4 ($CDCl_3$, 100 MHz)

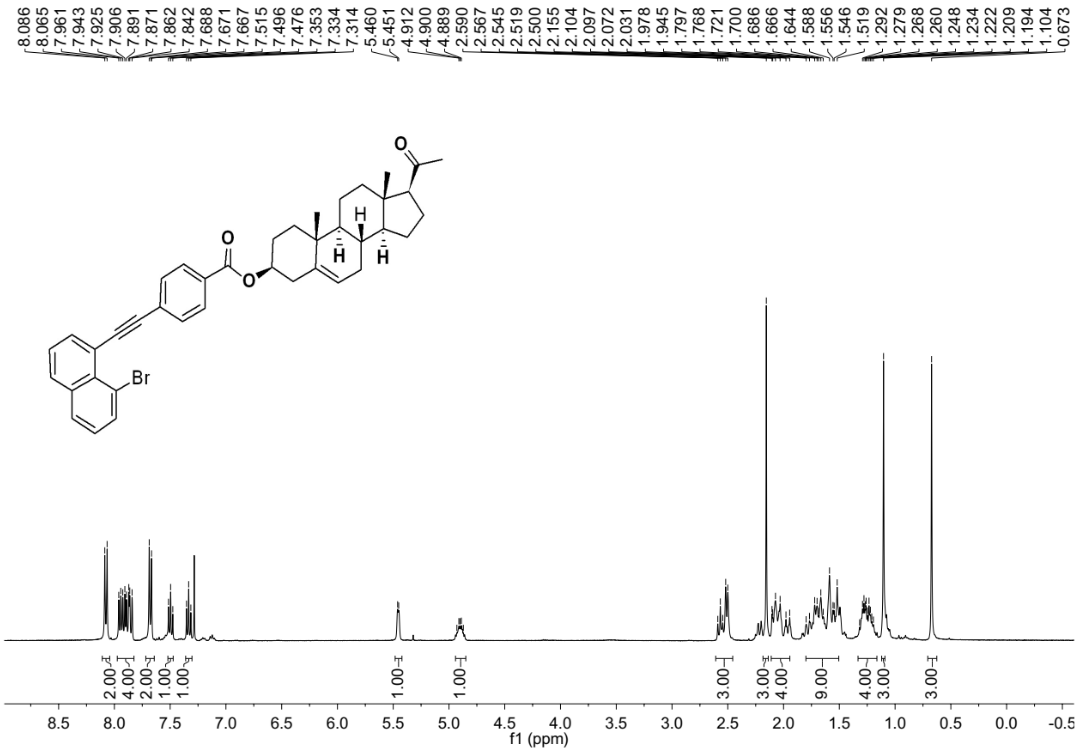

Figure S5. ^{1}H NMR Spectrum of Precursor B3 ($CDCl_3$, 400 MHz)

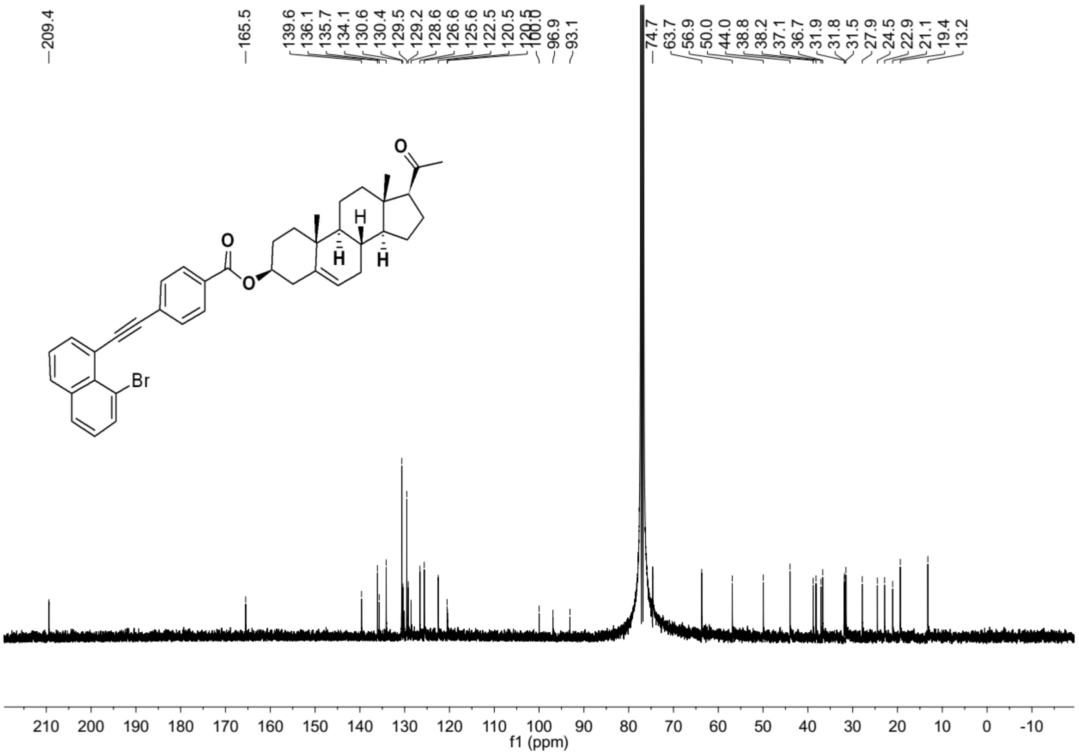

Figure S6. ^{13}C NMR Spectrum of Precursor B3 ($CDCl_3$, 100 MHz)

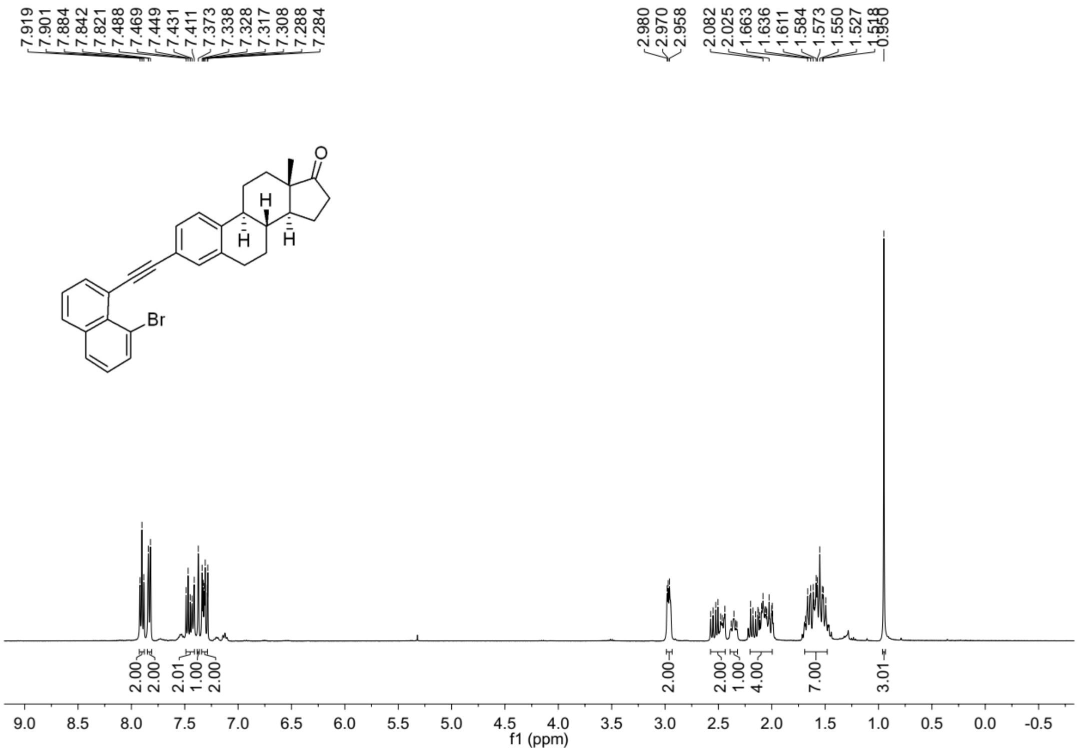

Figure S7. ^{1}**H NMR Spectrum of Precursor C5 (CDCl$_3$, 400 MHz)**

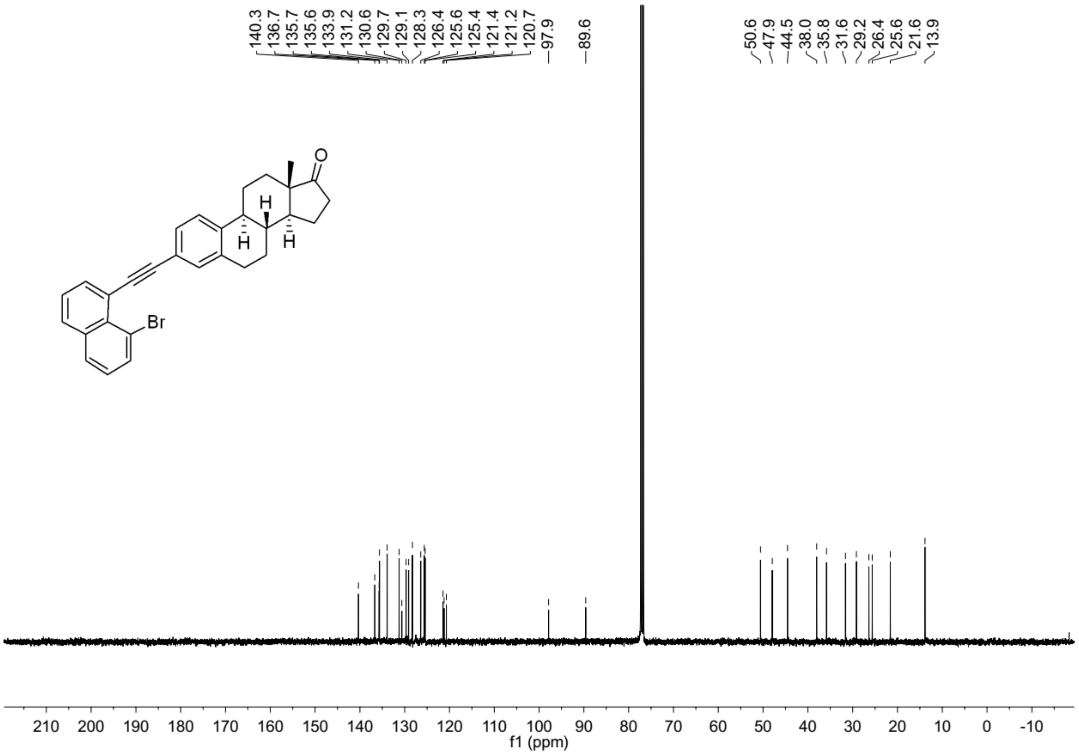

Figure S8. ^{13}**C NMR Spectrum of Precursor C5 (CDCl$_3$, 100 MHz)**

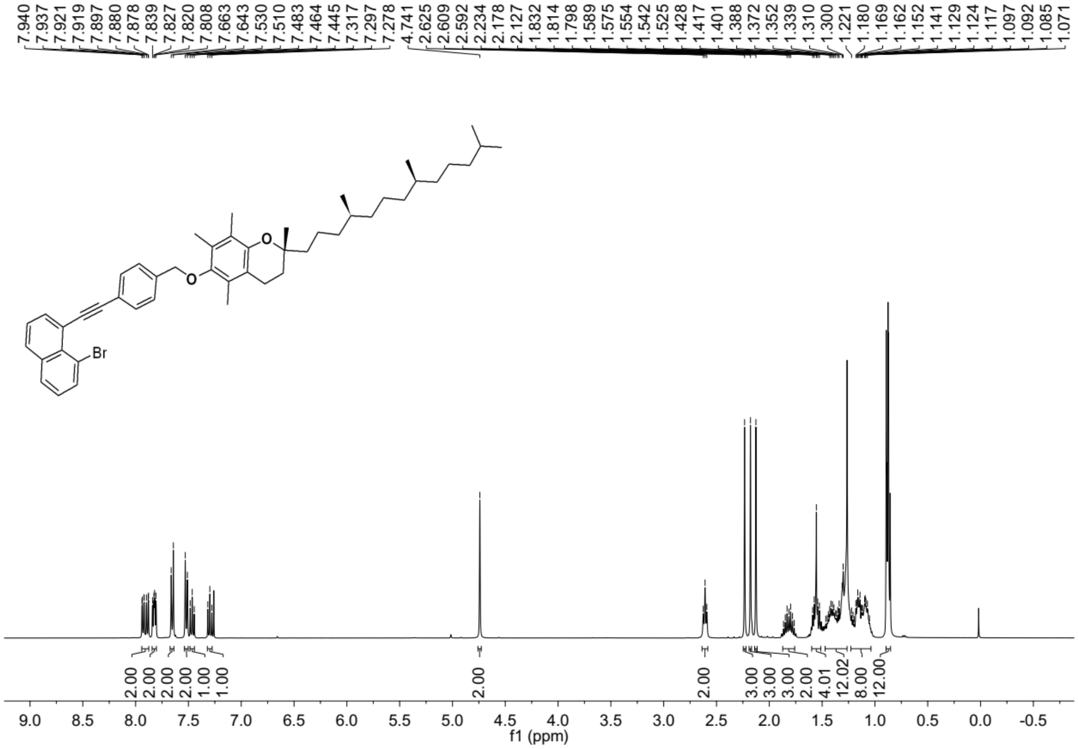

Figure S9. ^{1}H NMR Spectrum of Precursor D5 ($CDCl_3$, 400 MHz)

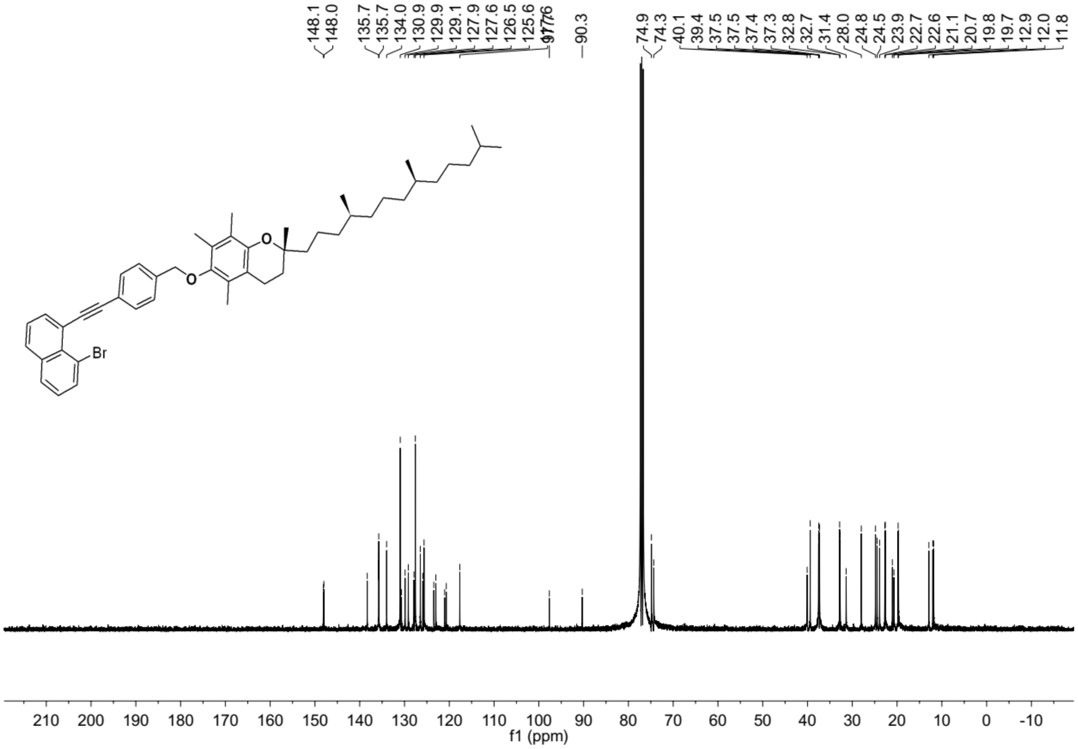

Figure S10. ^{13}C NMR Spectrum of Precursor D5 ($CDCl_3$, 100 MHz)

3 NMR spectra of Compounds **7a-8b**

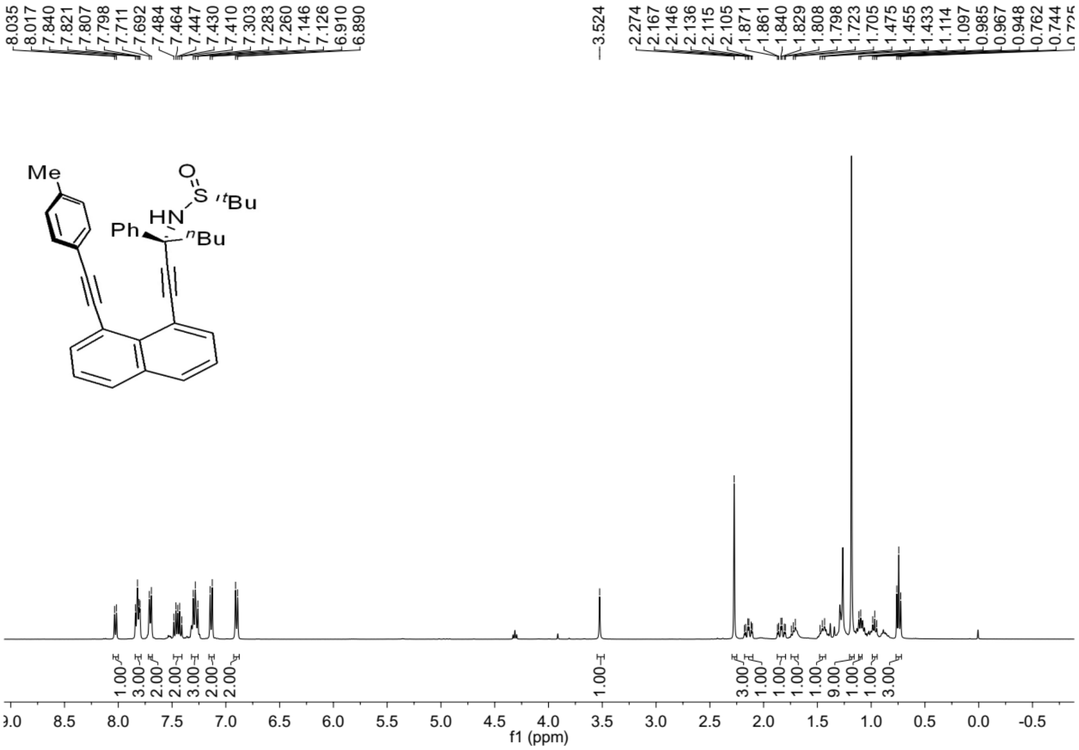

Figure S11. ^{1}H NMR Spectrum of Compound 7a ($CDCl_3$, 400 MHz)

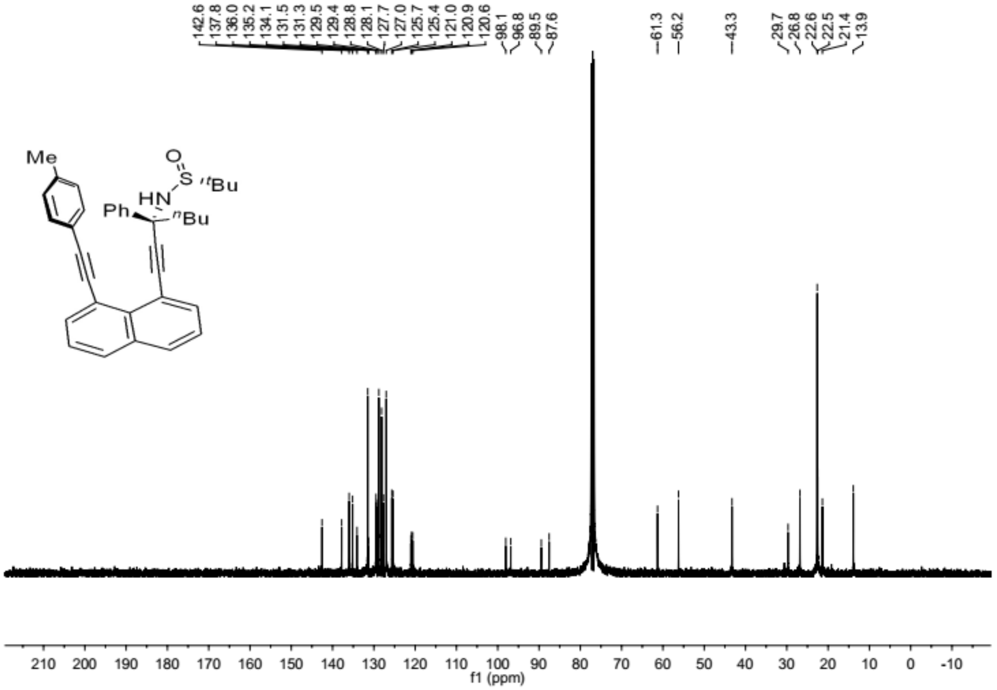

Figure S12. ^{13}C NMR Spectrum of Compound 7a ($CDCl_3$, 100 MHz)

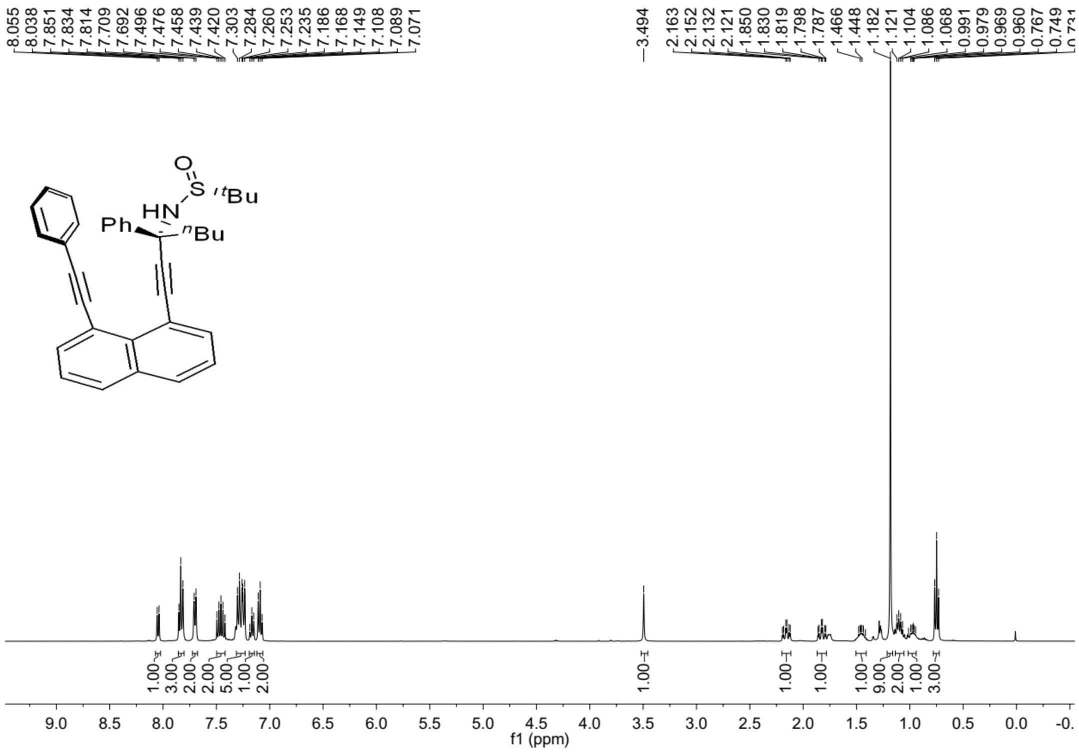

Figure S13. ^{1}**H NMR Spectrum of Compound 7b (CDCl$_3$, 400 MHz)**

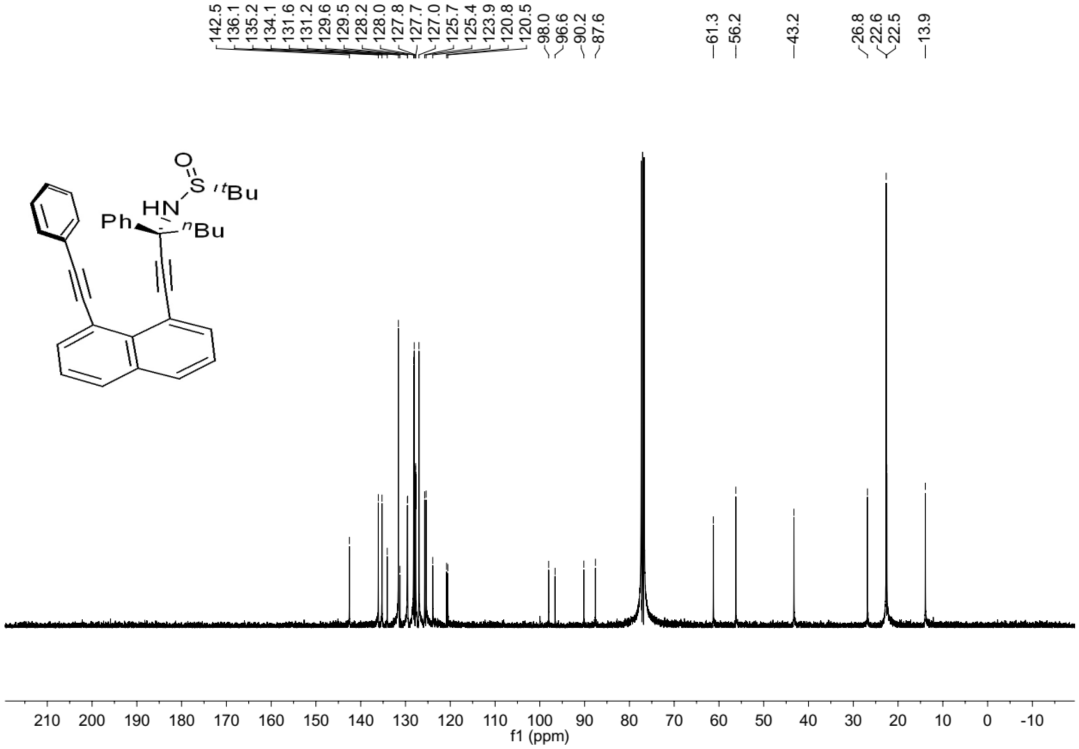

Figure S14. ^{13}**C NMR Spectrum of Compound 7b (CDCl$_3$, 100 MHz)**

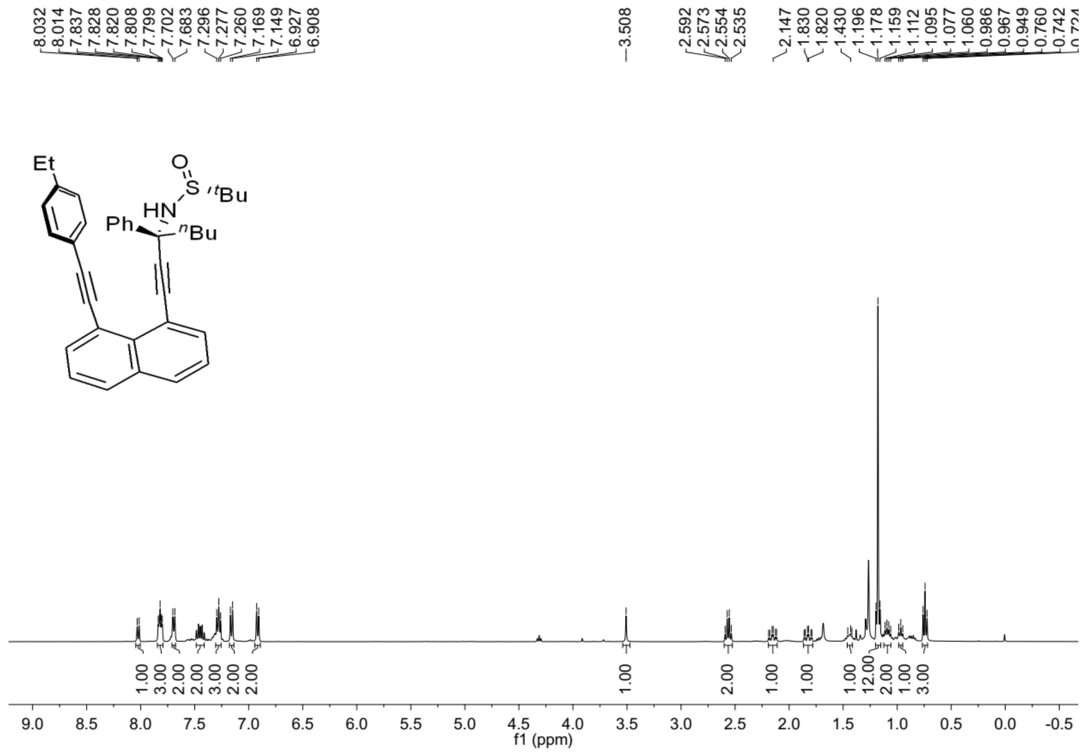

Figure S15. ^{1}H NMR Spectrum of Compound 7c ($CDCl_3$, 400 MHz)

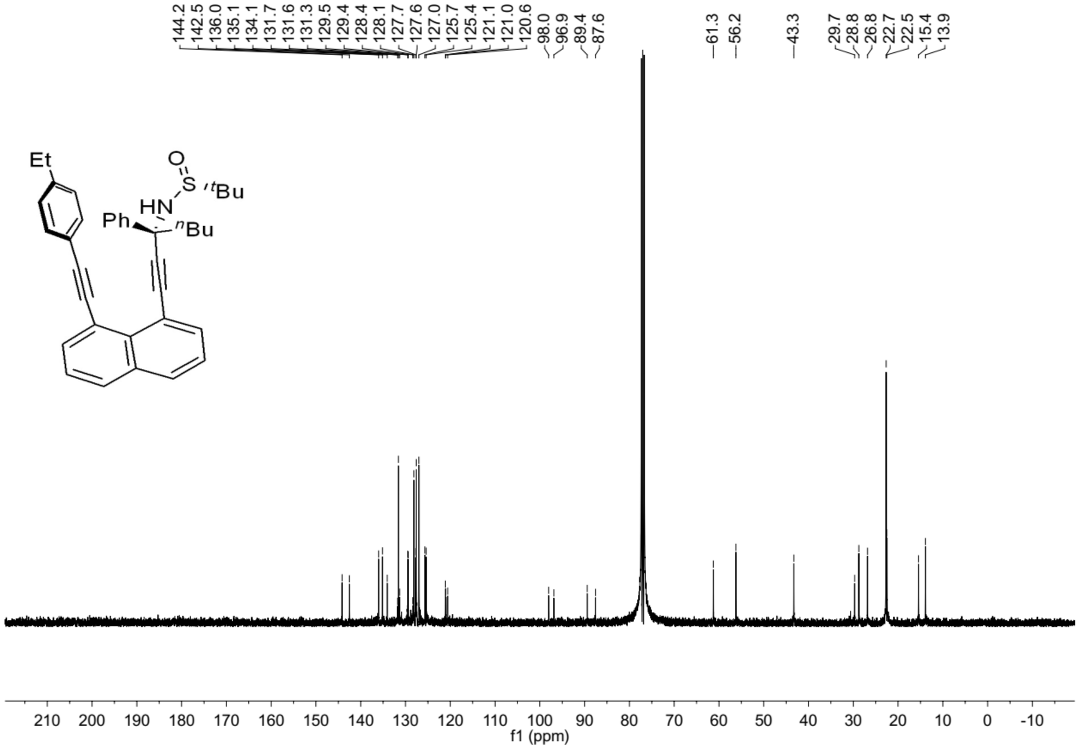

Figure S16. ^{13}C NMR Spectrum of Compound 7c ($CDCl_3$, 100 MHz)

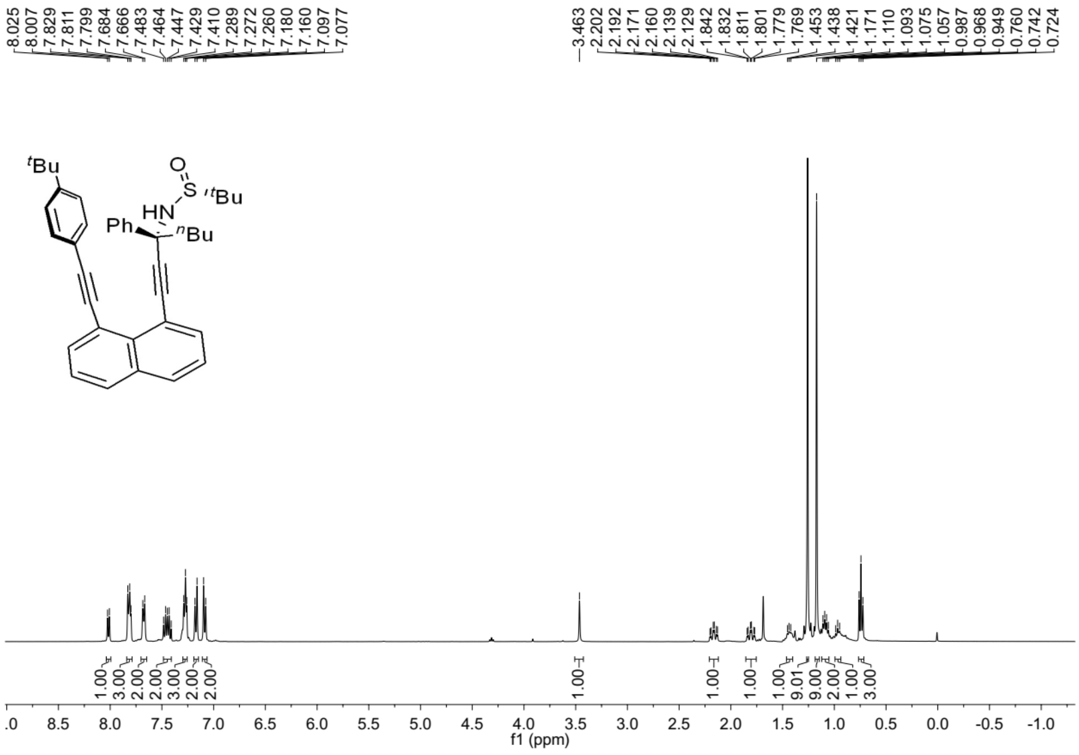

Figure S17. ^{1}H NMR Spectrum of Compound 7d ($CDCl_3$, 400 MHz)

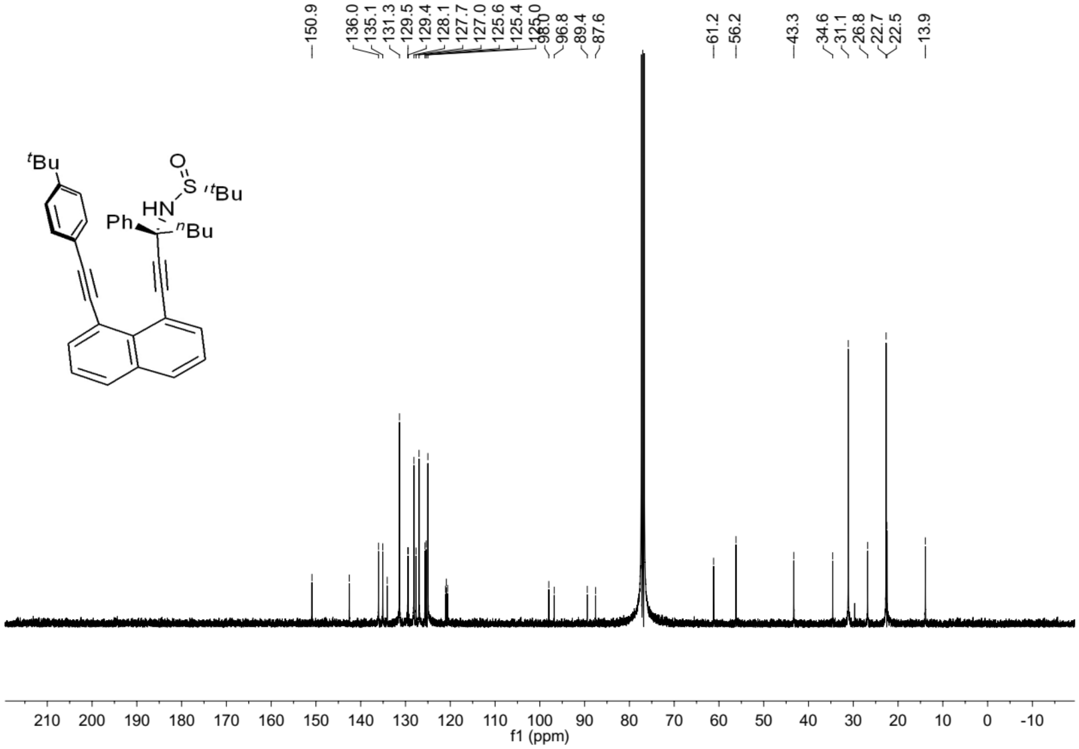

Figure S18. ^{13}C NMR Spectrum of Compound 7d ($CDCl_3$, 100 MHz)

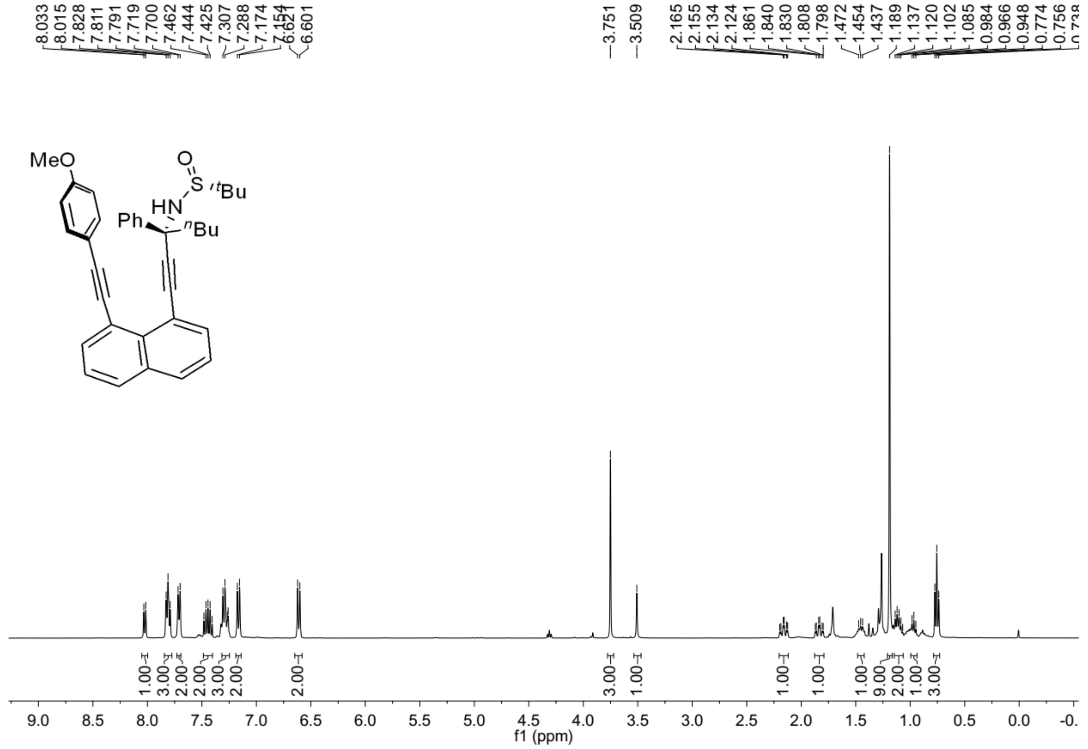

Figure S19. ^{1}H NMR Spectrum of Compound 7e ($CDCl_3$, 400 MHz)

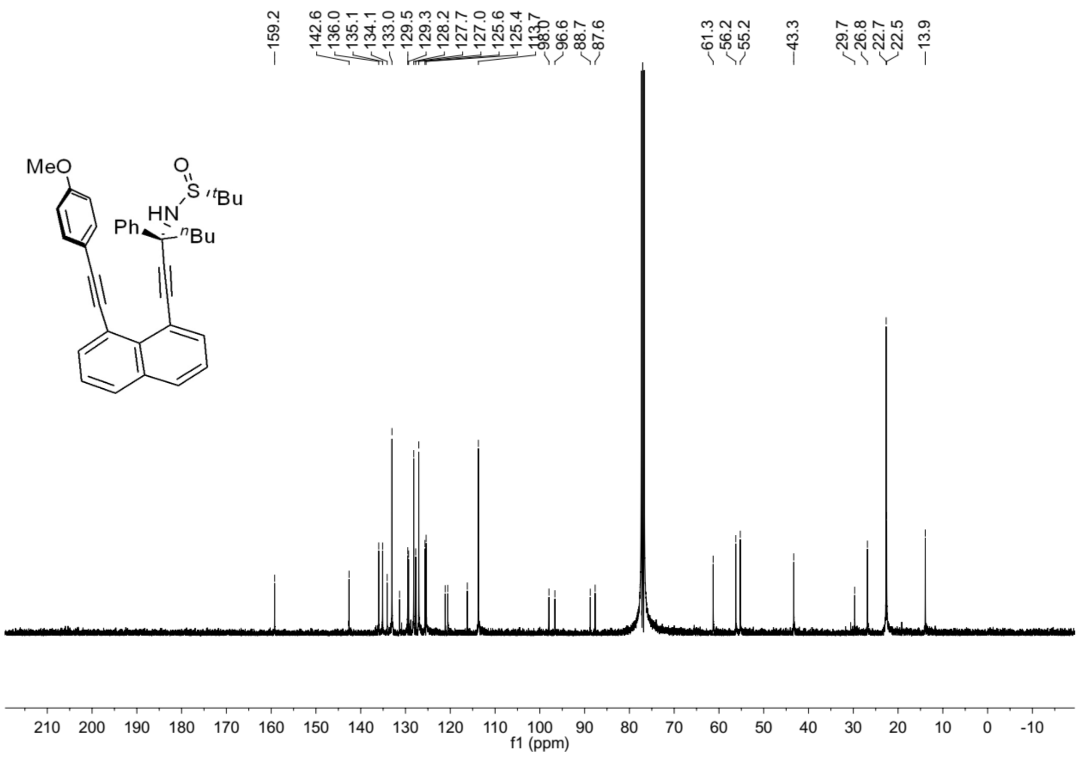

Figure S20. ^{13}C NMR Spectrum of Compound 7e ($CDCl_3$, 100 MHz)

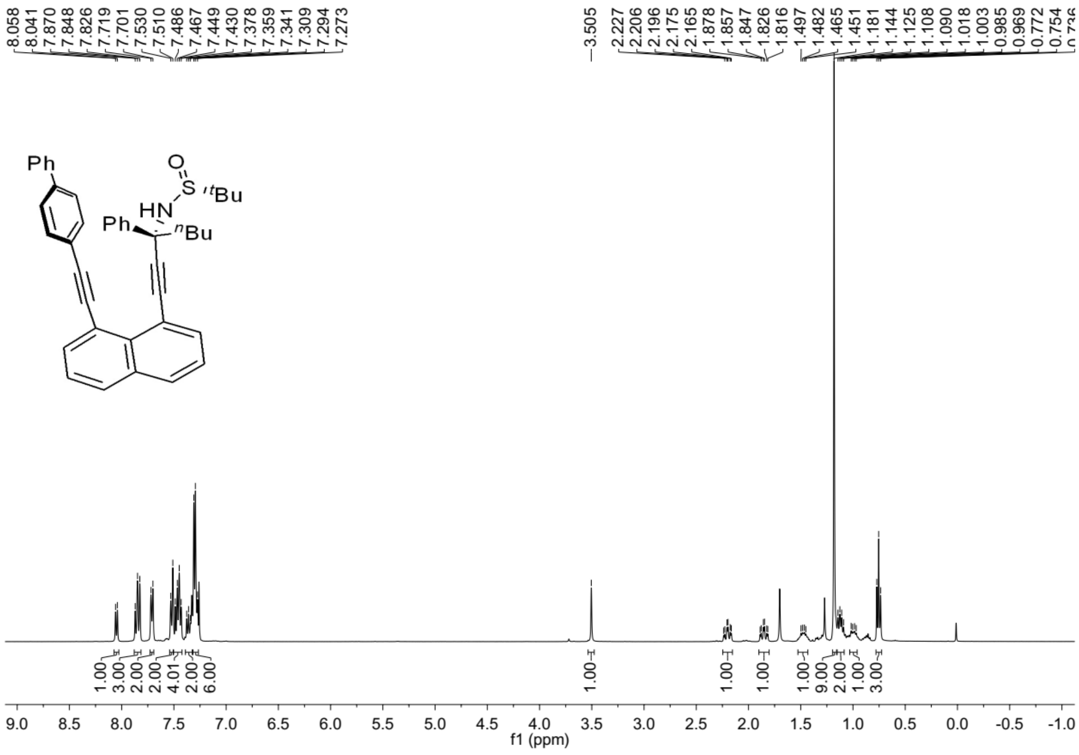

Figure S21. ^{1}H NMR Spectrum of Compound 7f ($CDCl_3$, 400 MHz)

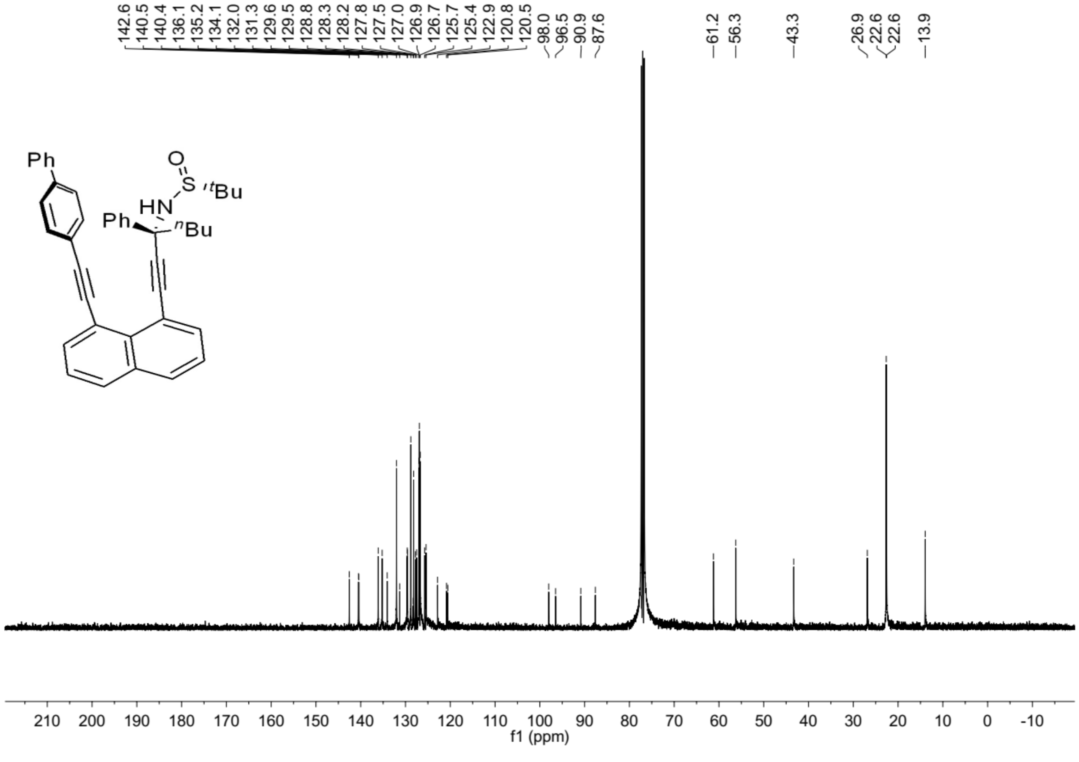

Figure S22. ^{13}C NMR Spectrum of Compound 7f ($CDCl_3$, 100 MHz)

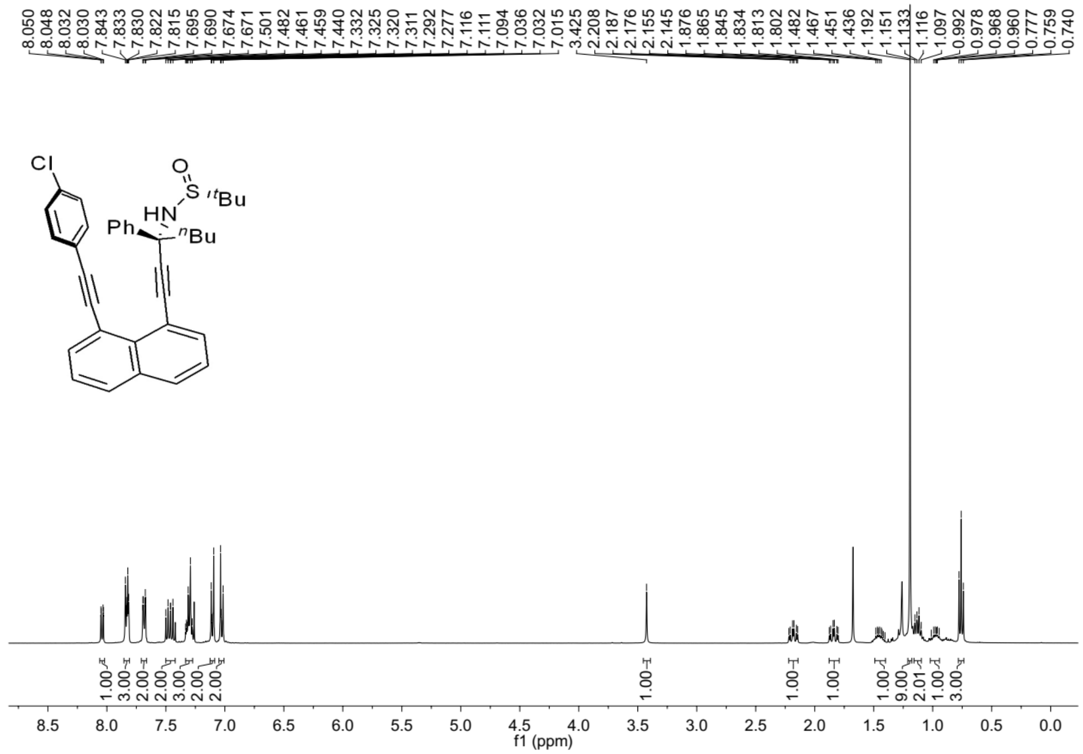

Figure S23. ^{1}**H NMR Spectrum of Compound 7g (CDCl$_3$, 400 MHz)**

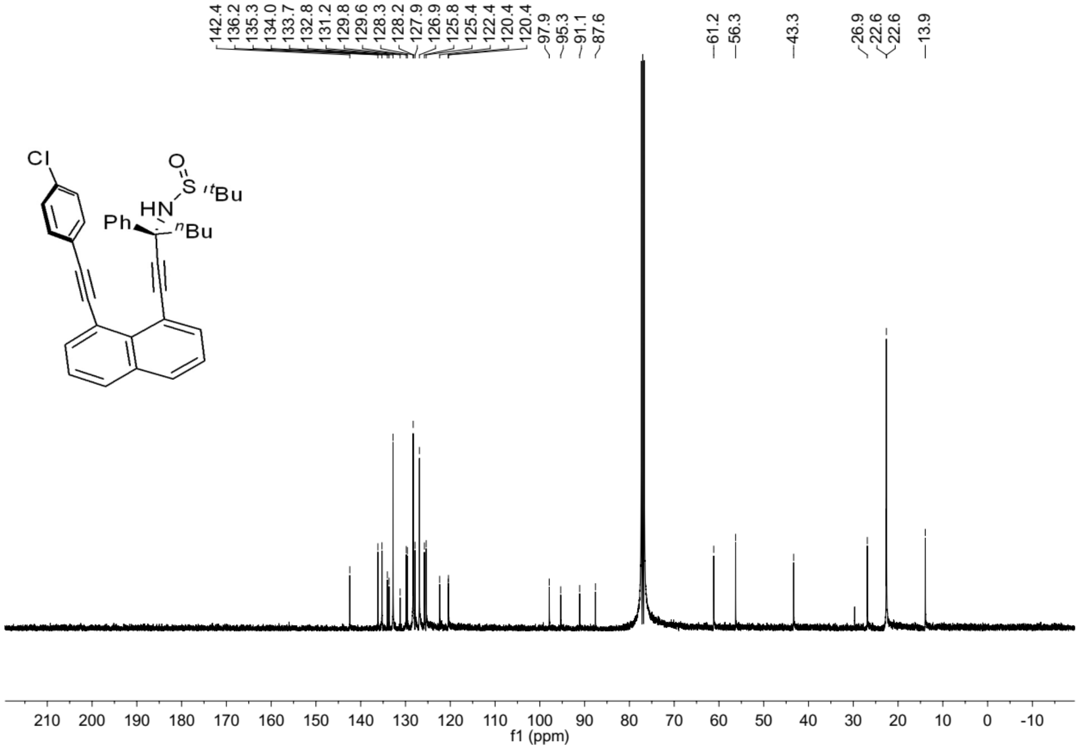

Figure S24. ^{13}**C NMR Spectrum of Compound 7g (CDCl$_3$, 100 MHz)**

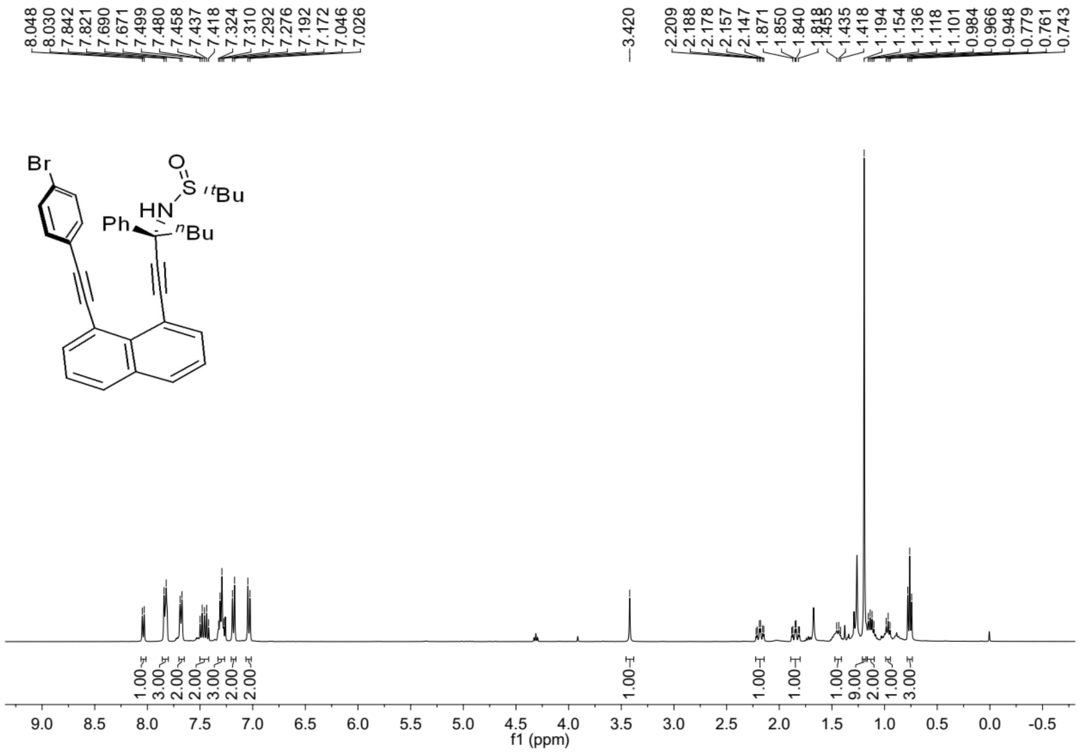

Figure S25. ^{1}H NMR Spectrum of Compound 7h ($CDCl_3$, 400 MHz)

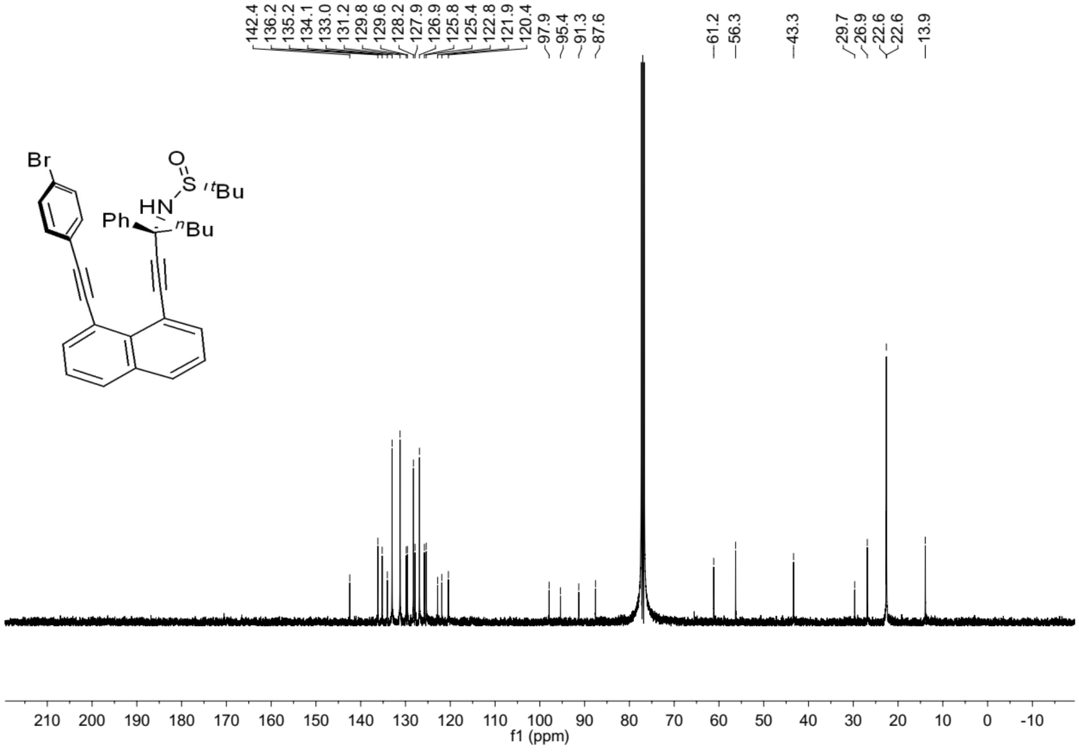

Figure S26. ^{13}C NMR Spectrum of Compound 7h ($CDCl_3$, 100 MHz)

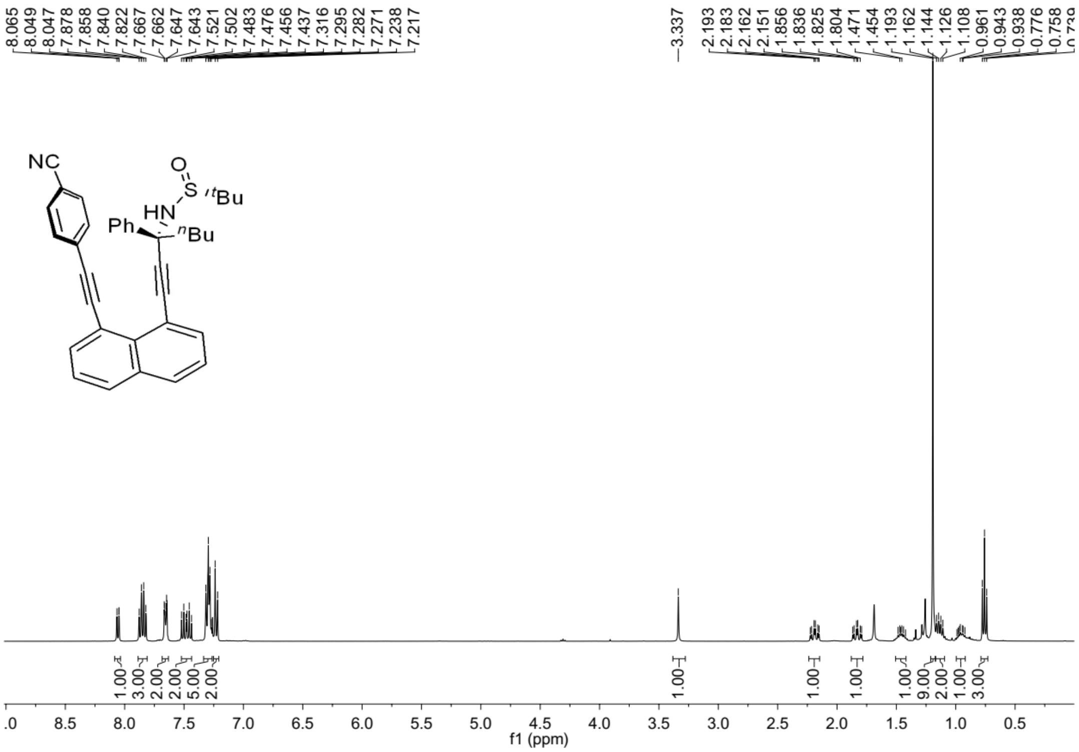

Figure S27. ^{1}H NMR Spectrum of Compound 7i ($CDCl_3$, 400 MHz)

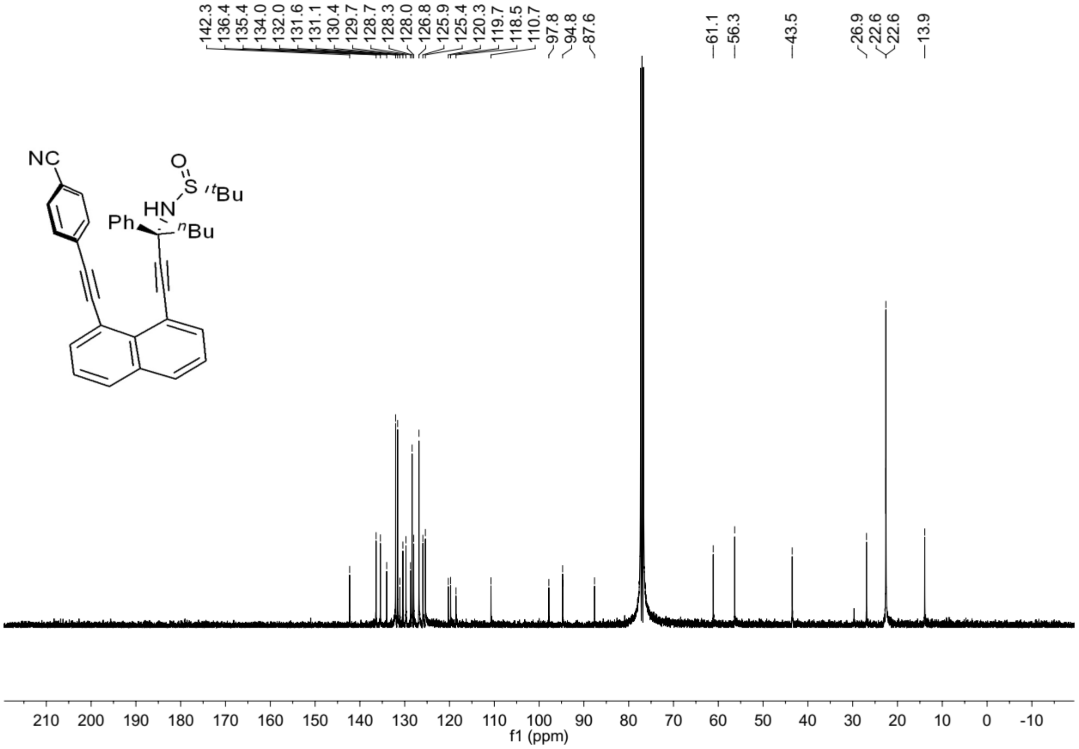

Figure S28. ^{13}C NMR Spectrum of Compound 7i ($CDCl_3$, 100 MHz)

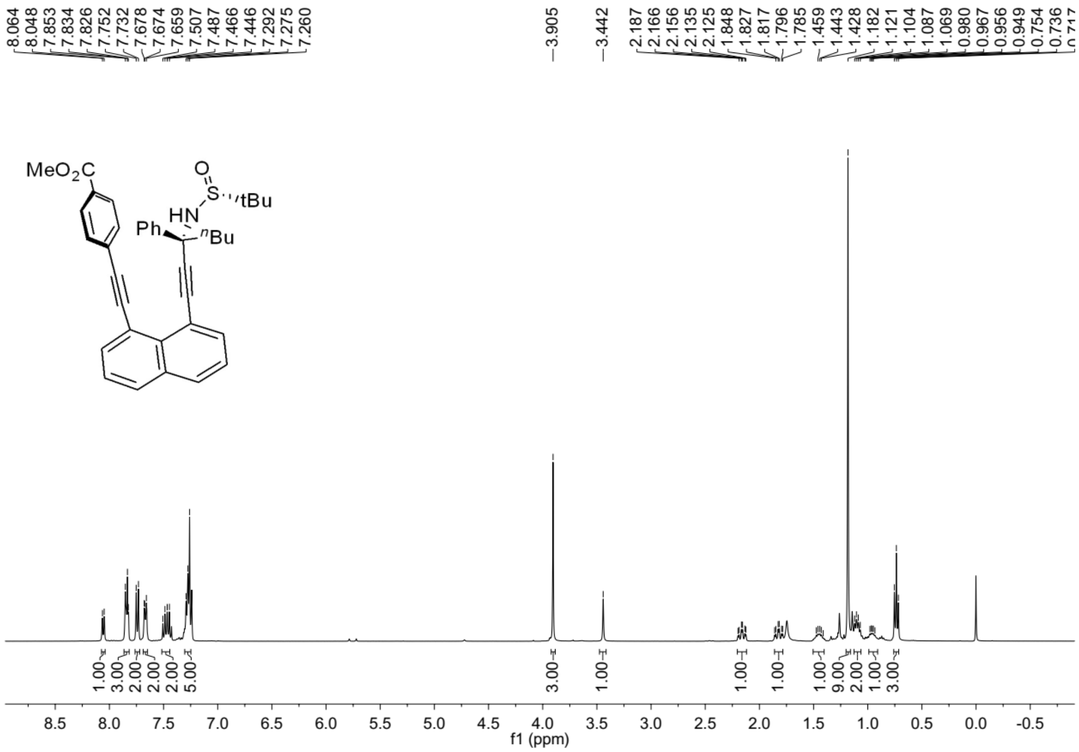

Figure S29. ^{1}**H NMR Spectrum of Compound 7j ($CDCl_3$, 400 MHz)**

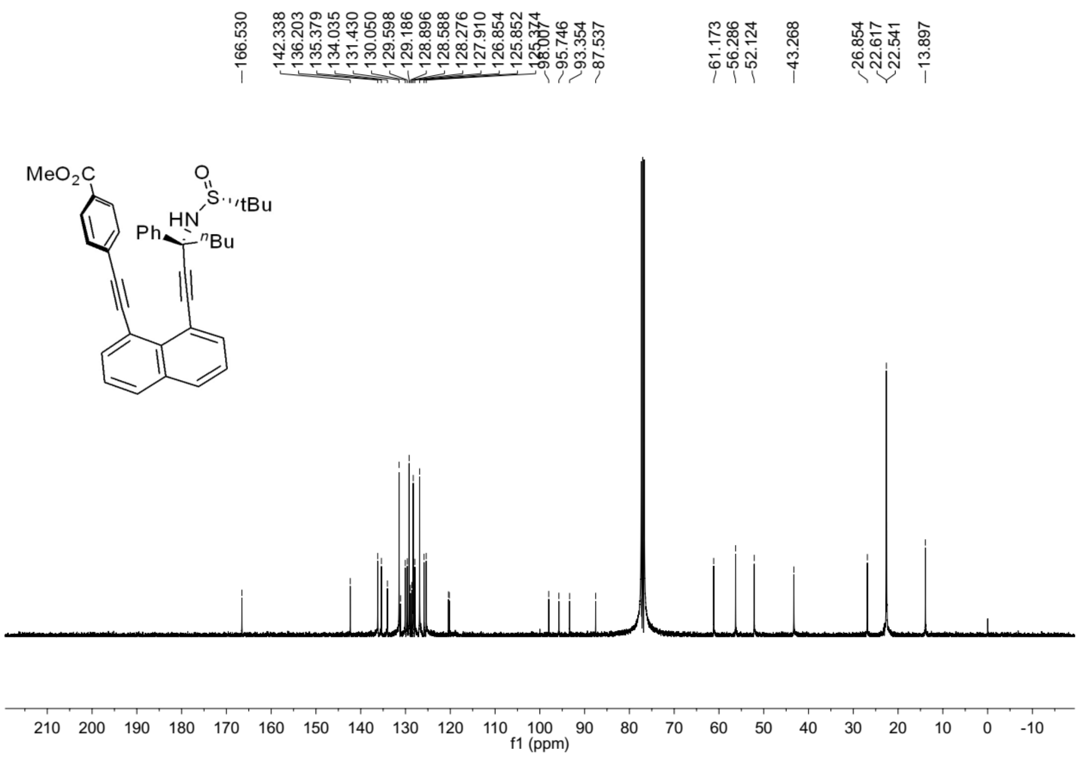

Figure S30. ^{13}**C NMR Spectrum of Compound 7j ($CDCl_3$, 100 MHz)**

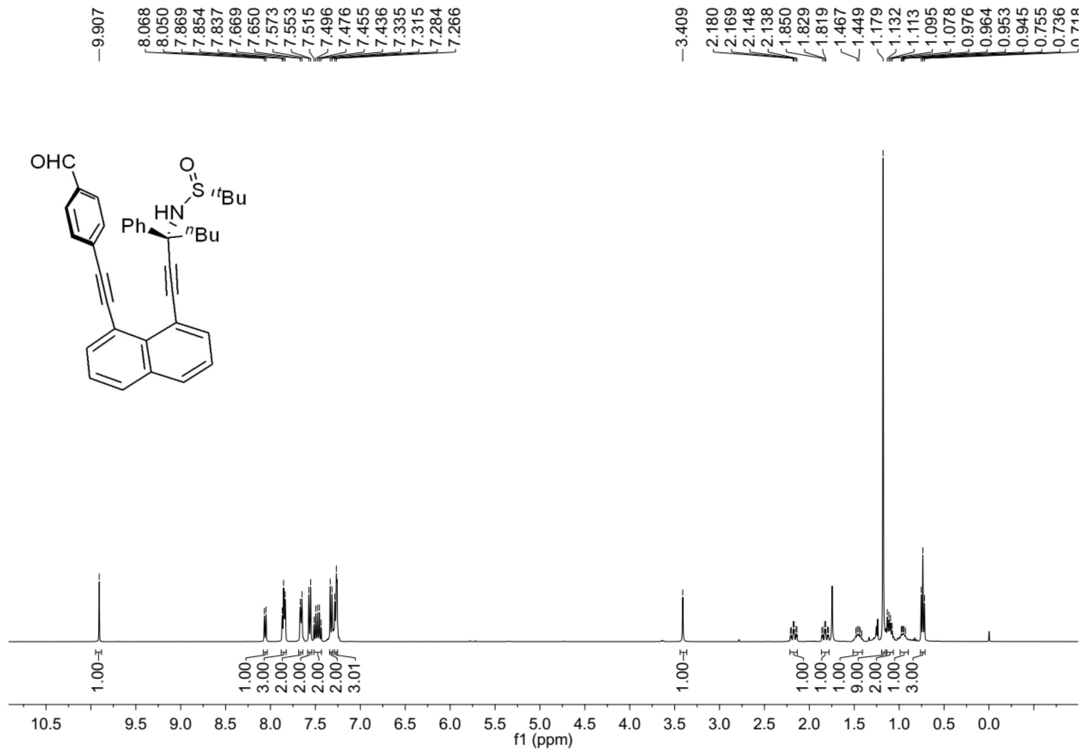

Figure S31. ^{1}H NMR Spectrum of Compound 7k ($CDCl_3$, 400 MHz)

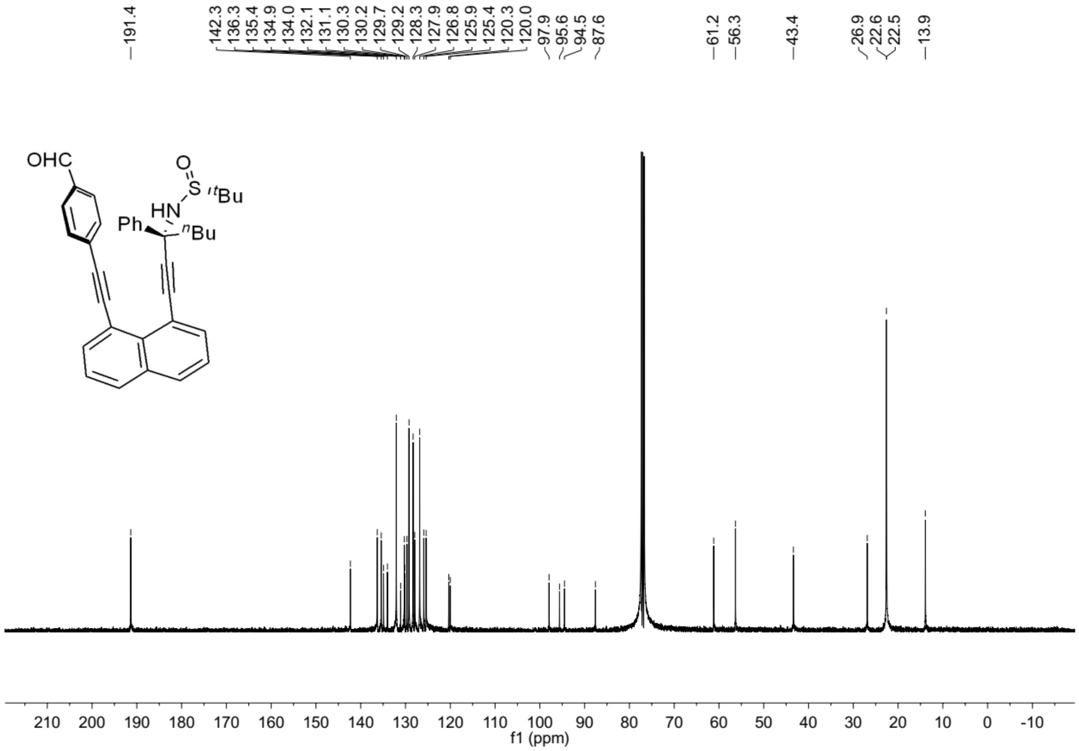

Figure S32. ^{13}C NMR Spectrum of Compound 7k ($CDCl_3$, 100 MHz)

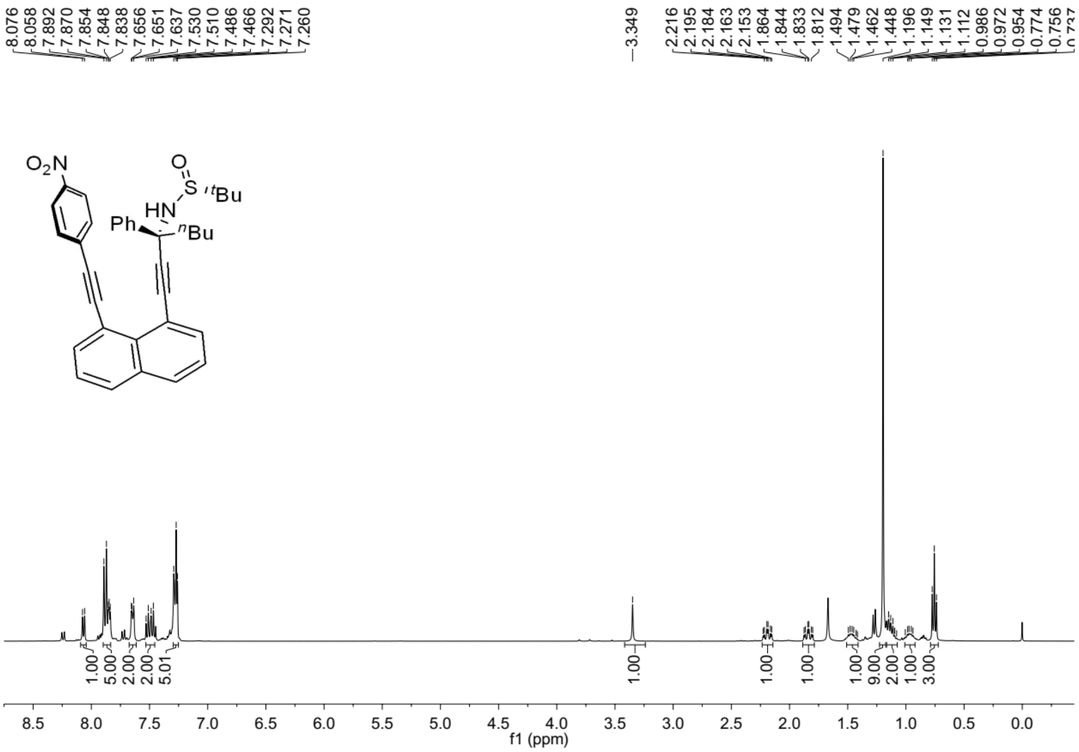

Figure S33. ^{1}**H NMR Spectrum of Compound 7l (CDCl$_3$, 400 MHz)**

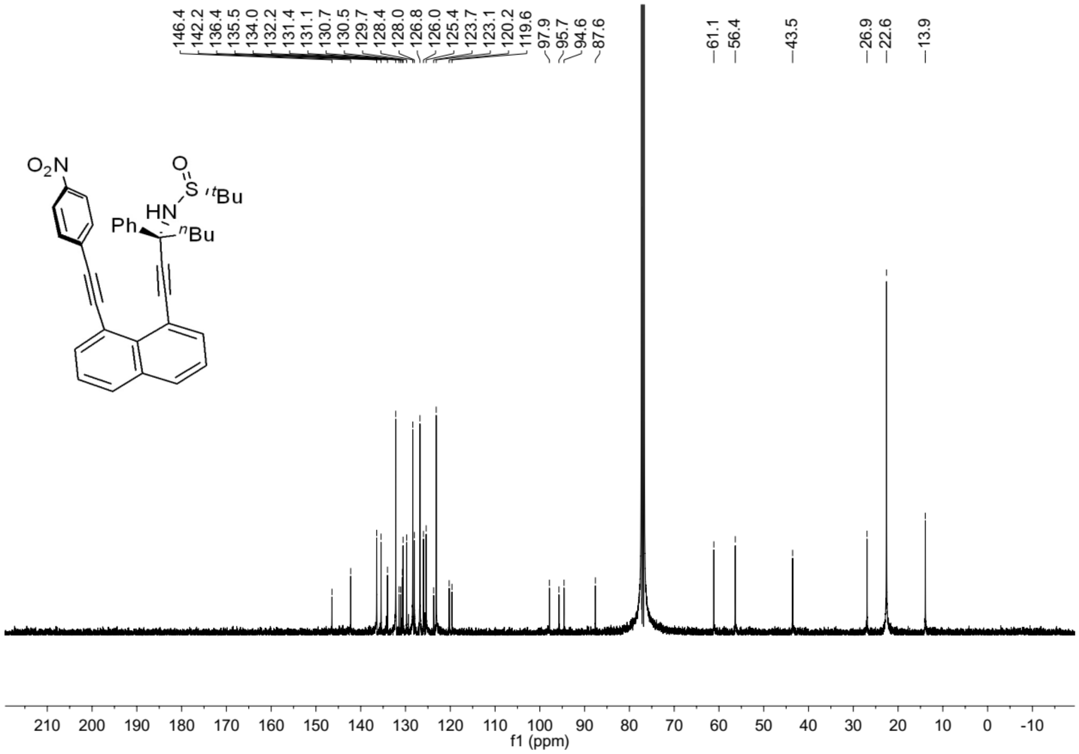

Figure S34. ^{13}**C NMR Spectrum of Compound 7l (CDCl$_3$, 100 MHz)**

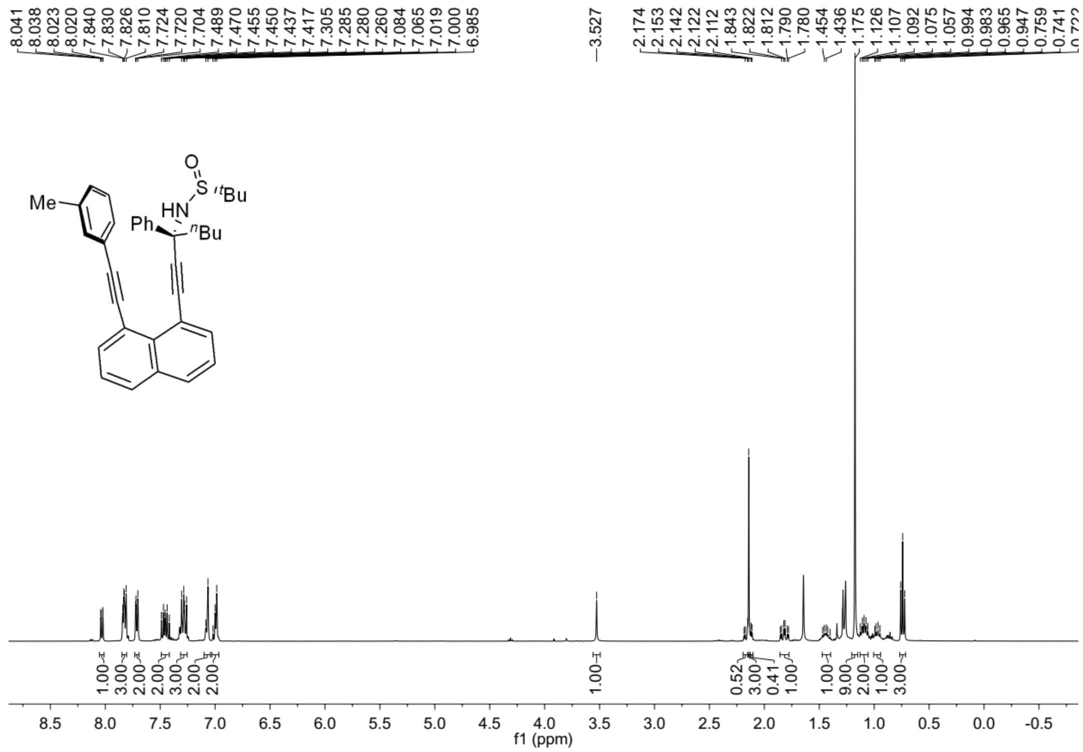

Figure S35. ^{1}H NMR Spectrum of Compound 7m ($CDCl_3$, 400 MHz)

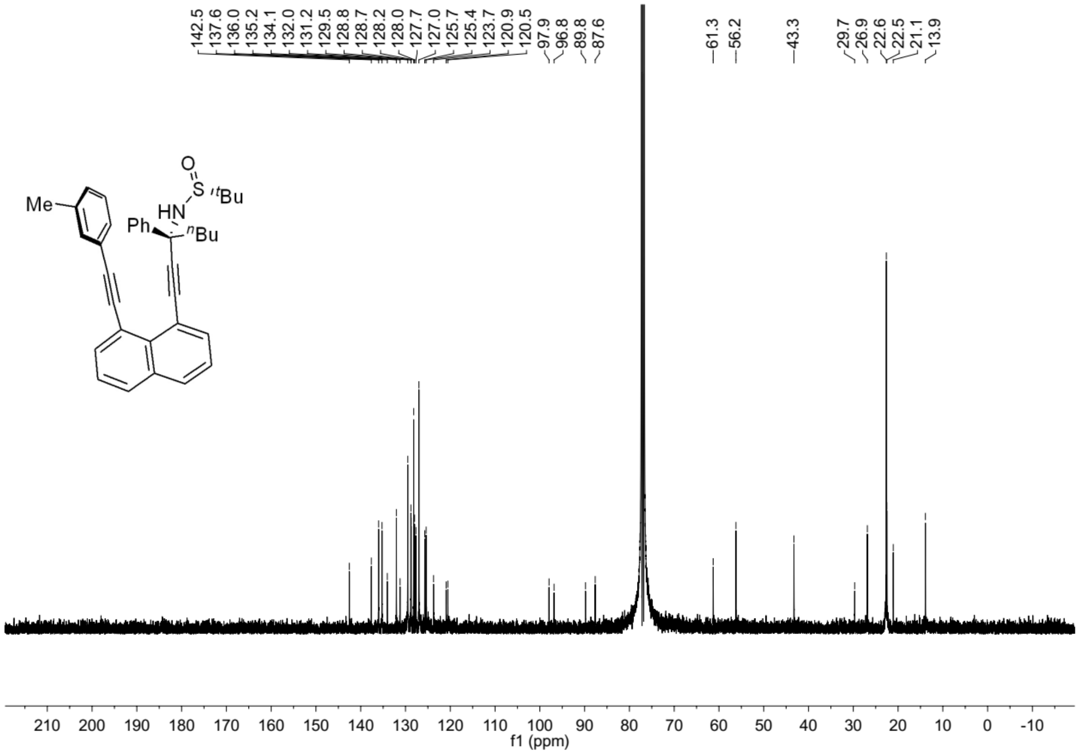

Figure S36. ^{13}C NMR Spectrum of Compound 7m ($CDCl_3$, 100 MHz)

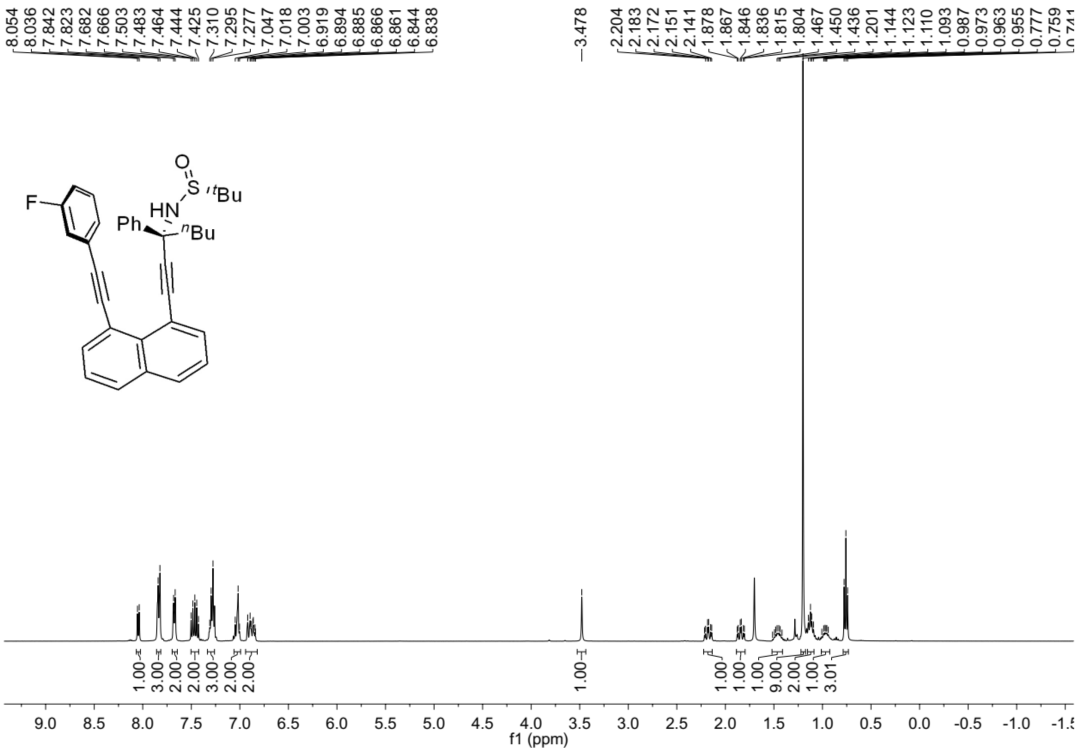

Figure S37. ^{1}H NMR Spectrum of Compound 7n ($CDCl_3$, 400 MHz)

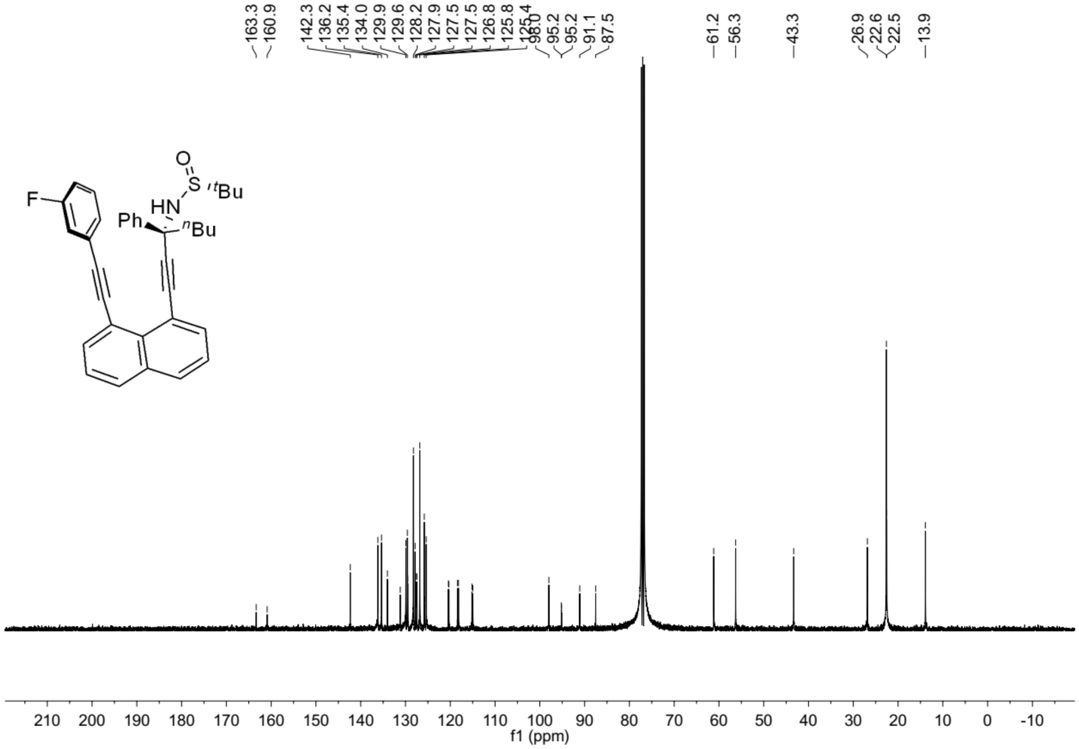

Figure S38. ^{13}C NMR Spectrum of Compound 7n ($CDCl_3$, 100 MHz)

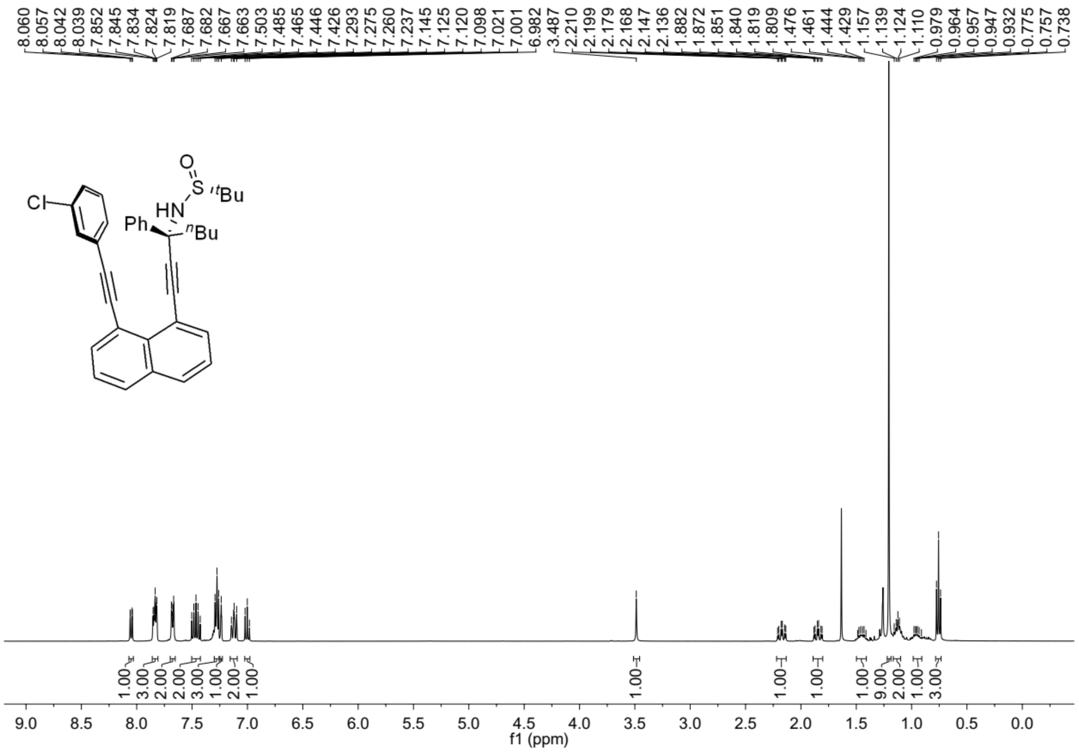

Figure S39. ^{1}H NMR Spectrum of Compound 7o ($CDCl_3$, 400 MHz)

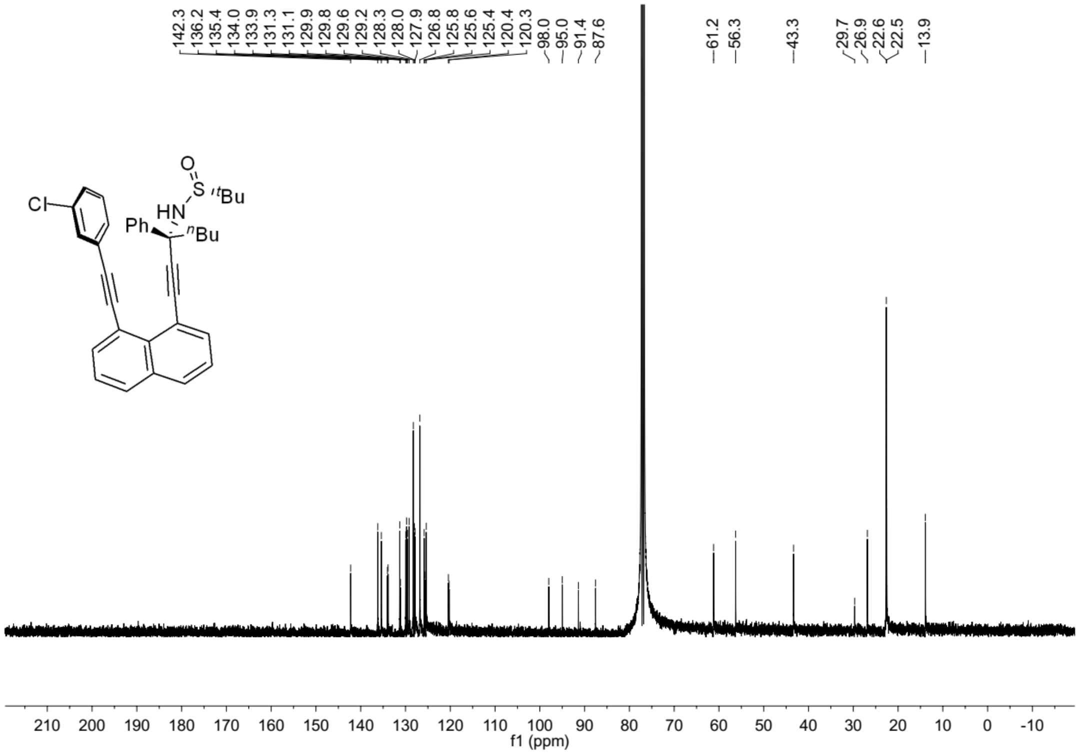

Figure S40. ^{13}C NMR Spectrum of Compound 7o ($CDCl_3$, 100 MHz)

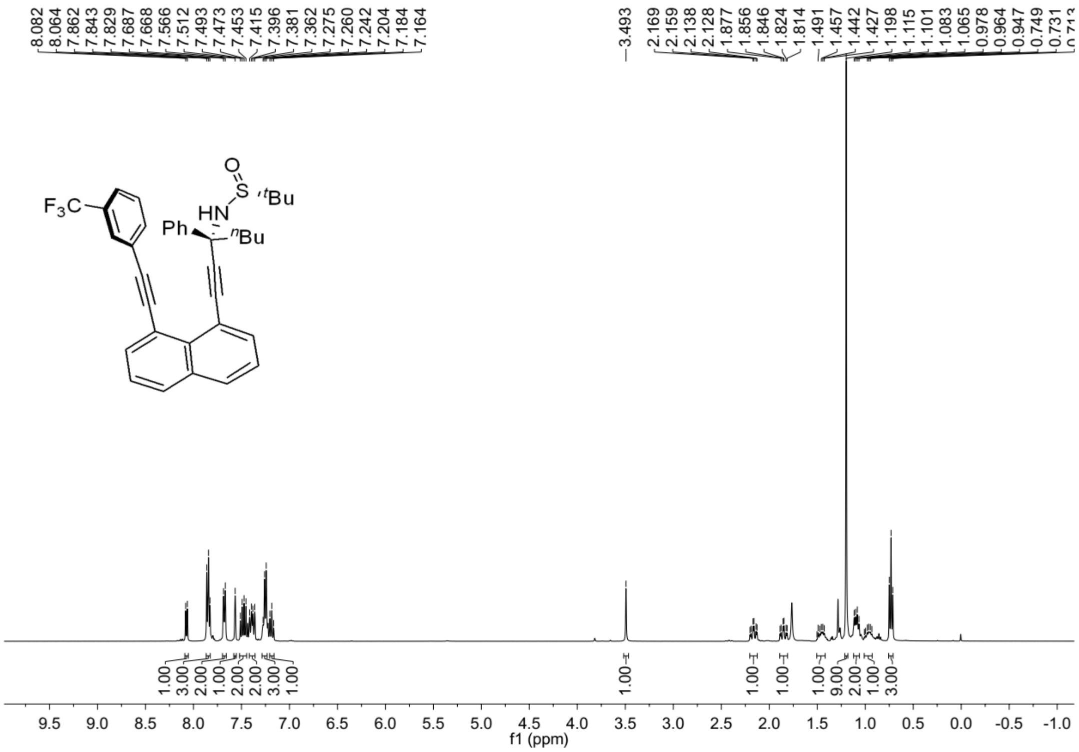

Figure S41. ^{1}H NMR Spectrum of Compound 7p ($CDCl_3$, 400 MHz)

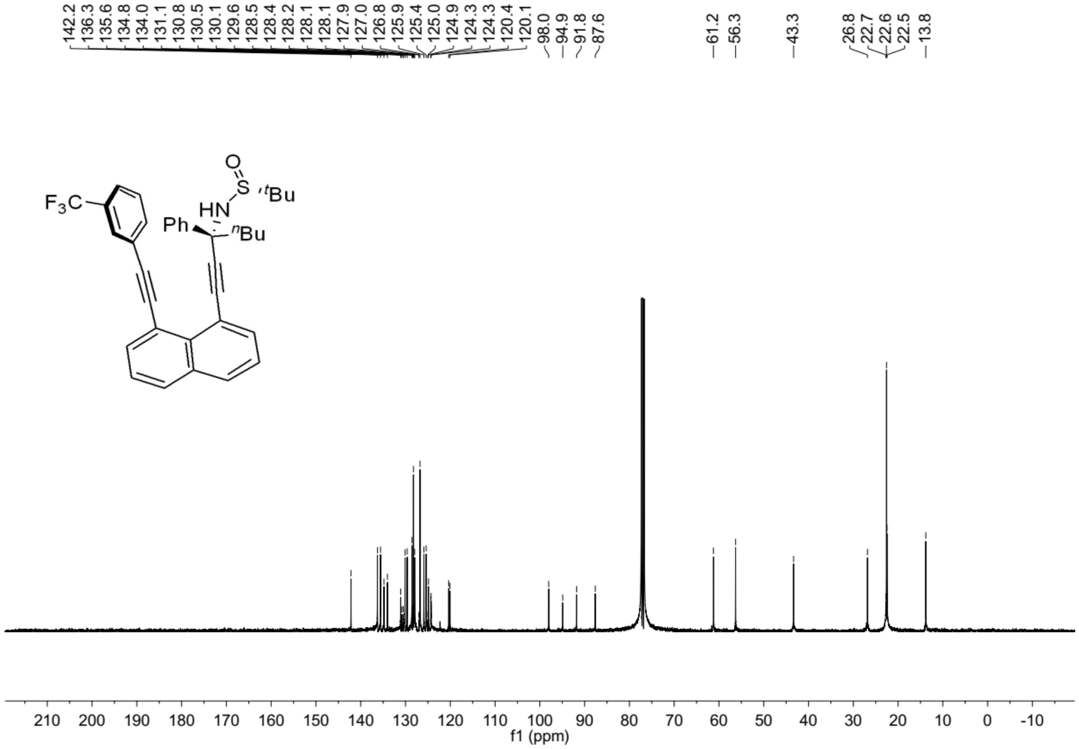

Figure S42. ^{13}C NMR Spectrum of Compound 7p ($CDCl_3$, 100 MHz)

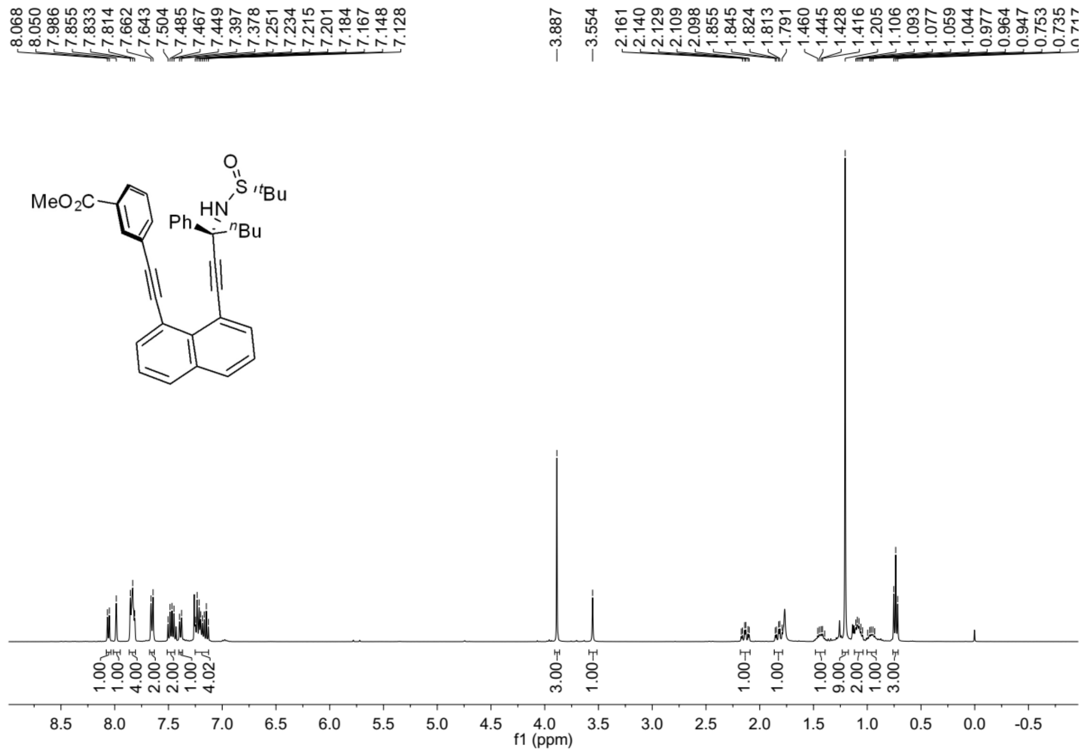

Figure S43. ^{1}H NMR Spectrum of Compound 7q ($CDCl_3$, 400 MHz)

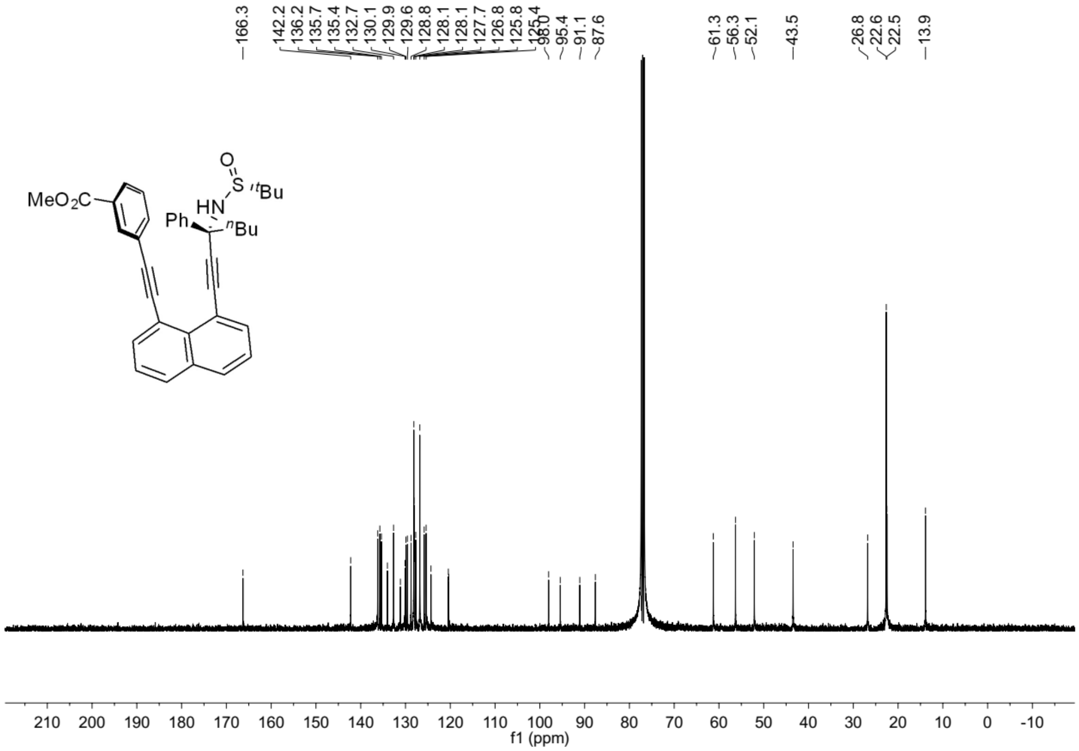

Figure S44. ^{13}C NMR Spectrum of Compound 7q ($CDCl_3$, 100 MHz)

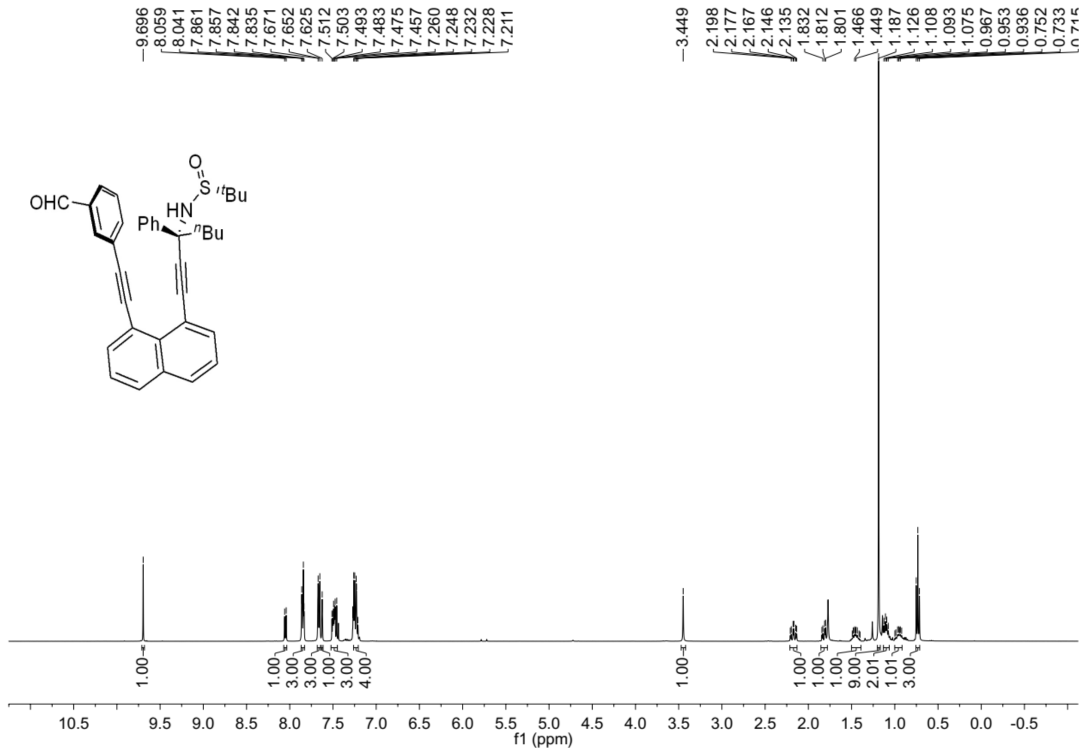

Figure S45. ^{1}H NMR Spectrum of Compound 7r (CDCl$_3$, 400 MHz)

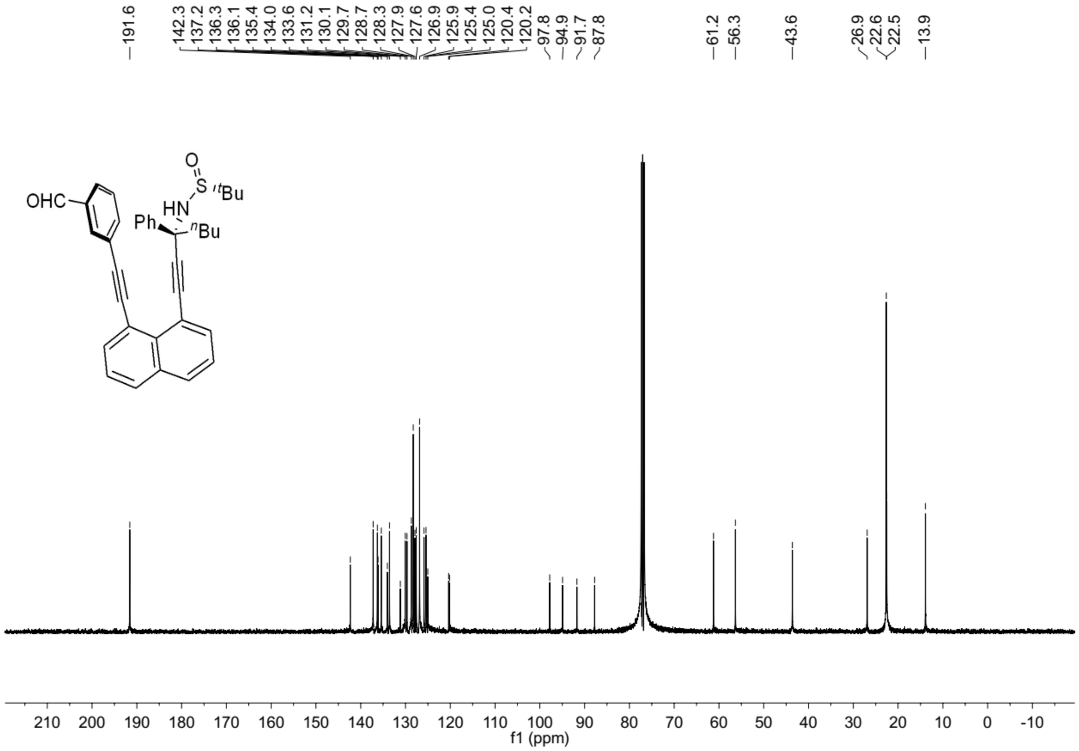

Figure S46. ^{13}C NMR Spectrum of Compound 7r (CDCl$_3$, 100 MHz)

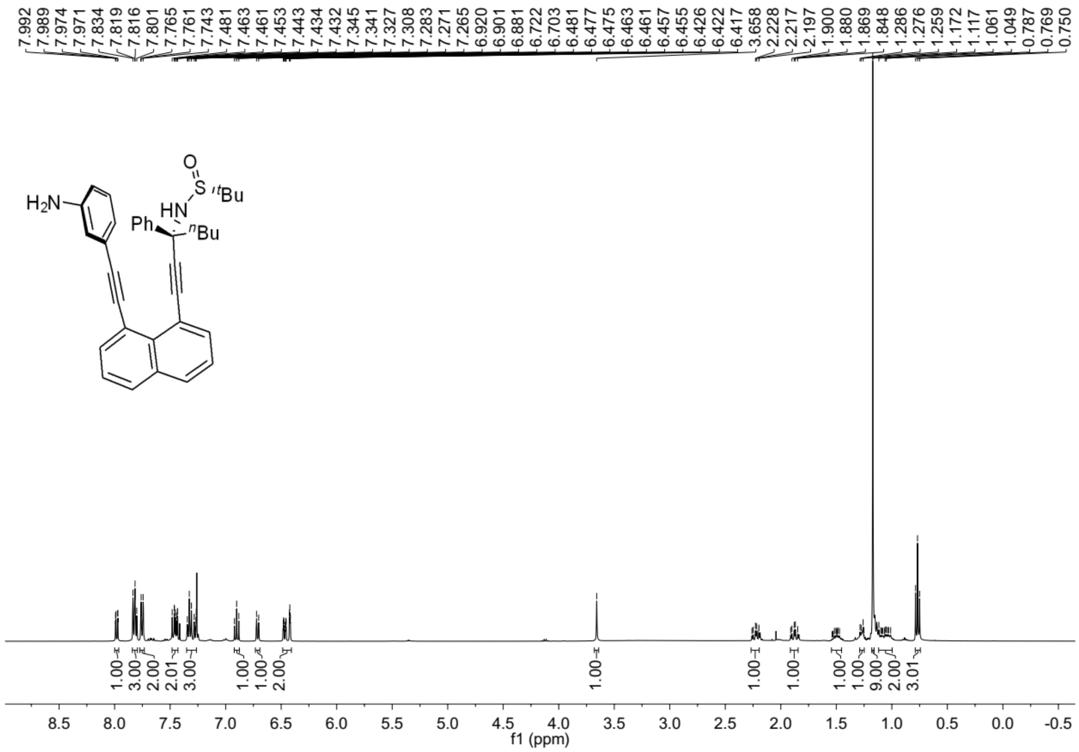

Figure S47. ^{1}**H NMR Spectrum of Compound 7s (**$CDCl_3$**, 400 MHz)**

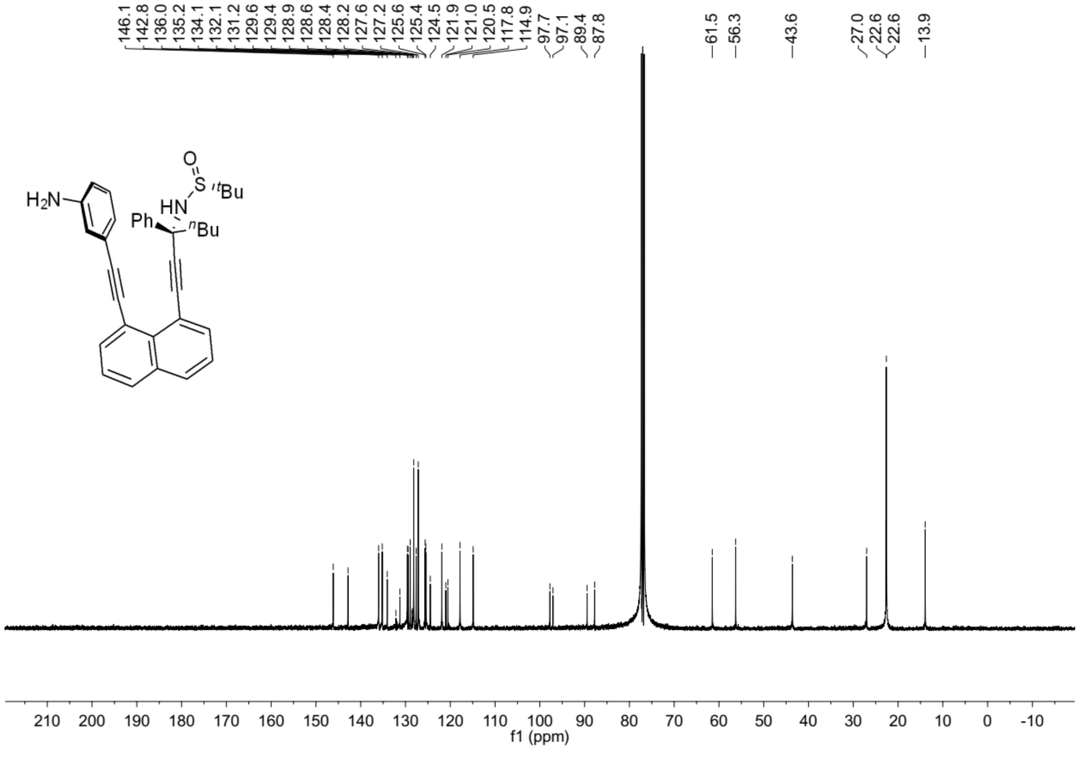

Figure S48. ^{13}**C NMR Spectrum of Compound 7s (**$CDCl_3$**, 100 MHz)**

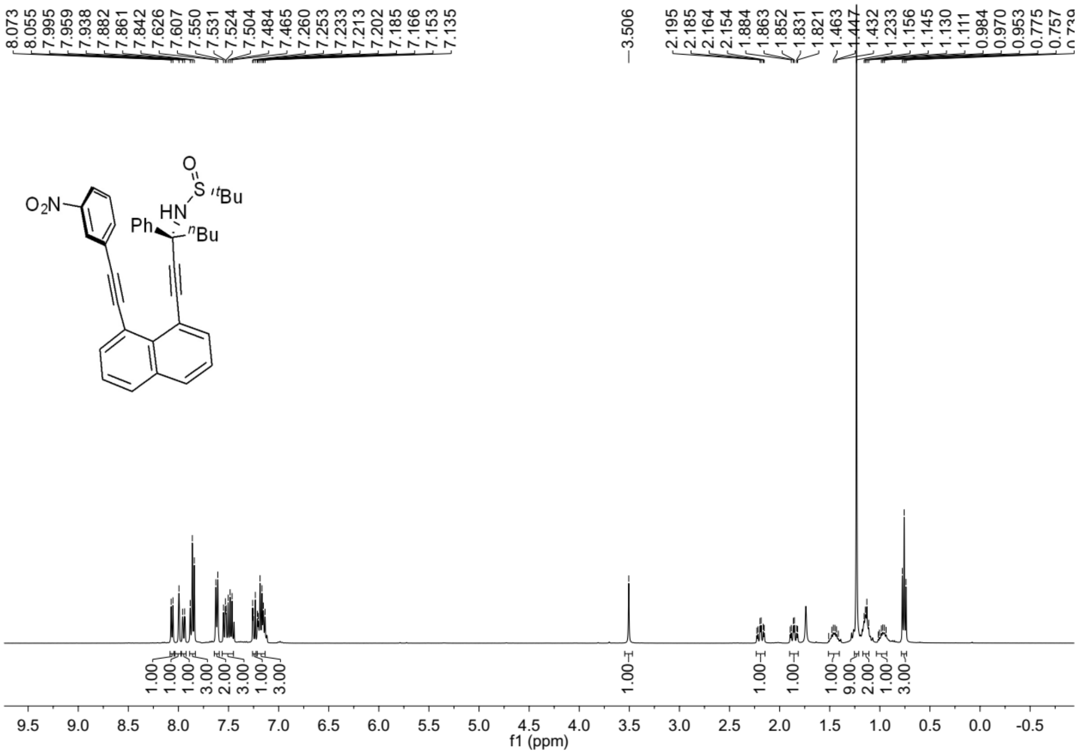

Figure S49. ^{1}H NMR Spectrum of Compound 7t ($CDCl_3$, 400 MHz)

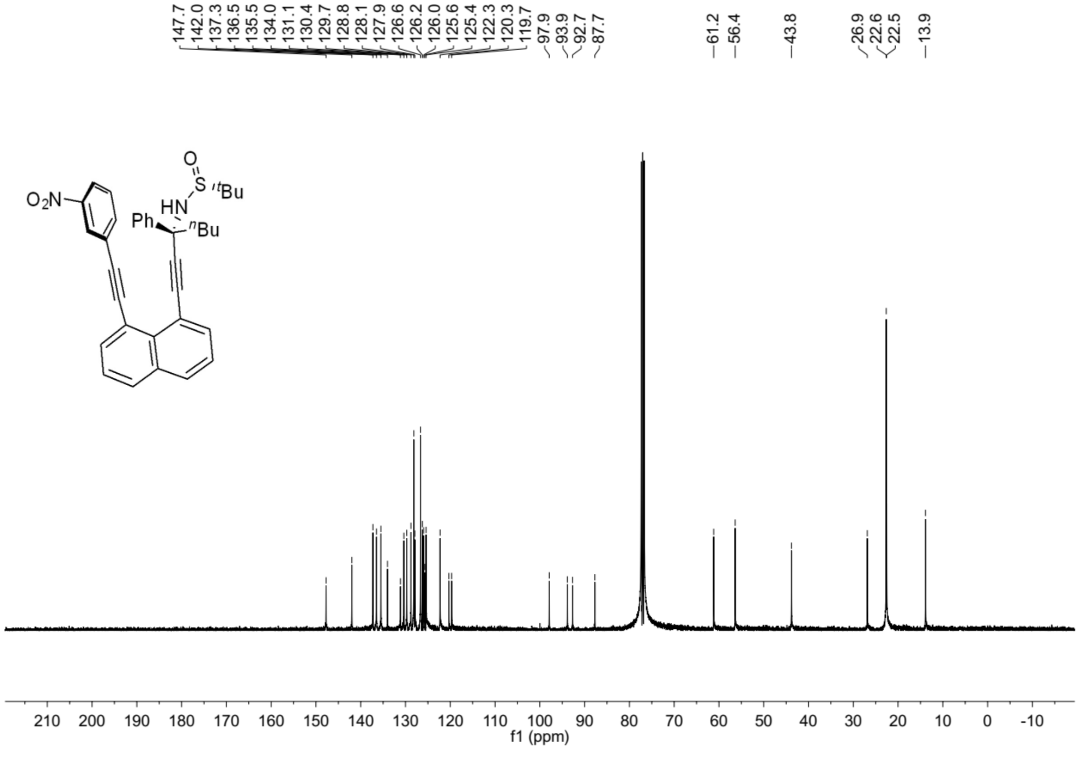

Figure S50. ^{13}C NMR Spectrum of Compound 7t ($CDCl_3$, 100 MHz)

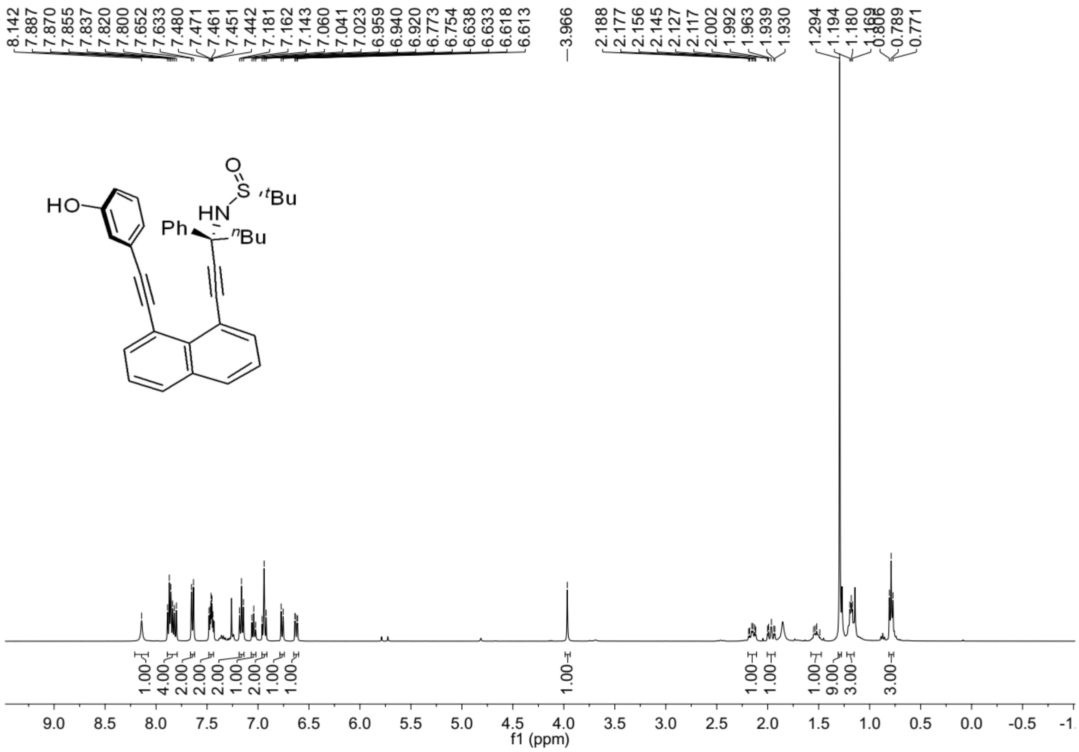

Figure S51. ^{1}H NMR Spectrum of Compound 7u ($CDCl_3$, 400 MHz)

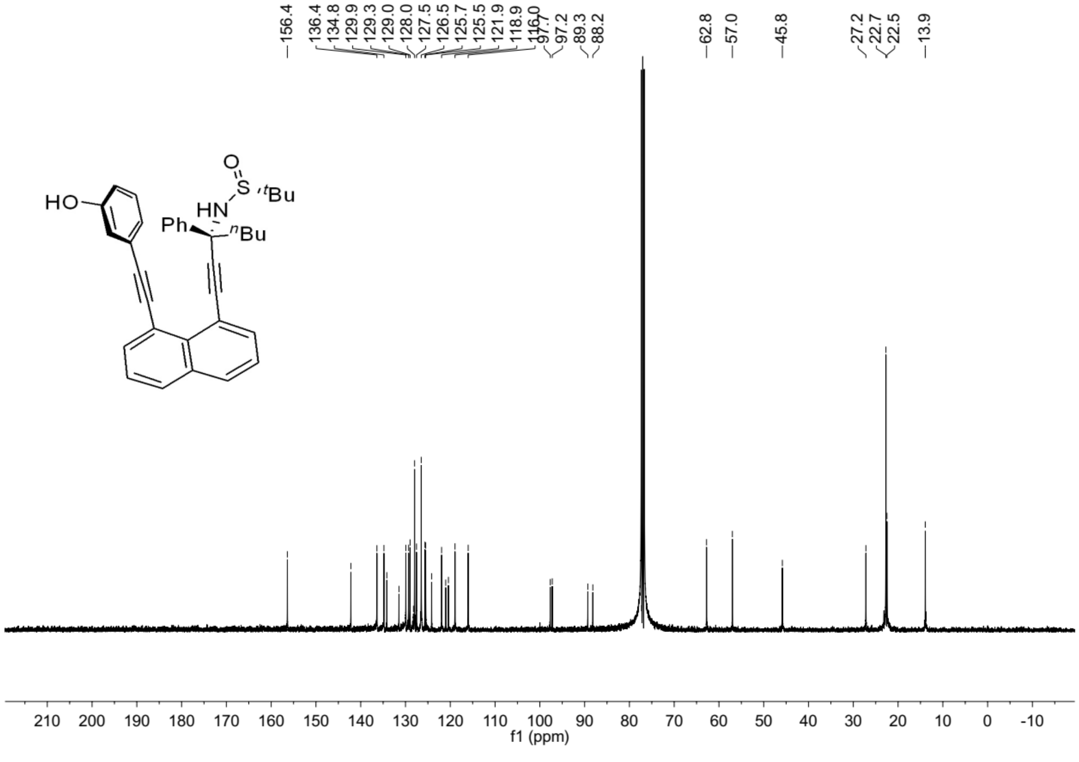

Figure S52. ^{13}C NMR Spectrum of Compound 7u ($CDCl_3$, 100 MHz)

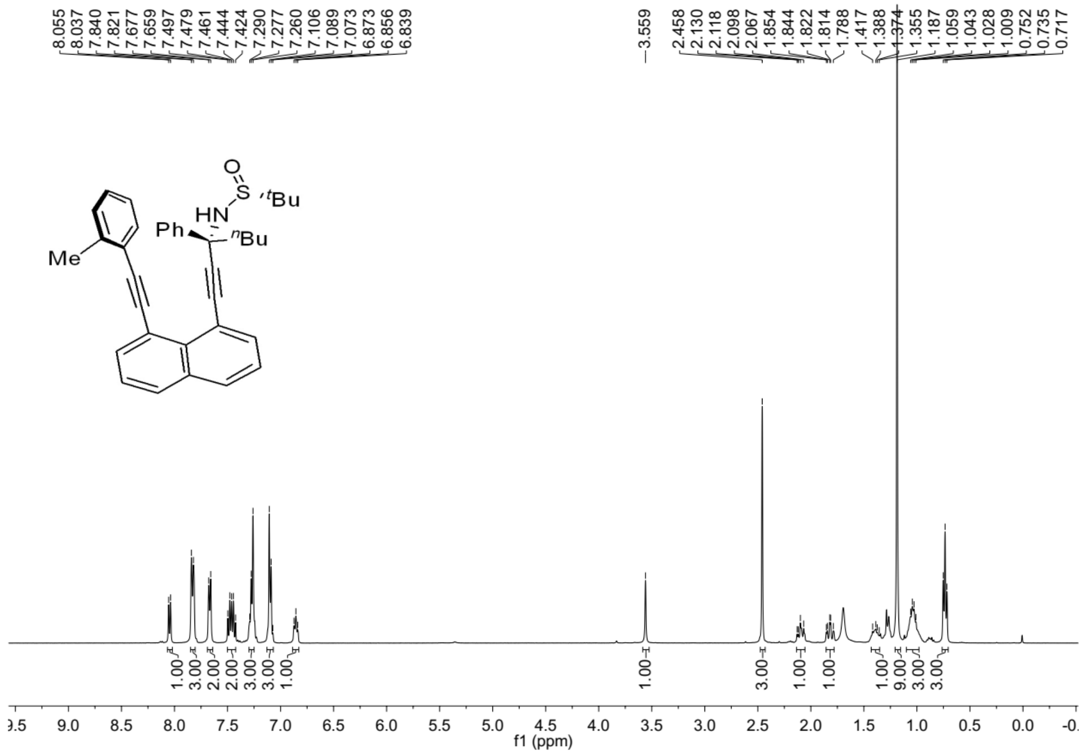

Figure S53. ^{1}H NMR Spectrum of Compound 7v ($CDCl_3$, 400 MHz)

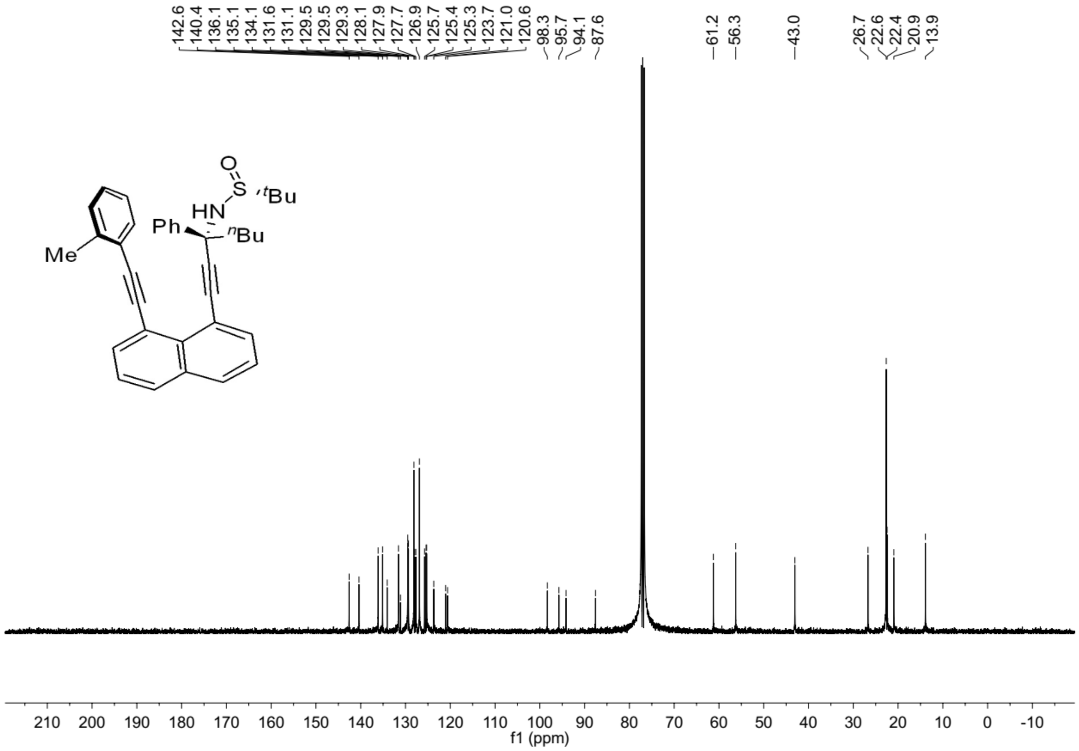

Figure S54. ^{13}C NMR Spectrum of Compound 7v ($CDCl_3$, 100 MHz)

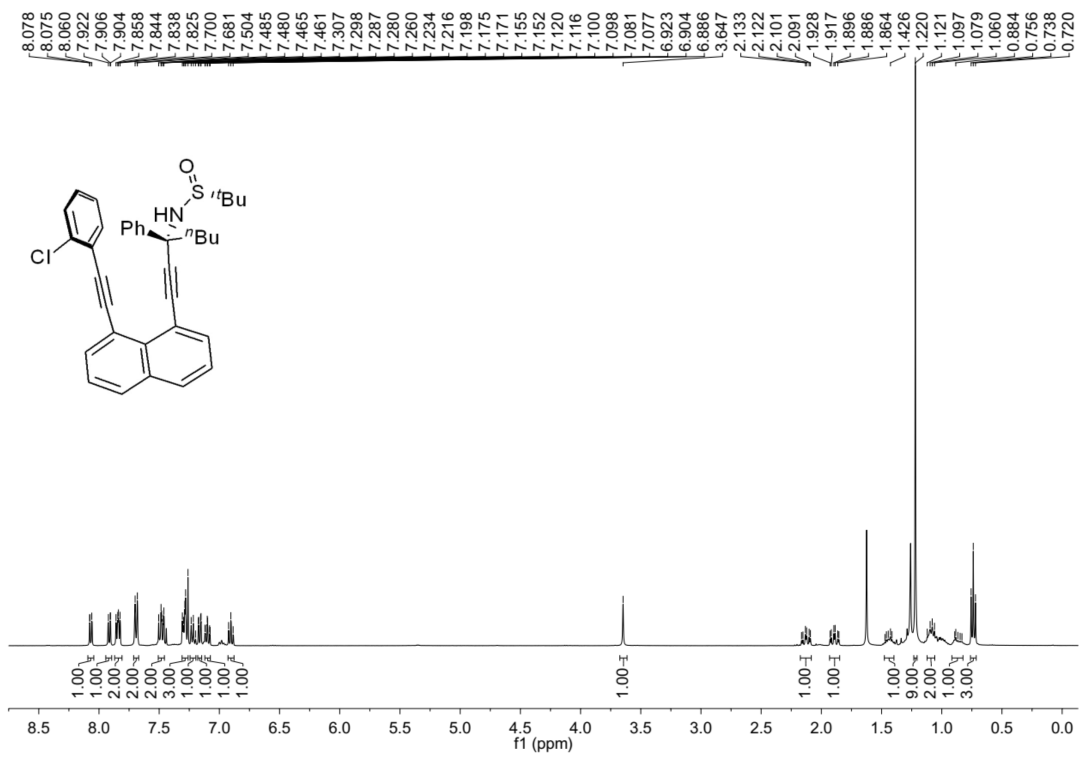

Figure S55. ^{1}H NMR Spectrum of Compound 7w ($CDCl_3$, 400 MHz)

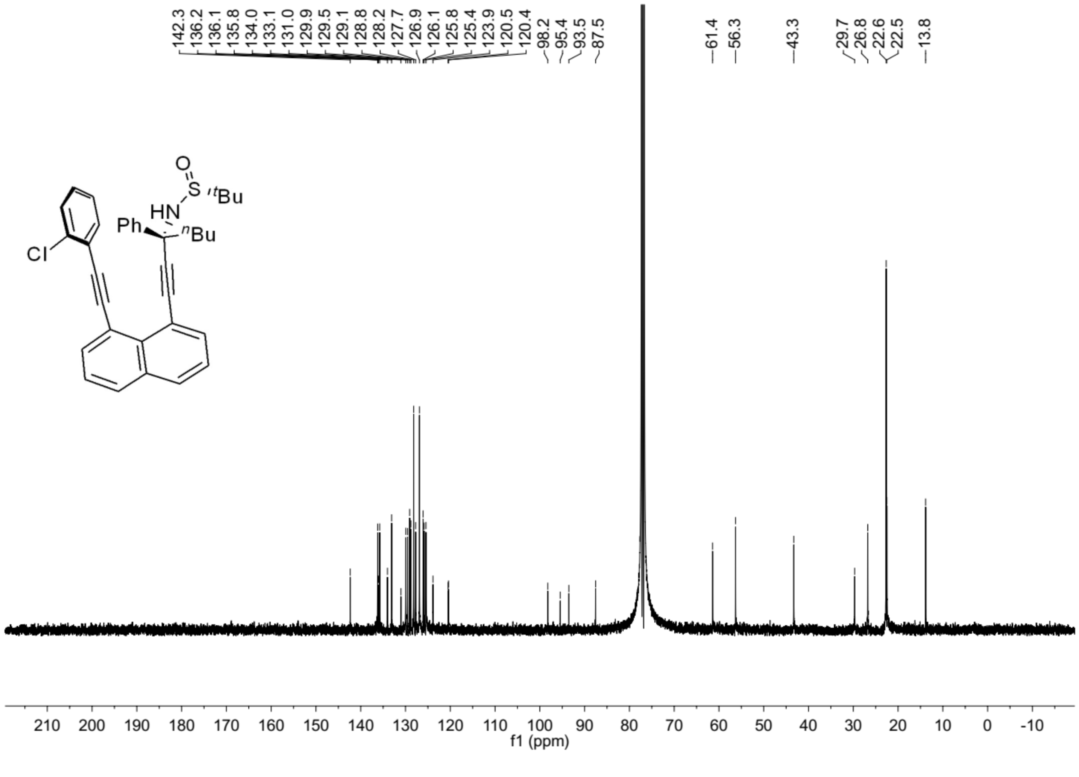

Figure S56. ^{13}C NMR Spectrum of Compound 7w ($CDCl_3$, 100 MHz)

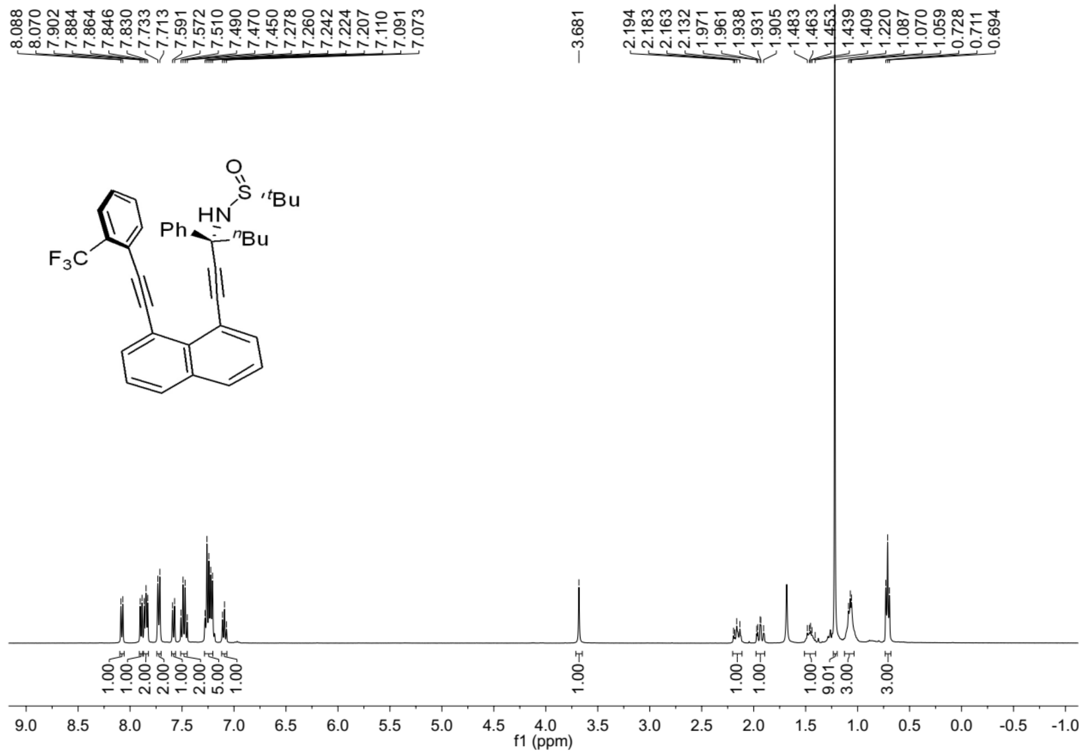

Figure S57. ^{1}H NMR Spectrum of Compound 7x ($CDCl_3$, 400 MHz)

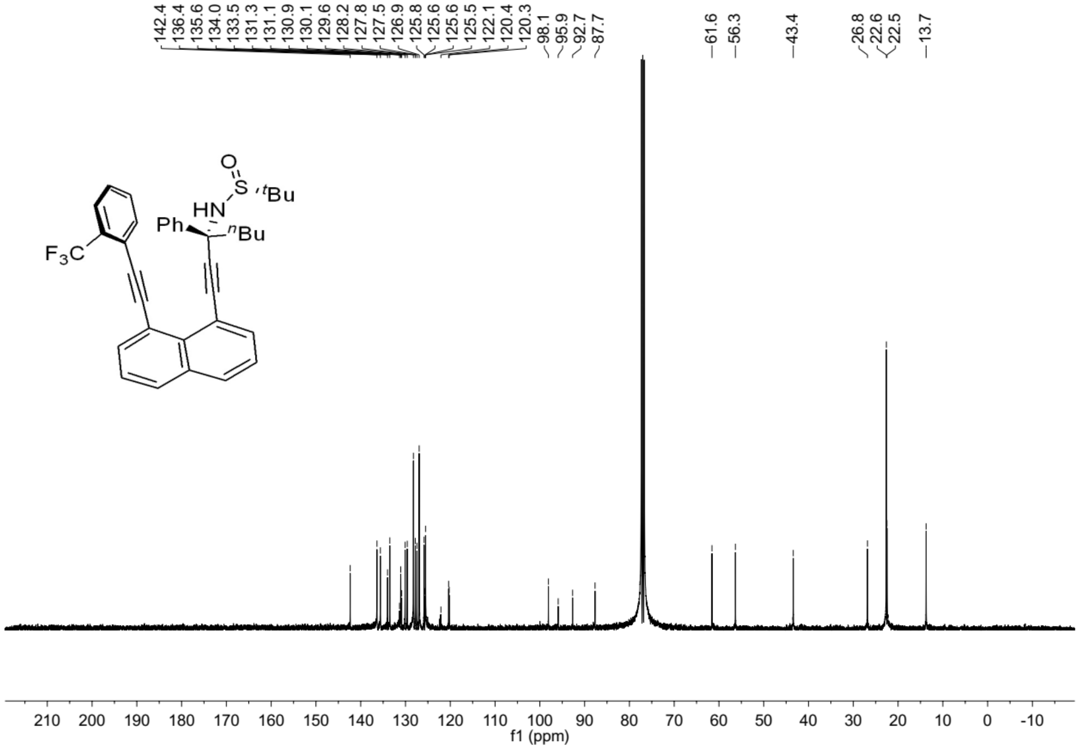

Figure S58. ^{13}C NMR Spectrum of Compound 7x ($CDCl_3$, 100 MHz)

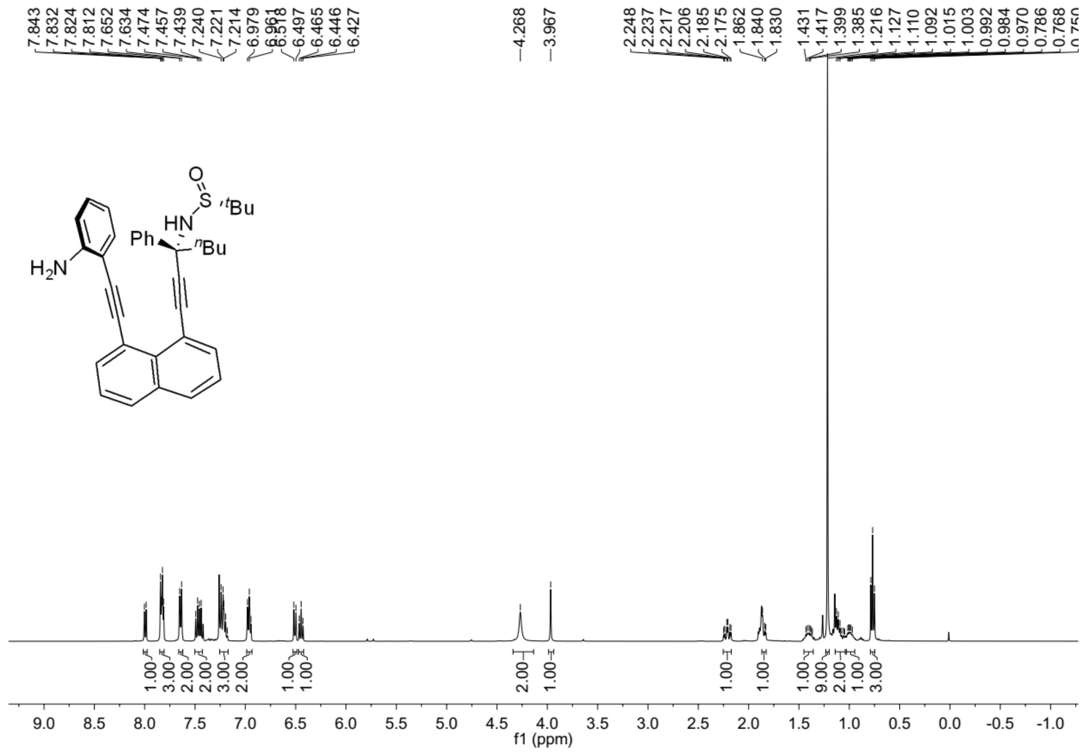

Figure S59. ^{1}**H NMR Spectrum of Compound 7y (CDCl**$_3$**, 400 MHz)**

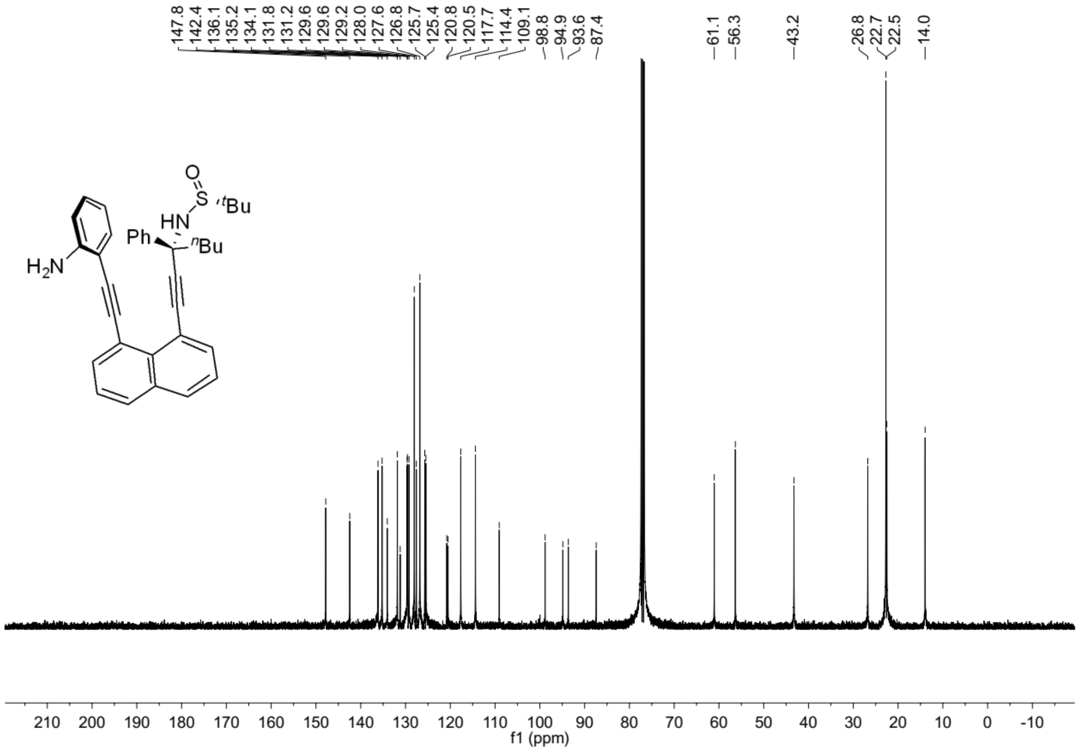

Figure S60. ^{13}**C NMR Spectrum of Compound 7y (CDCl**$_3$**, 100 MHz)**

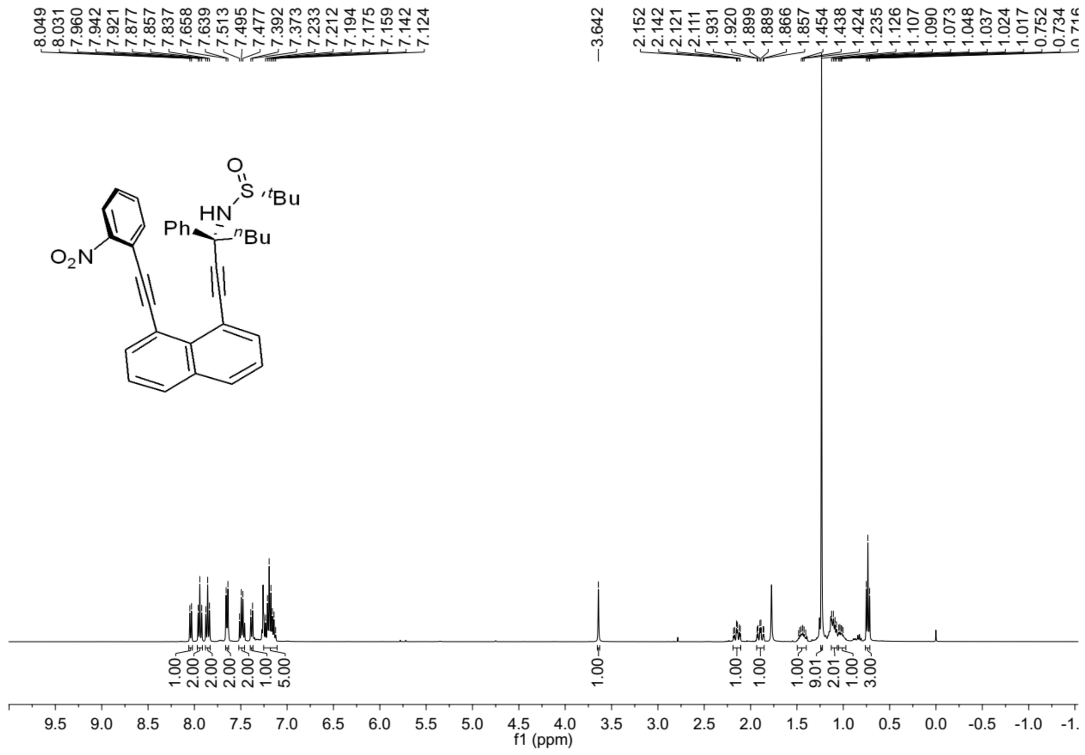

Figure S61. ^{1}**H NMR Spectrum of Compound 7z (CDCl$_3$, 400 MHz)**

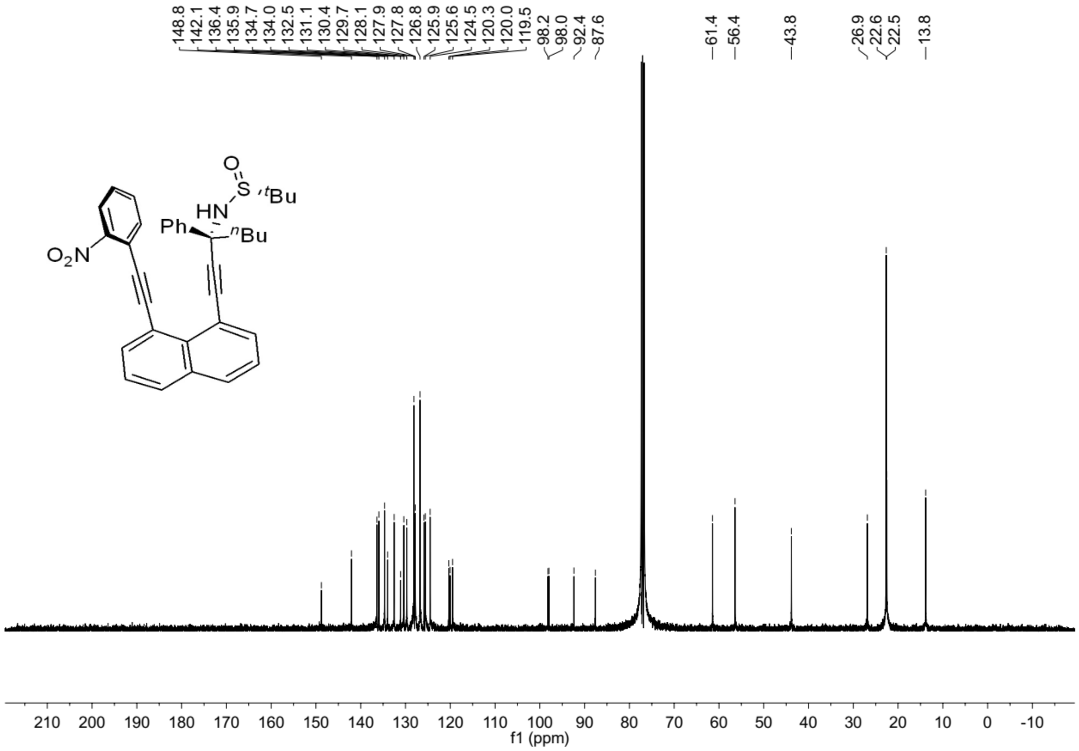

Figure S62. ^{13}**C NMR Spectrum of Compound 7z (CDCl$_3$, 100 MHz)**

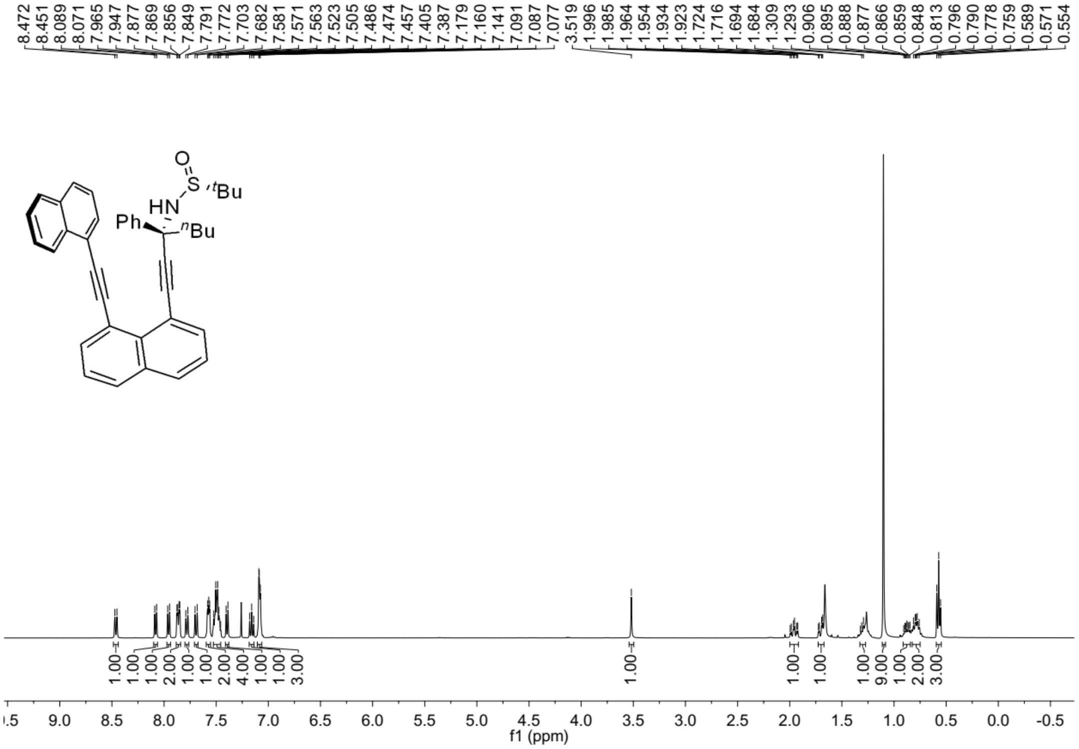

Figure S63. **^{1}H NMR Spectrum of Compound 7aa (CDCl$_3$, 400 MHz)**

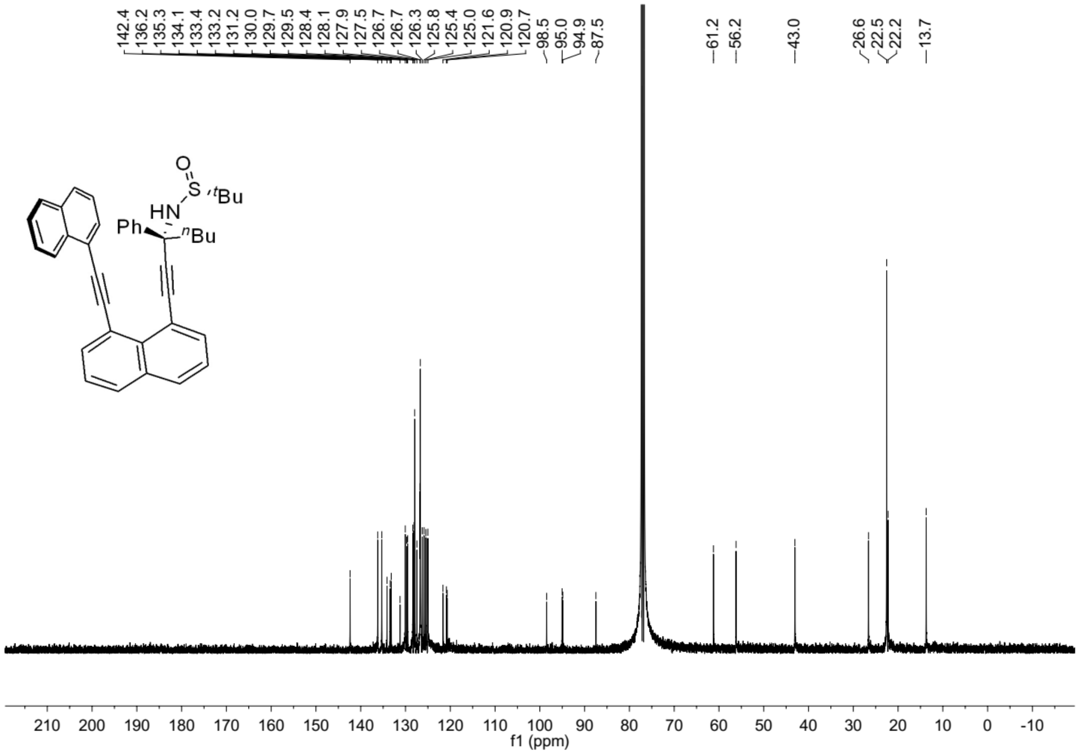

Figure S64. **^{13}C NMR Spectrum of Compound 7aa (CDCl$_3$, 100 MHz)**

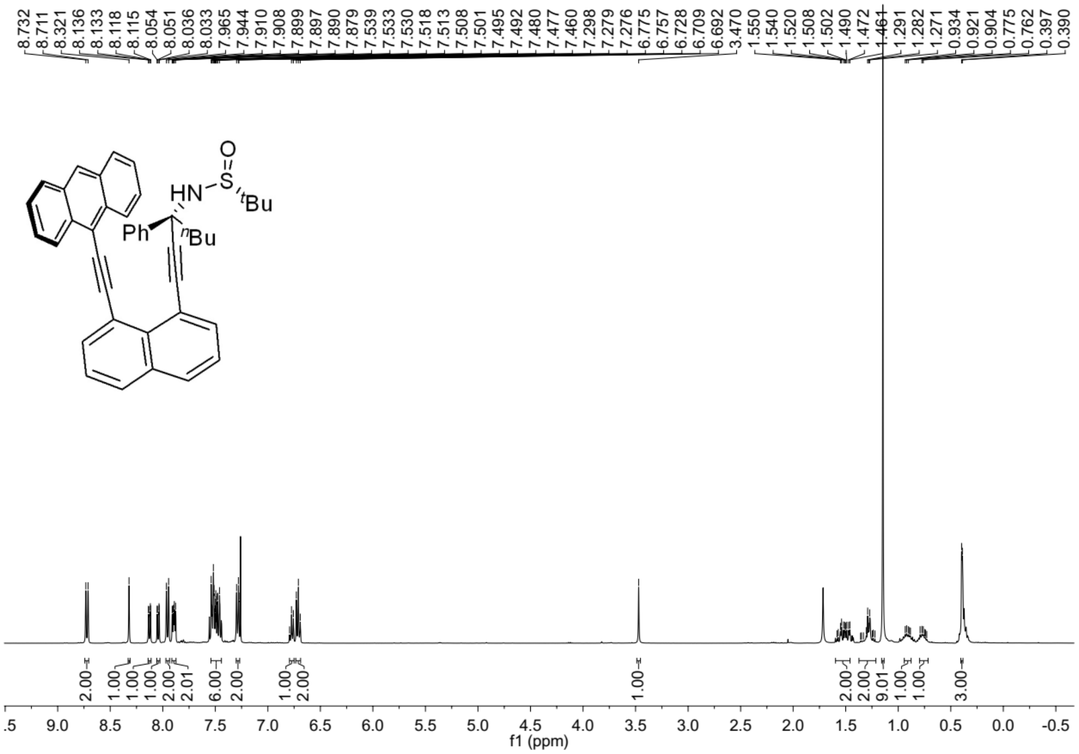

Figure S65. ^{1}H NMR Spectrum of Compound 7ab ($CDCl_3$, 400 MHz)

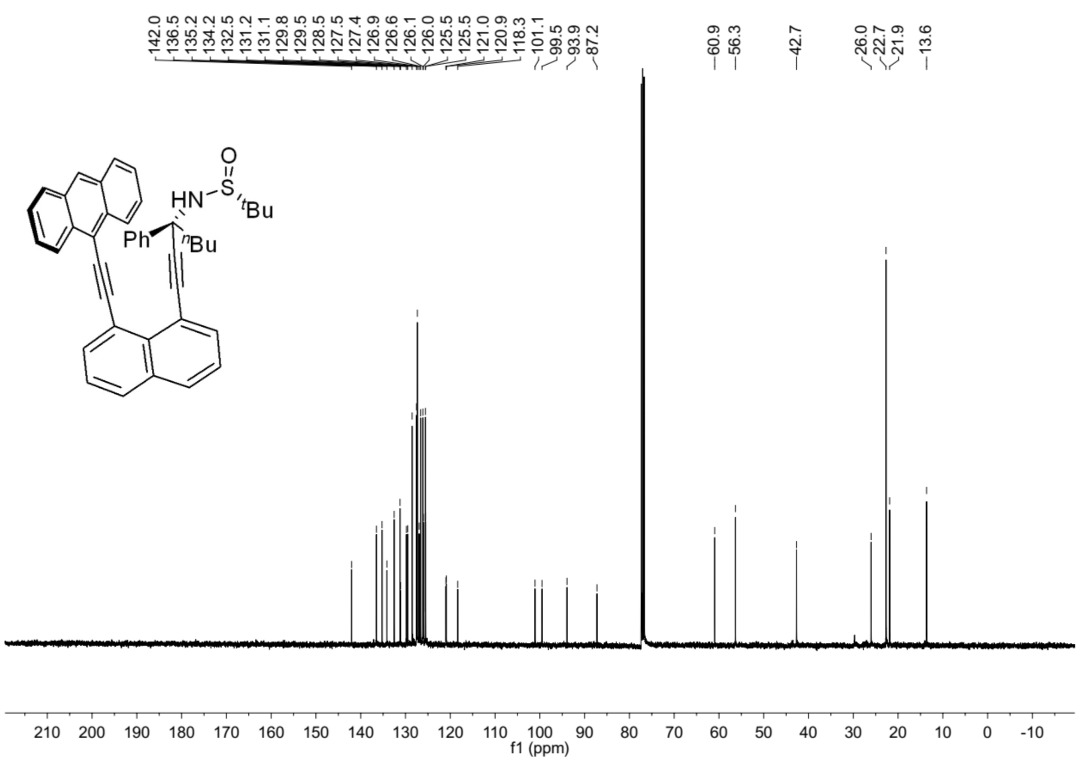

Figure S66. ^{13}C NMR Spectrum of Compound 7ab ($CDCl_3$, 100 MHz)

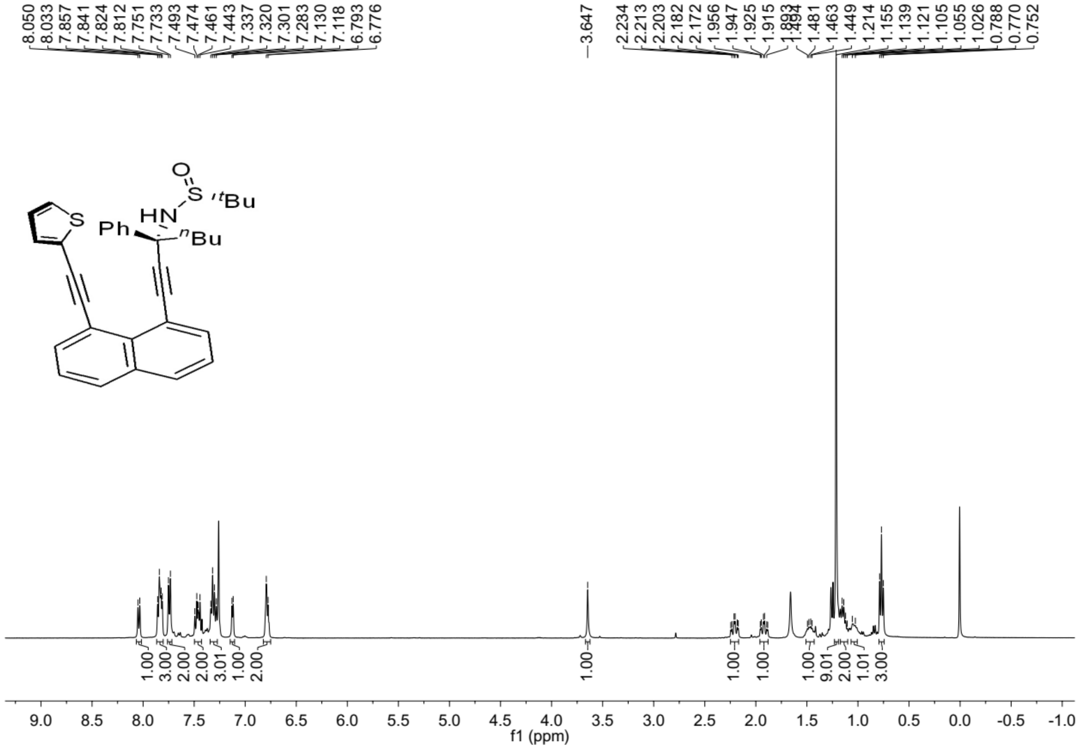

Figure S67. **^{1}H NMR Spectrum of Compound 7ac ($CDCl_3$, 400 MHz)**

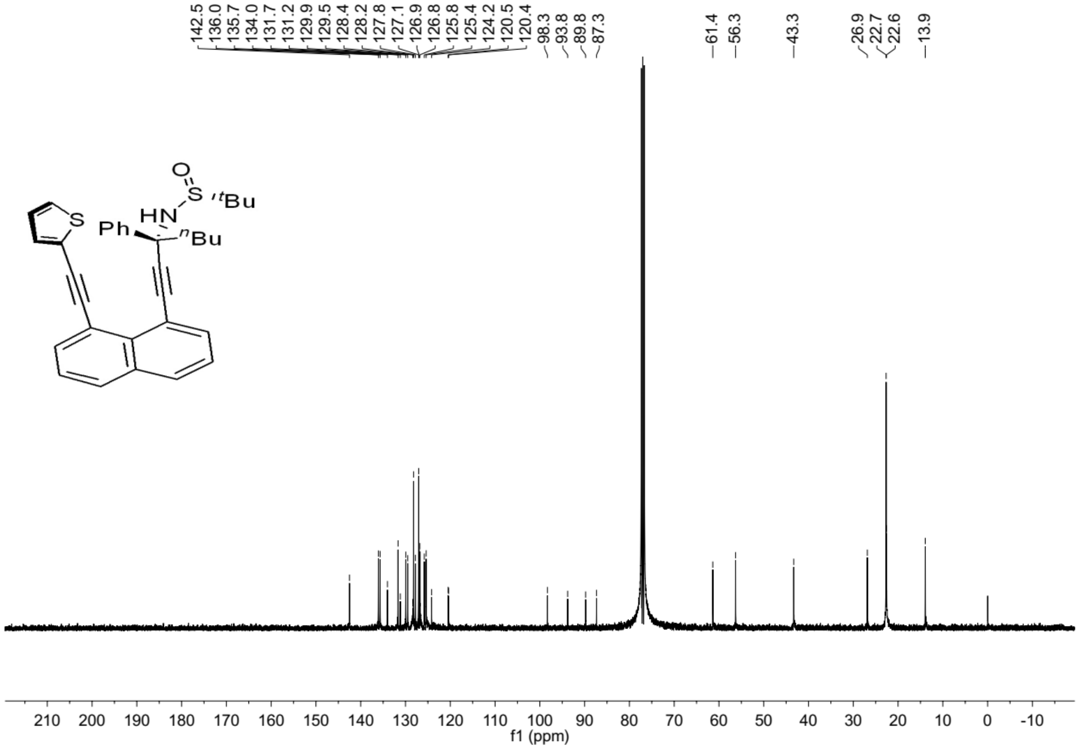

Figure S68. **^{13}C NMR Spectrum of Compound 7ac ($CDCl_3$, 100 MHz)**

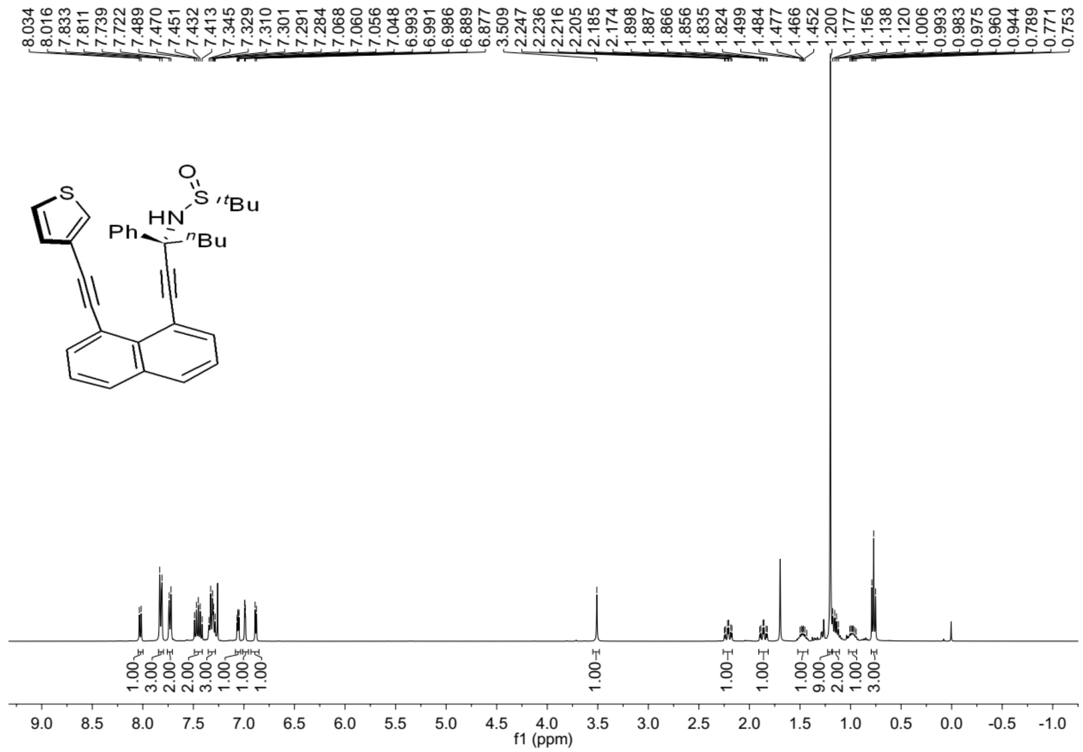

Figure S69. ^{1}H NMR Spectrum of Compound 7ad ($CDCl_3$, 400 MHz)

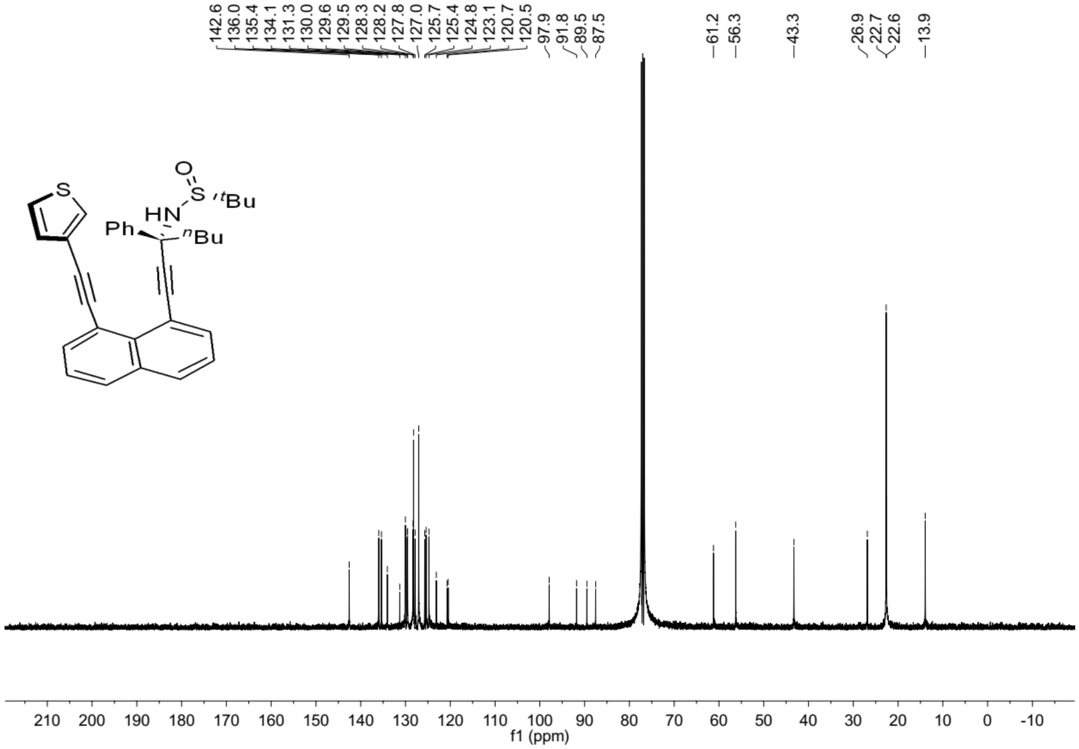

Figure S70. ^{13}C NMR Spectrum of Compound 7ad ($CDCl_3$, 100 MHz)

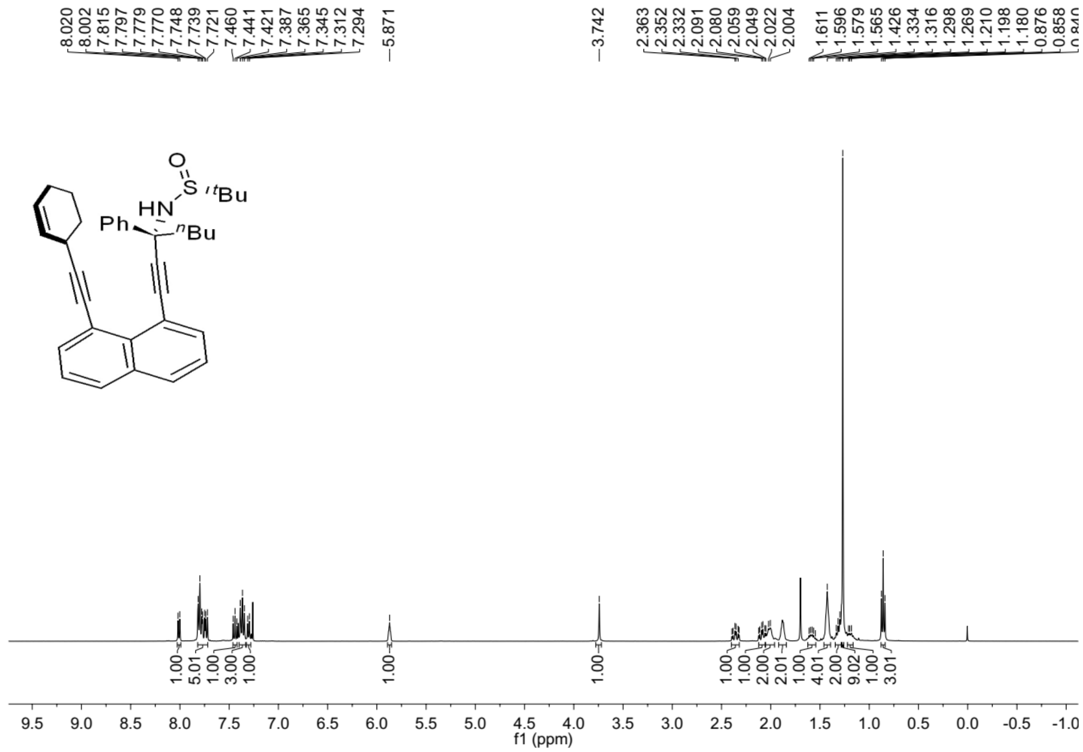

Figure S71. ^{1}H NMR Spectrum of Compound 7ae ($CDCl_3$, 400 MHz)

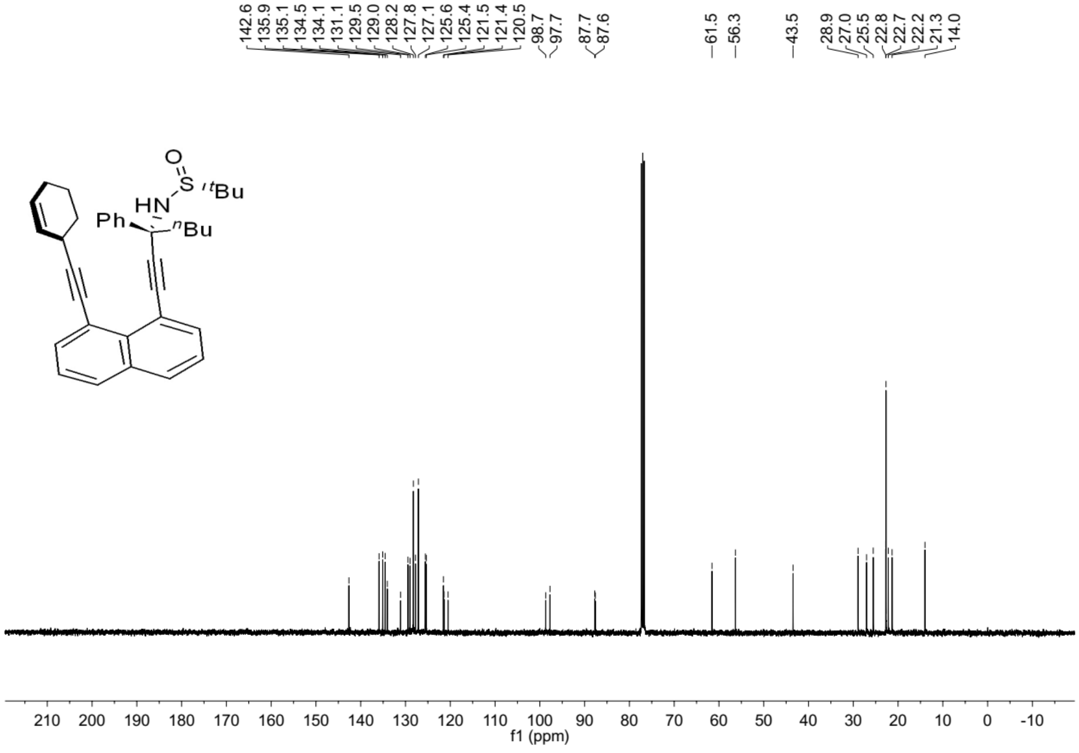

Figure S72. ^{13}C NMR Spectrum of Compound 7ae ($CDCl_3$, 100 MHz)

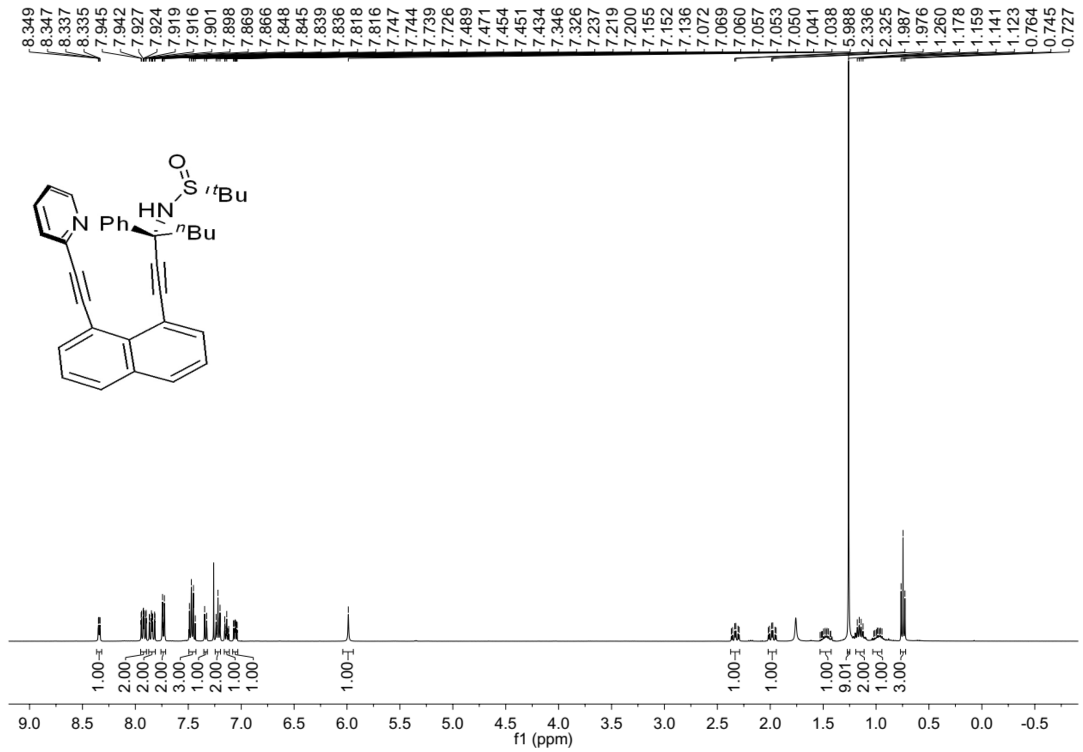

Figure S73. ^{1}H NMR Spectrum of Compound 7af ($CDCl_3$, 400 MHz)

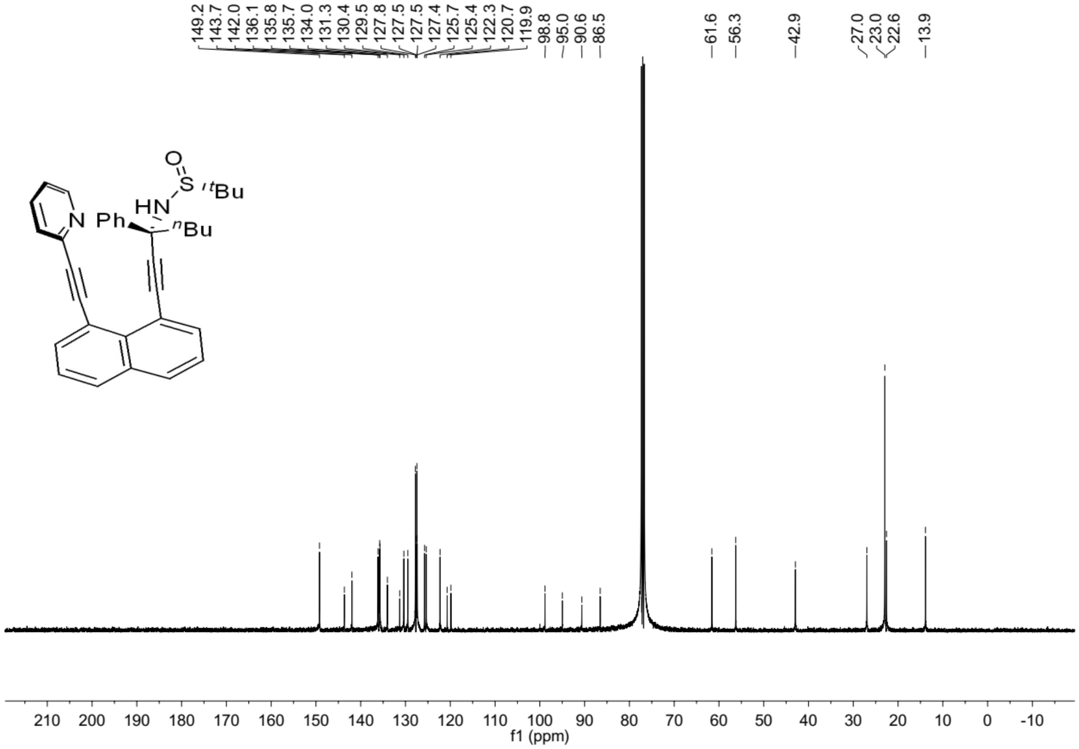

Figure S74. ^{13}C NMR Spectrum of Compound 7af ($CDCl_3$, 100 MHz)

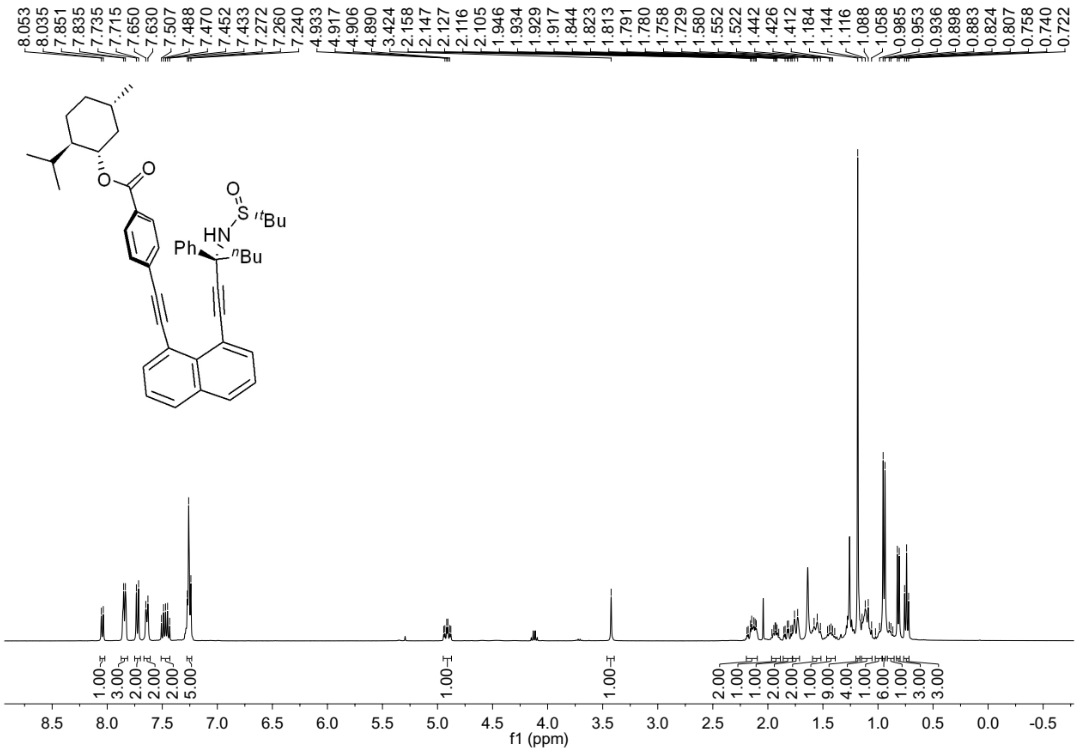

Figure S75. ^{1}H NMR Spectrum of Compound 7ag ($CDCl_3$, 400 MHz)

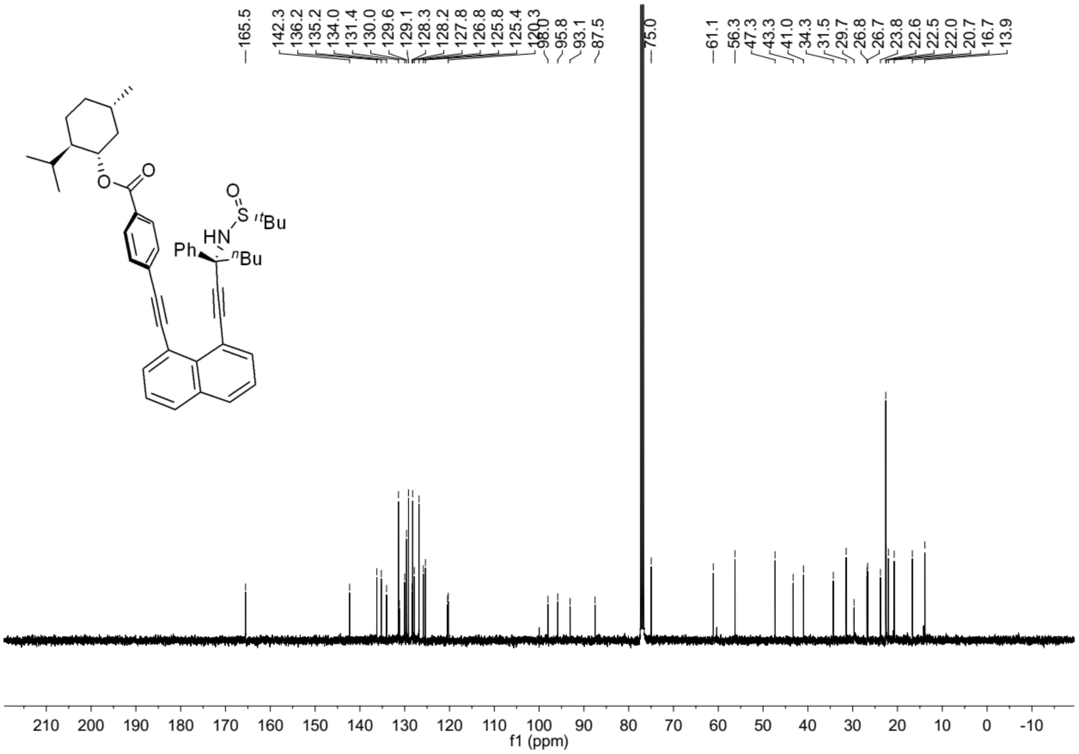

Figure S76. ^{13}C NMR Spectrum of Compound 7ag ($CDCl_3$, 100 MHz)

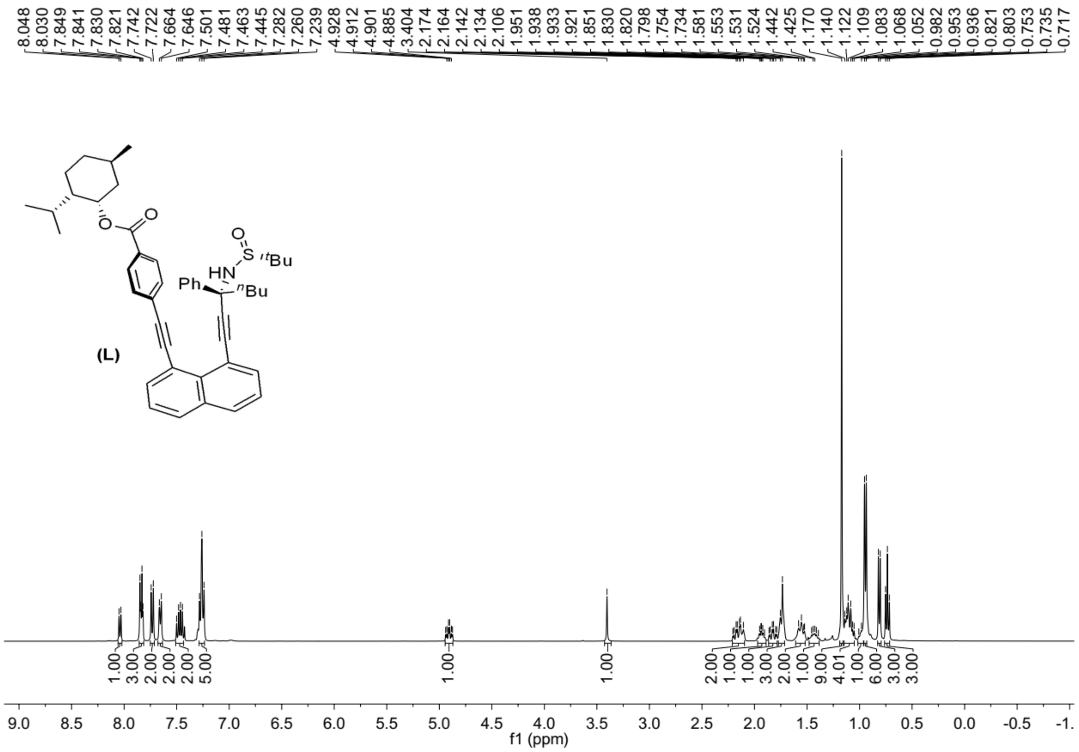

Figure S77. ^{1}H NMR Spectrum of Compound 7ah (CDCl$_3$, 400 MHz)

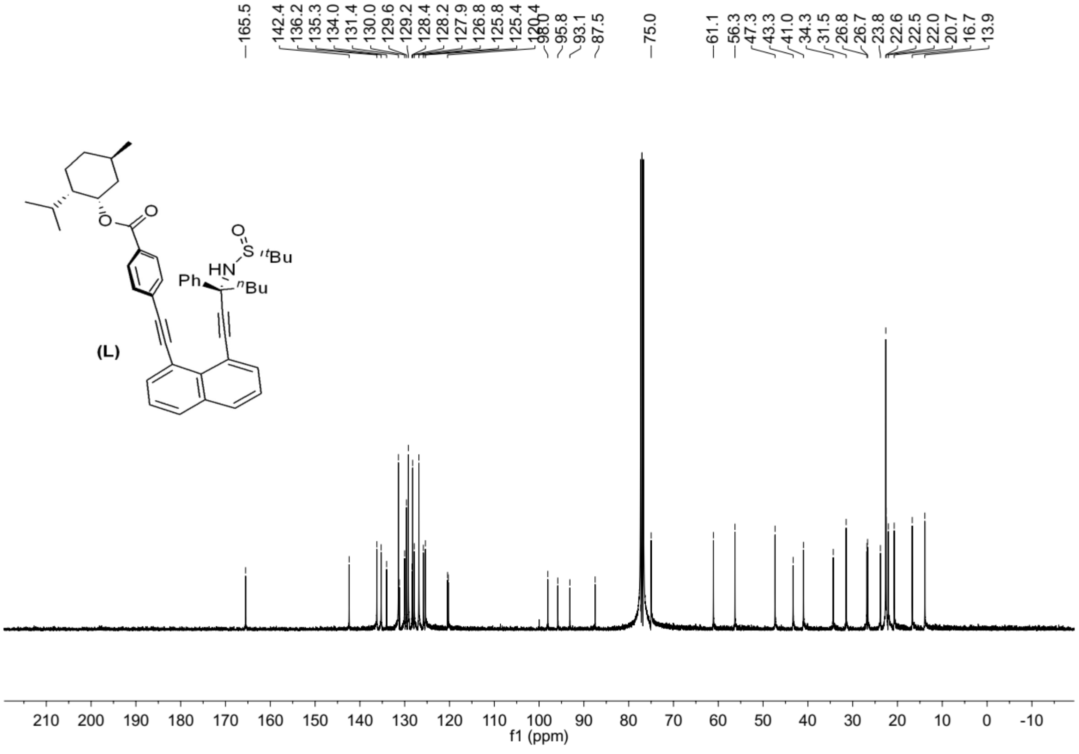

Figure S78. ^{13}C NMR Spectrum of Compound 7ah (CDCl$_3$, 100 MHz)

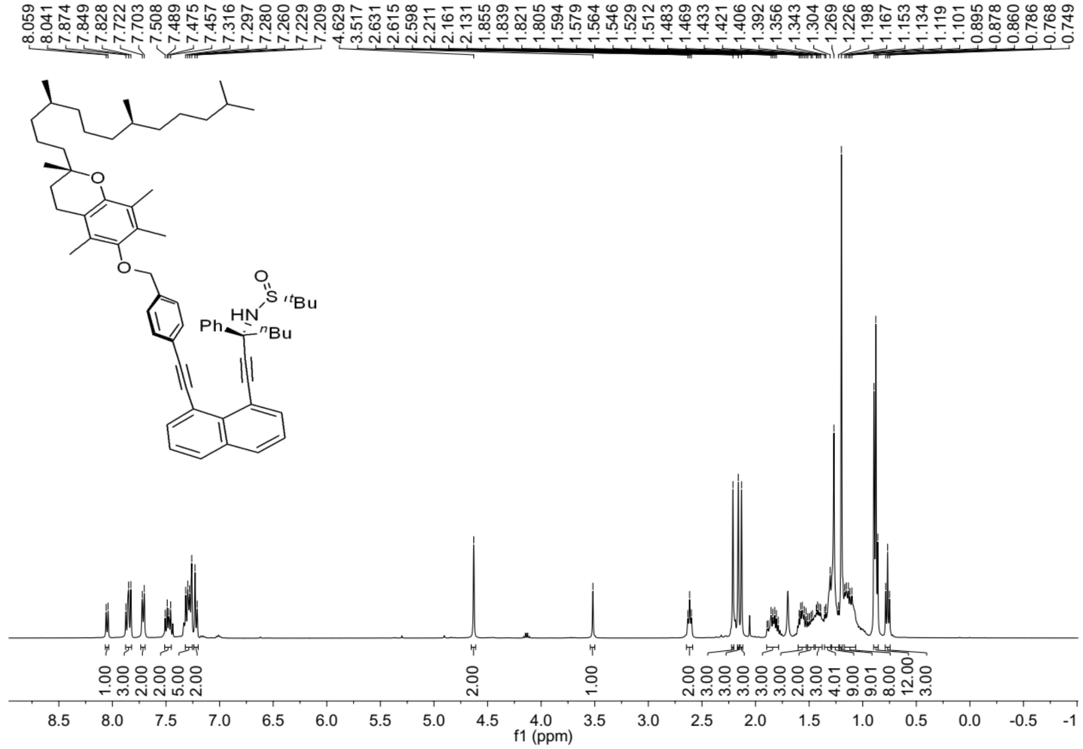

Figure S79. ^{1}**H NMR Spectrum of Compound 7ai (CDCl$_3$, 400 MHz)**

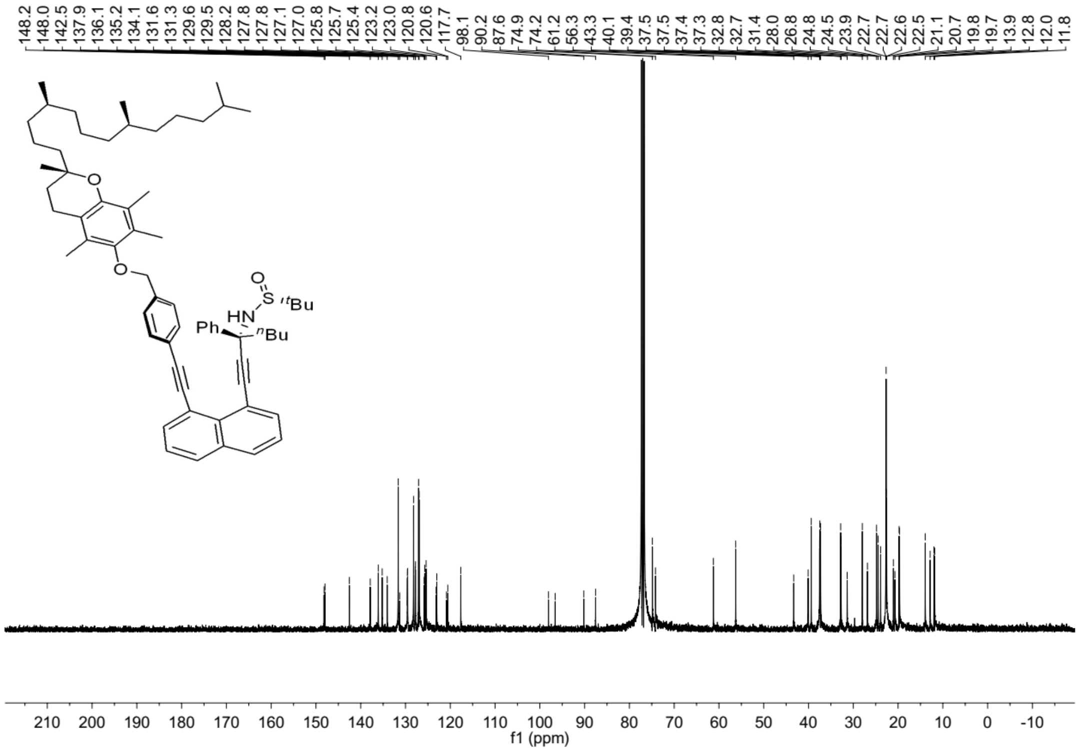

Figure S80. ^{13}**C NMR Spectrum of Compound 7ai (CDCl$_3$, 100 MHz)**

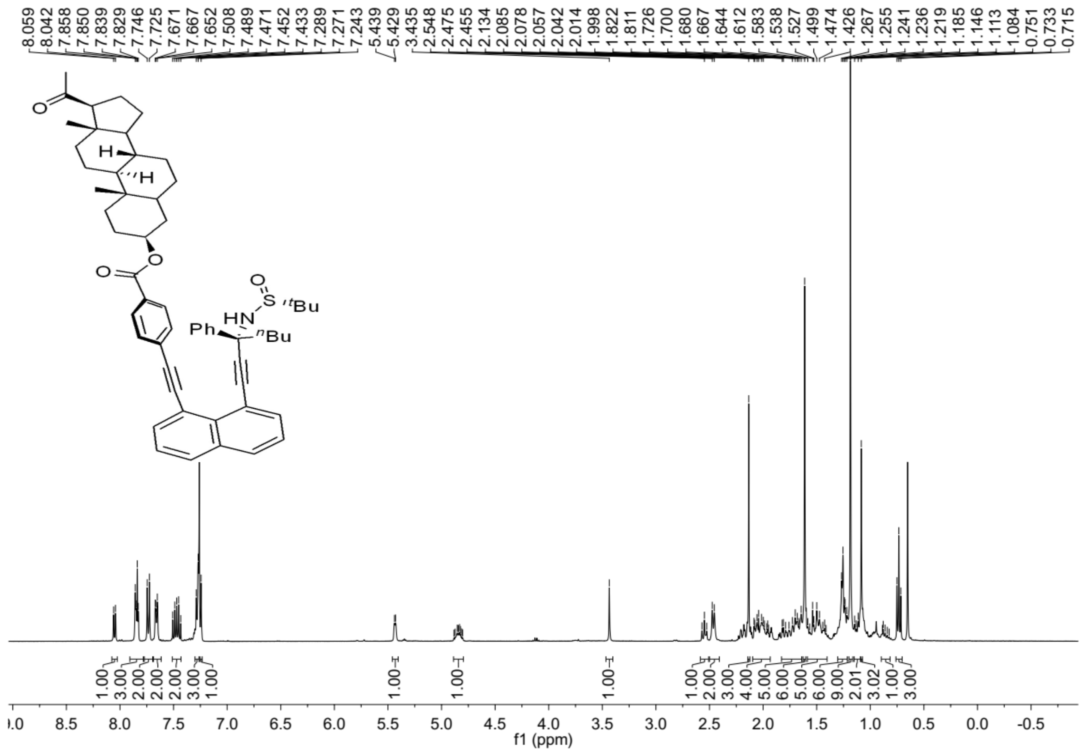

Figure S81. ^{1}H NMR Spectrum of Compound 7aj (CDCl$_3$, 400 MHz)

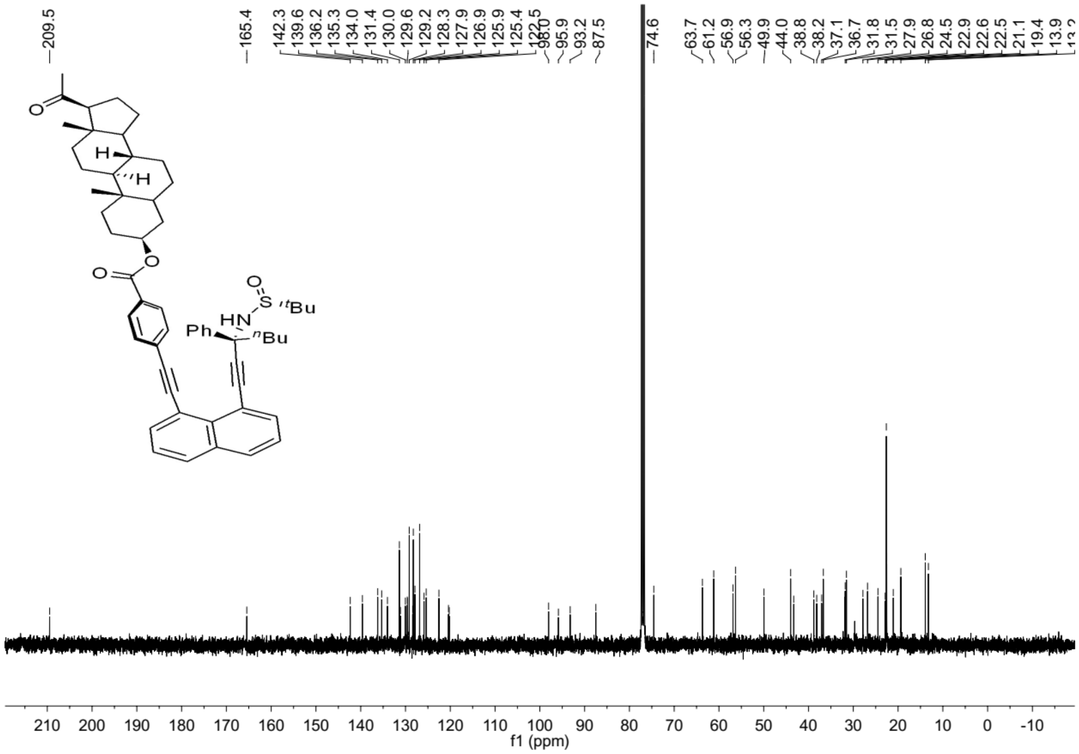

Figure S82. ^{13}C NMR Spectrum of Compound 7aj (CDCl$_3$, 100 MHz)

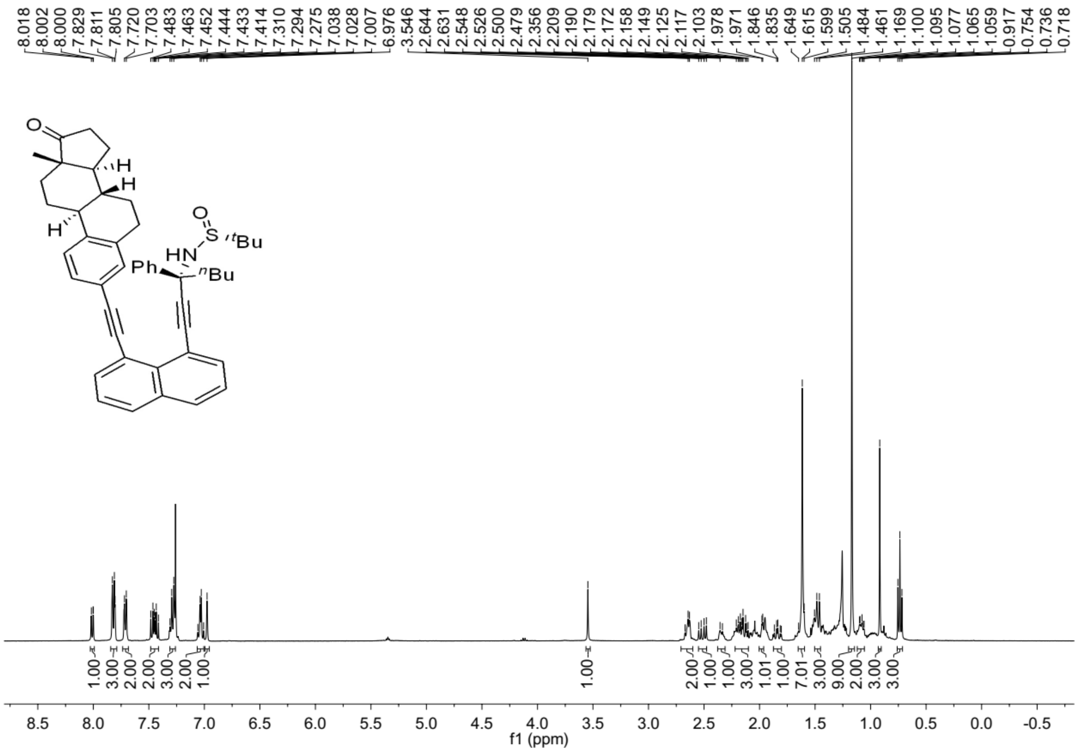

Figure S83. ^{1}H NMR Spectrum of Compound 7ak ($CDCl_3$, 400 MHz)

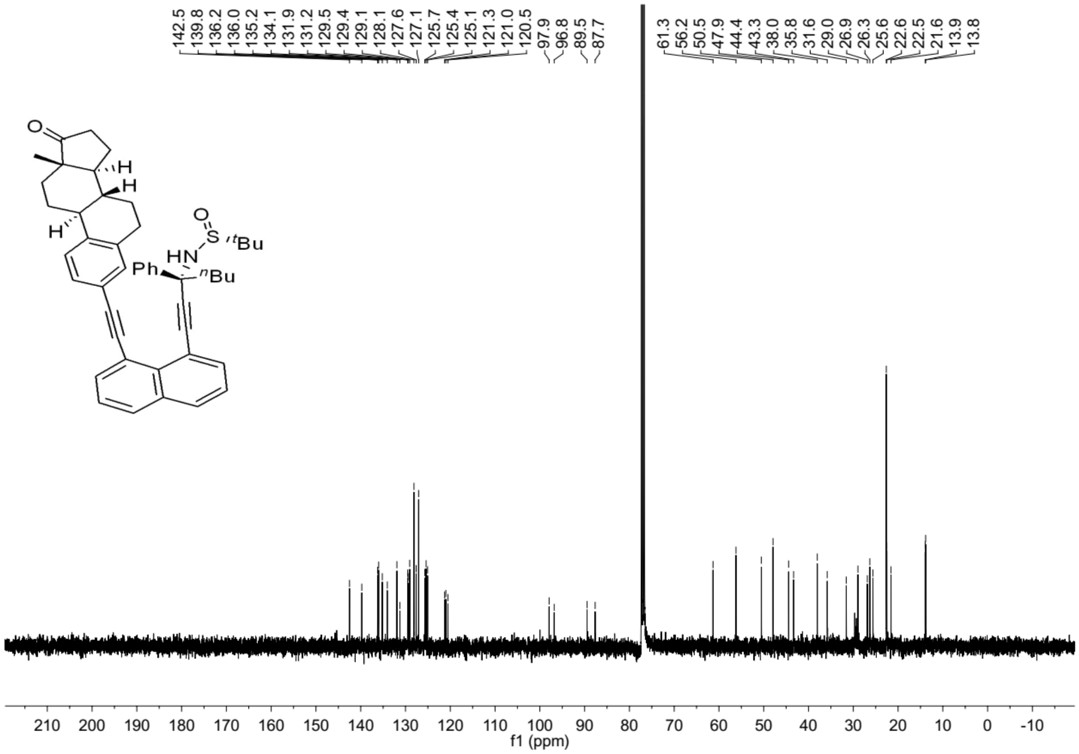

Figure S84. ^{13}C NMR Spectrum of Compound 7ak ($CDCl_3$, 100 MHz)

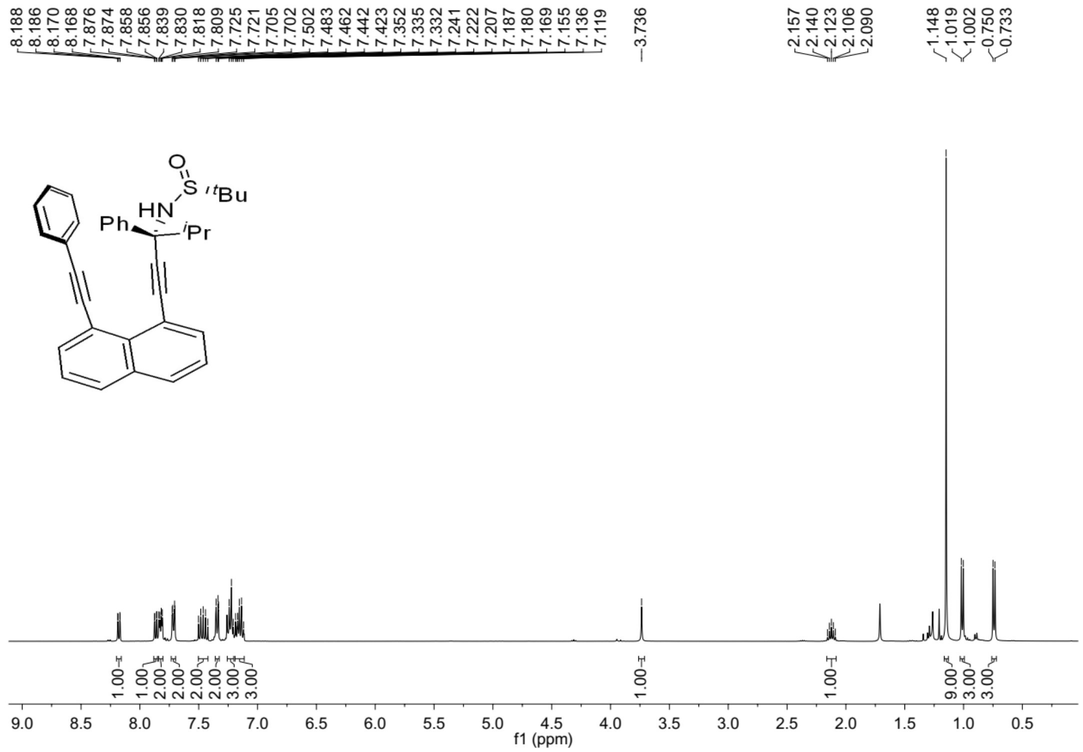

Figure S85. ^{1}**H NMR Spectrum of Compound 7ba (CDCl$_3$, 400 MHz)**

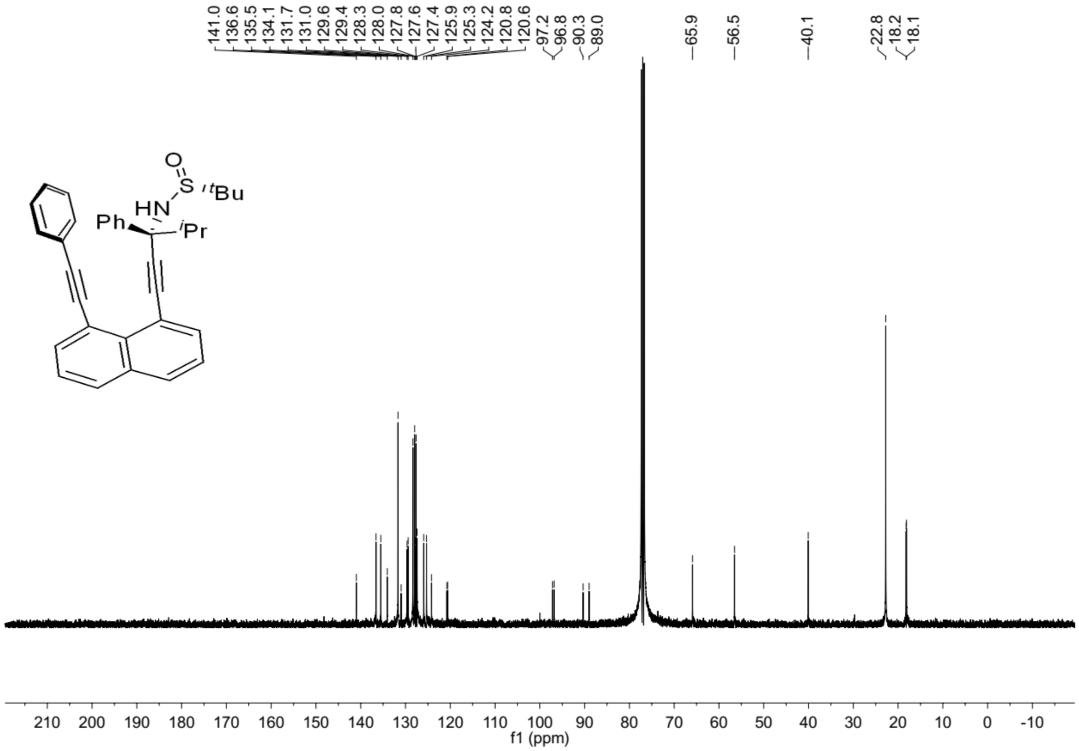

Figure S86. ^{13}**C NMR Spectrum of Compound 7ba (CDCl$_3$, 100 MHz)**

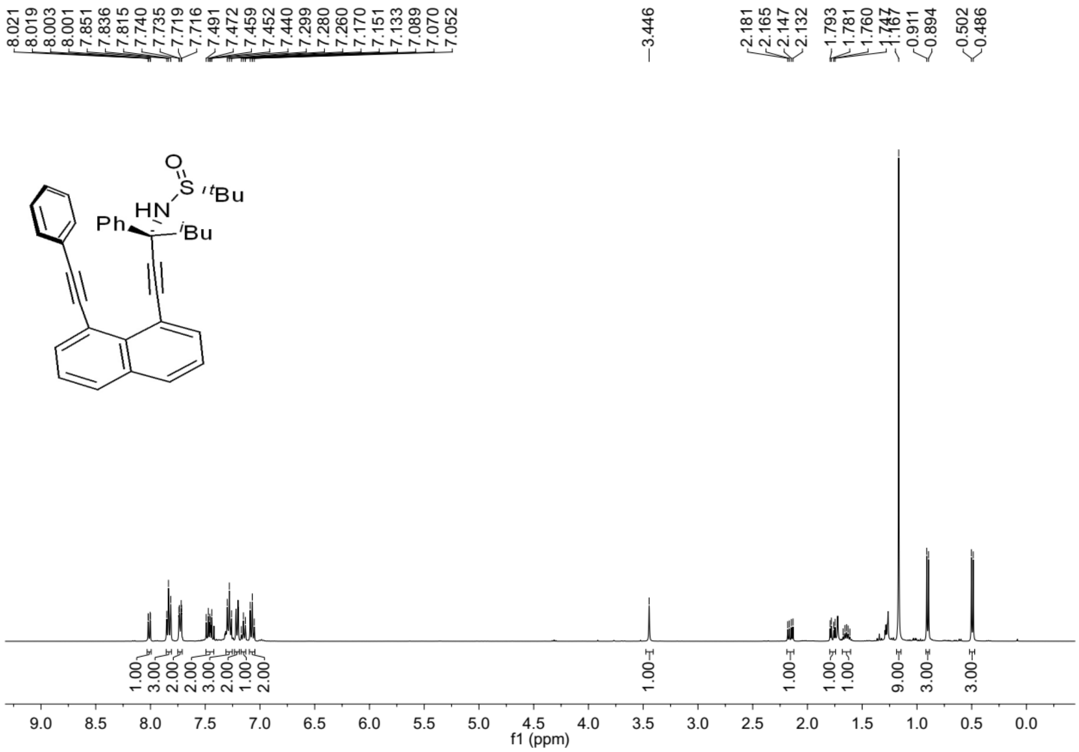

Figure S87. ^{1}H **NMR Spectrum of Compound 7bb (CDCl$_3$, 400 MHz)**

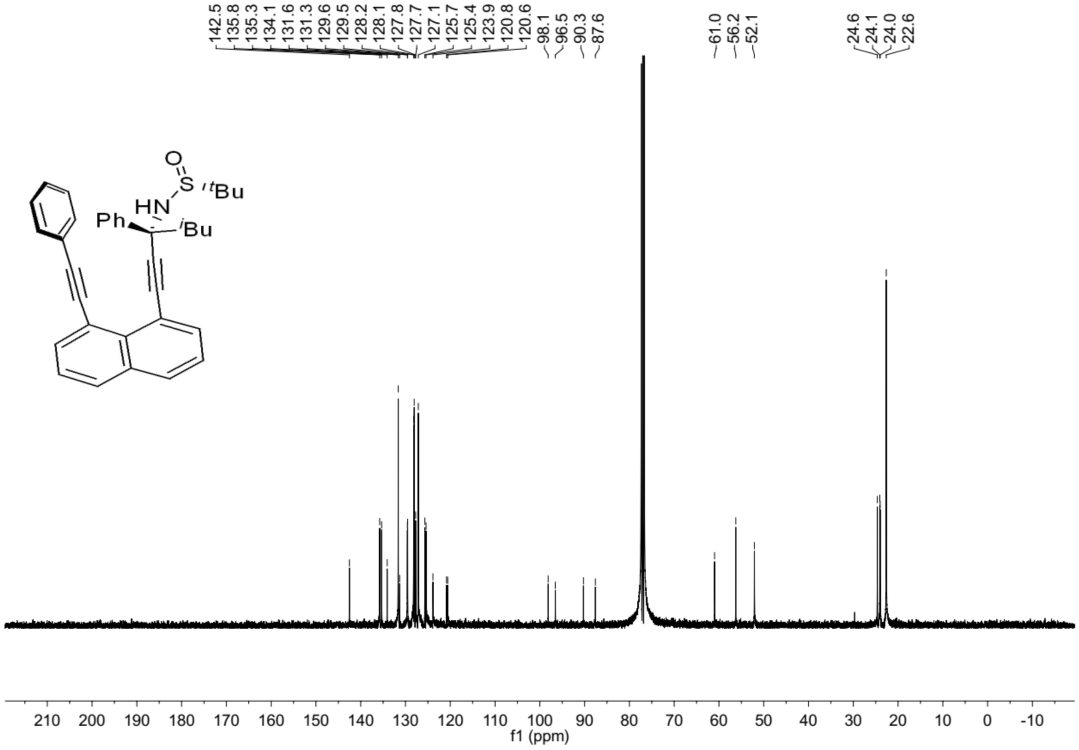

Figure S88. ^{13}C **NMR Spectrum of Compound 7bb (CDCl$_3$, 100 MHz)**

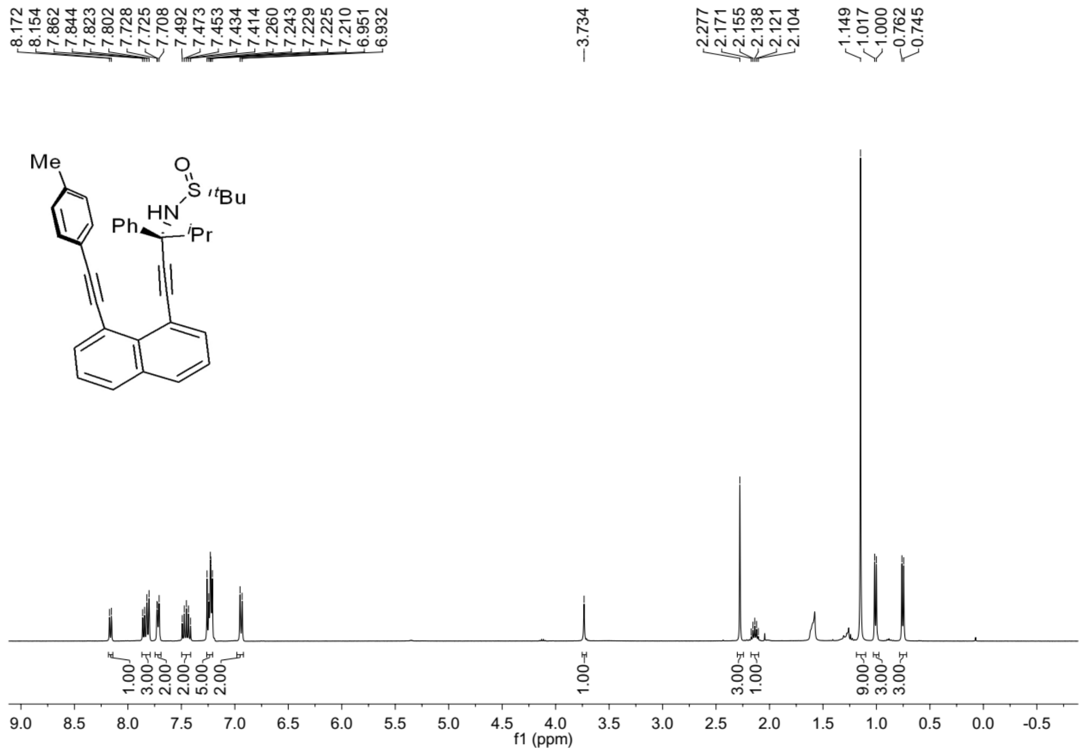

Figure S89. ^{1}H NMR Spectrum of Compound 7bc ($CDCl_3$, 400 MHz)

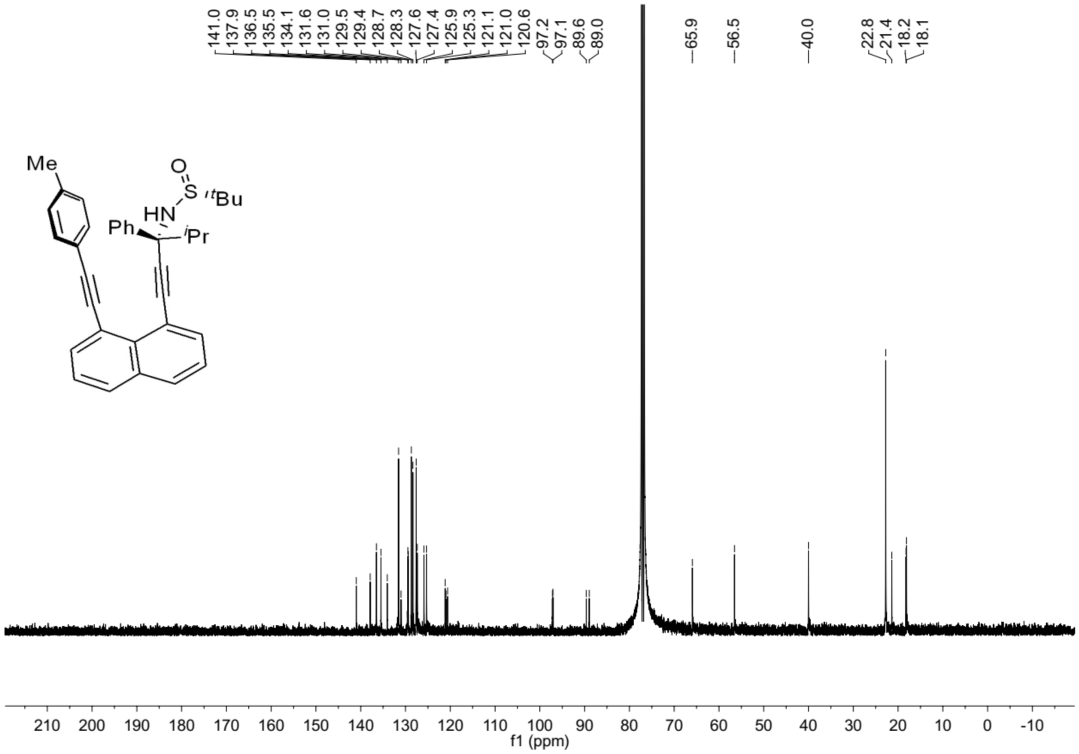

Figure S90. ^{13}C NMR Spectrum of Compound 7bc ($CDCl_3$, 100 MHz)

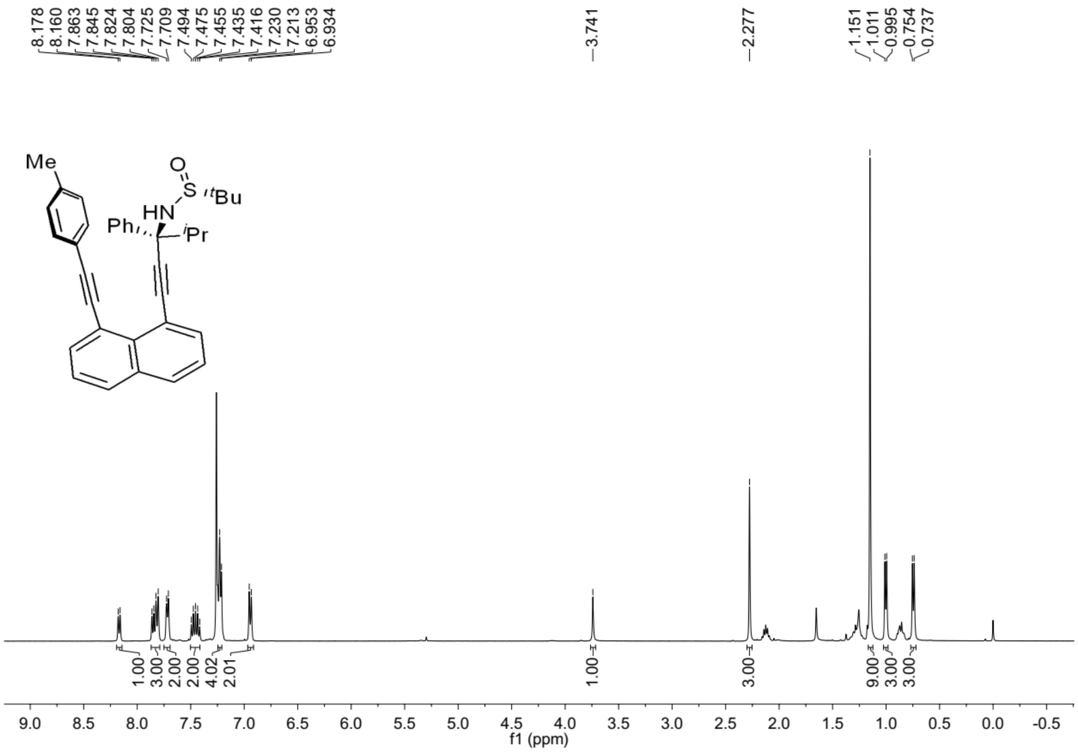

Figure S91. ^{1}H NMR Spectrum of Compound 7bc' ($CDCl_3$, 400 MHz)

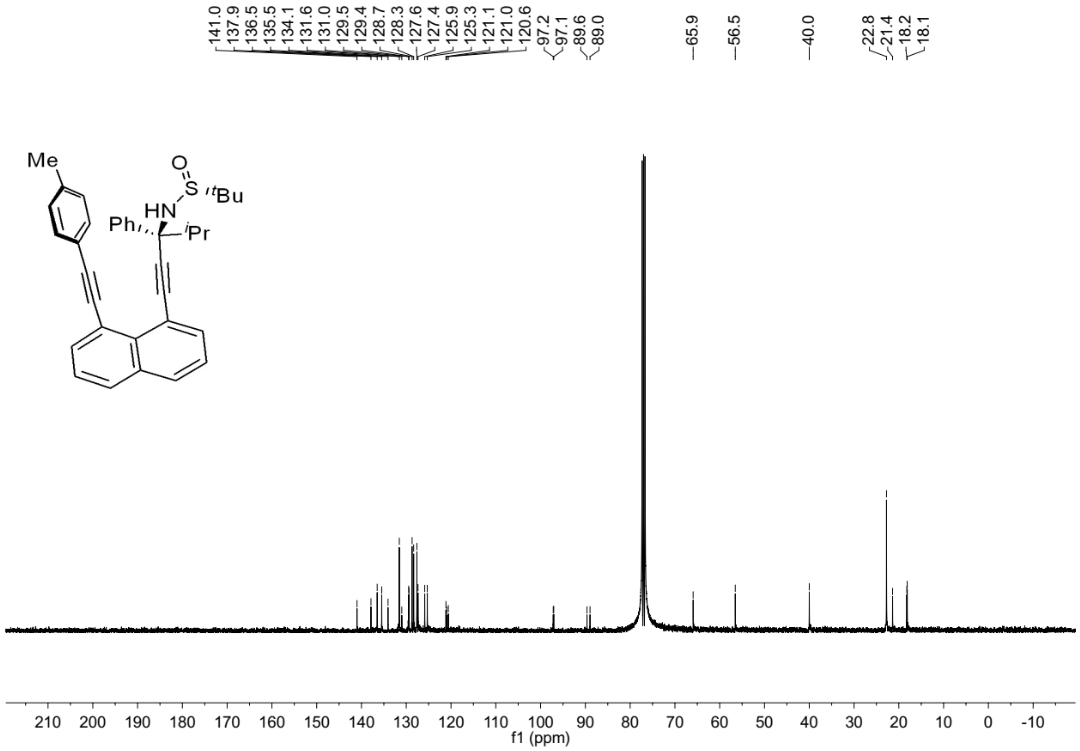

Figure S92. ^{13}C NMR Spectrum of Compound 7bc' ($CDCl_3$, 100 MHz)

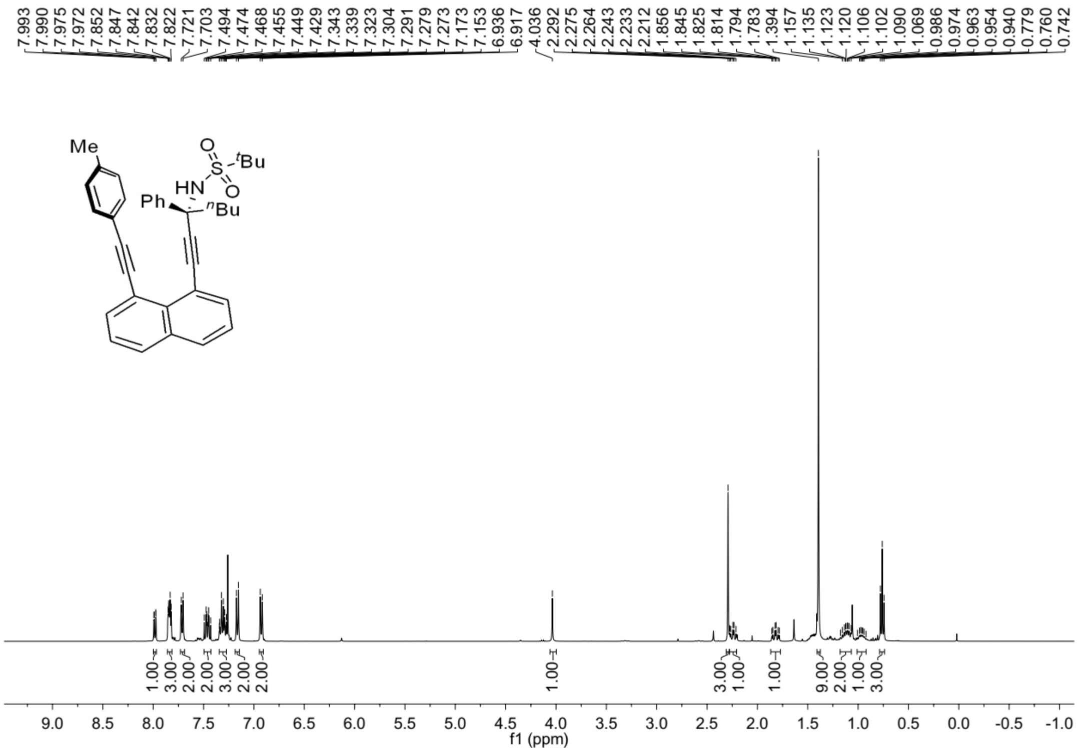

Figure S93. ^{1}H NMR Spectrum of Compound 8a ($CDCl_3$, 400 MHz)

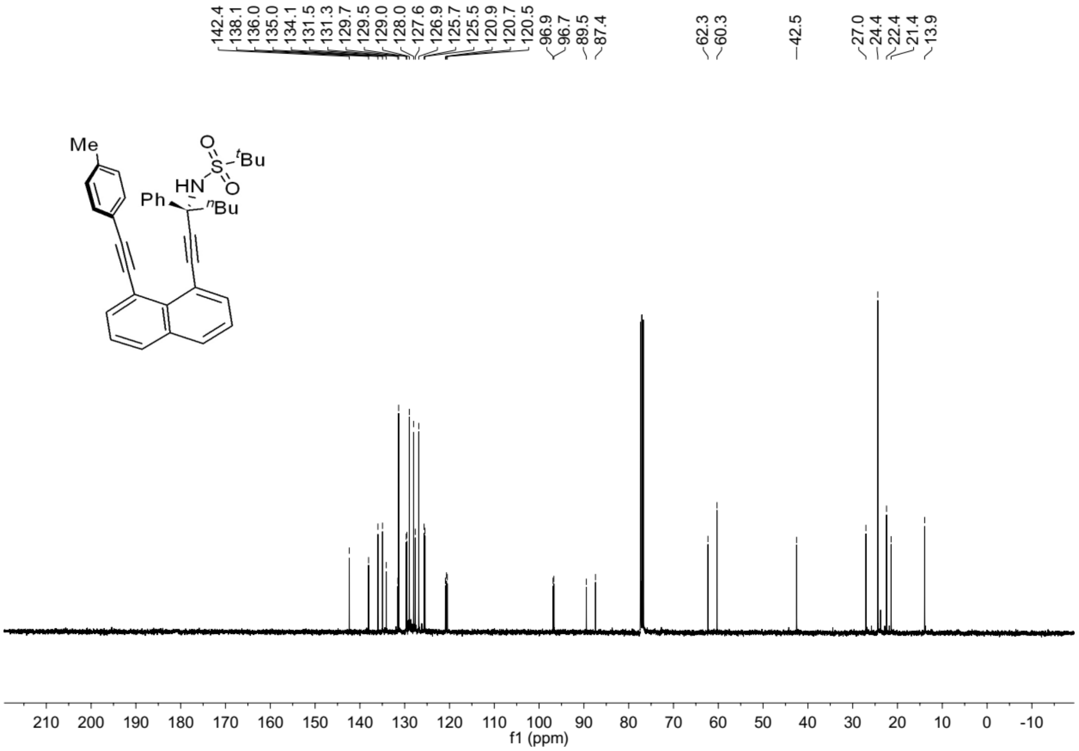

Figure S94. ^{13}C NMR Spectrum of Compound 8a ($CDCl_3$, 100 MHz)

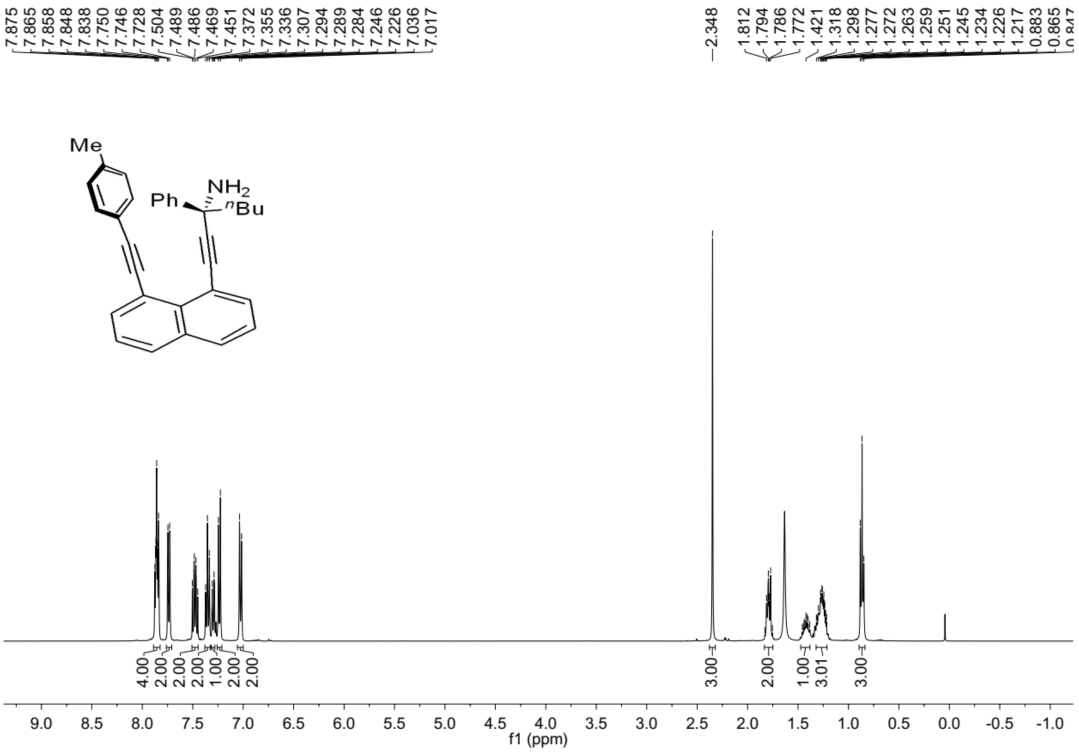

Figure S95. ¹H NMR Spectrum of Precursor 8b ($CDCl_3$, 400 MHz)

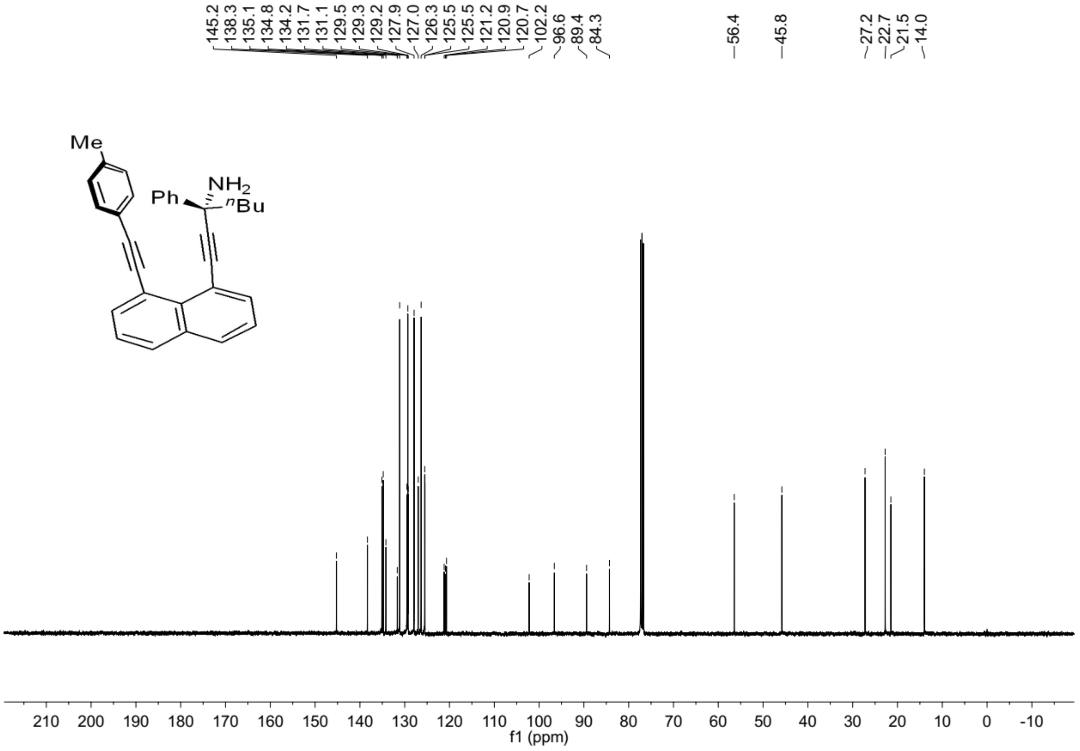

Figure S96. ¹³C NMR Spectrum of Precursor 8b ($CDCl_3$, 100 MHz)

4 VT NMR of Compound **7bc**

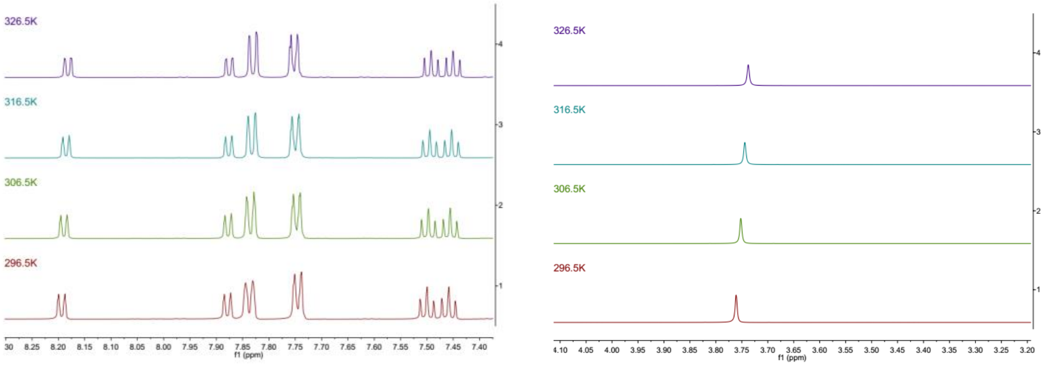

^{1}H VT-NMR spectra of 7bc in $CDCl_3$.

(The spectra above were acquired in 10-degree increments between 25 °C and 55 °C.)

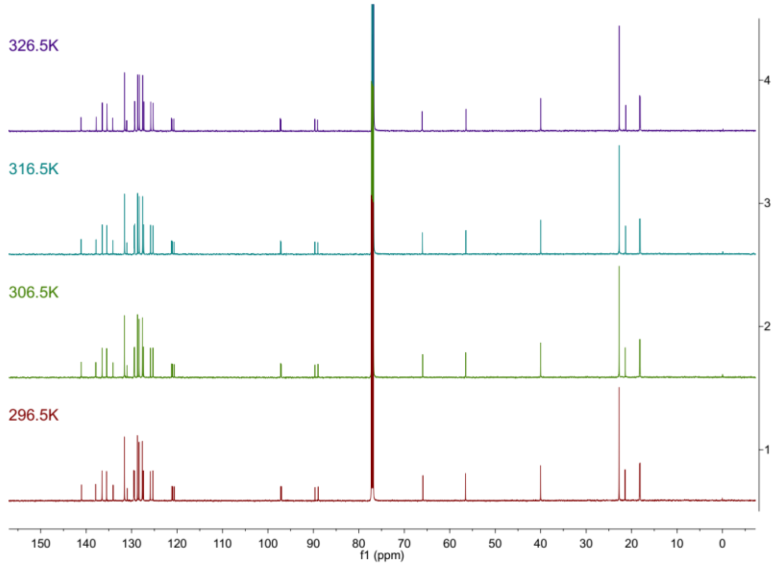

^{13}C VT-NMR spectra of 7bc in $CDCl_3$.

(The spectra above were acquired in 10-degree increments between 25 °C and 55 °C.)

According to DFT studies, when increasing the temperature, one of the conformations of **7bc-2**, **7bc-3** may be observed. In **7bc-3**, the isopropyl group is closer to the left aromatic ring and may be subject to the shielding effect of this aromatic ring, leading to a lower field shift in the H chemical shift of isopropyl group. After undergoing **VT-NMR**, we can clearly see that whether it is the chemical shift of H or the chemical shift of C, all peaks remain consistent, indicating that the structure shown in the **7bc** is the most stable isomer.

5 X-ray Single-crystal Data for Compound **7bc and 7bc'**

Table S1 Crystal data and structure refinement of **7bc**.

Identification code	CCDC 2303102
Empirical formula	$C_{35}H_{35}NOS$
Formula weight	517.70
Temperature/K	298(2)
Crystal system	trigonal
Space group	$P3_2$
a/Å	13.6015(14)
b/Å	13.6015(14)
c/Å	13.3008(15)
α/°	90.00
β/°	90.00
γ/°	120.00
Volume/Å^3	2131.0(4)
Z	3
ρ_{calc}g/cm^3	1.210
μ/mm^{-1}	0.142
F(000)	828.0
Crystal size/mm^3	0.4 × 0.11 × 0.1
Radiation	MoKα (λ = 0.71073)
2Θ range for data collection/°	4.62 to 50.04
Index ranges	$-16 \le h \le 11$, $-16 \le k \le 16$, $-15 \le l \le 15$
Reflections collected	10359
Independent reflections	4970 [R_{int} = 0.0681, R_{sigma} = 0.1029]

Data/restraints/parameters	4970/1/378
Goodness-of-fit on F^2	1.013
Final R indexes [I>=2σ (I)]	R_1 = 0.0592, wR_2 = 0.1188
Final R indexes [all data]	R_1 = 0.0938, wR_2 = 0.1299
Largest diff. peak/hole / e $Å^{-3}$	0.17/-0.24
Flack parameter	0.03(10)

Table S2 Crystal data and structure refinement of **7bc’**.

Identification code	CCDC 2303103
Empirical formula	$C_{35}H_{35}NOS$
Formula weight	517.70
Temperature/K	298(2)
Crystal system	trigonal
Space group	$P3_1$
a/Å	13.5845(12)
b/Å	13.5845(12)
c/Å	13.2817(11)
α/°	90.00
β/°	90.00
γ/°	120.00
Volume/$Å^3$	2122.6(3)
Z	3
$\rho_{calc}g/cm^3$	1.215
μ/mm^{-1}	0.143
F(000)	828.0

Crystal size/mm^3	0.3 × 0.22 × 0.14
Radiation	MoKα (λ = 0.71073)
2Θ range for data collection/°	4.62 to 50.04
Index ranges	-15 ≤ h ≤ 16, -14 ≤ k ≤ 16, -15 ≤ l ≤ 15
Reflections collected	10284
Independent reflections	4799 [R_{int} = 0.0386, R_{sigma} = 0.0549]
Data/restraints/parameters	4799/1/350
Goodness-of-fit on F^2	1.092
Final R indexes [I>=2σ (I)]	R_1 = 0.0445, wR_2 = 0.0959
Final R indexes [all data]	R_1 = 0.0703, wR_2 = 0.1122
Largest diff. peak/hole / e $Å^{-3}$	0.15/-0.26
Flack parameter	-0.06(9)
